# Discovery of Mcl-1-specific inhibitor AZD5991 and preclinical activity in multiple myeloma and acute myeloid leukemia

**DOI:** 10.1038/s41467-018-07551-w

**Published:** 2018-12-17

**Authors:** Adriana E. Tron, Matthew A. Belmonte, Ammar Adam, Brian M. Aquila, Lawrence H. Boise, Elisabetta Chiarparin, Justin Cidado, Kevin J. Embrey, Eric Gangl, Francis D. Gibbons, Gareth P. Gregory, David Hargreaves, J. Adam Hendricks, Jeffrey W. Johannes, Ricky W. Johnstone, Steven L. Kazmirski, Jason G. Kettle, Michelle L. Lamb, Shannon M. Matulis, Ajay K. Nooka, Martin J. Packer, Bo Peng, Philip B. Rawlins, Daniel W. Robbins, Alwin G. Schuller, Nancy Su, Wenzhan Yang, Qing Ye, Xiaolan Zheng, J. Paul Secrist, Edwin A. Clark, David M. Wilson, Stephen E. Fawell, Alexander W. Hird

**Affiliations:** 1 Oncology, IMED Biotech Unit, AstraZeneca, Waltham, MA 02451 USA; 2 Department of Hematology and Medical Oncology, Emory University School of Medicine, Atlanta, GA 30322 USA; 3 Winship Cancer Institute of Emory University, Atlanta, GA 30322 USA; 4 Oncology, IMED Biotech Unit, AstraZeneca, Cambridge, CB4 0WG UK; 5 Discovery Sciences, IMED Biotech Unit, AstraZeneca, Cambridge, CB4 0WG UK; 6 School of Clinical Sciences at Monash Health, Monash University, Clayton, VIC 3800 Australia; 7 Peter MacCallum Cancer Centre, Melbourne, VIC 3000 Australia; 8 Discovery Sciences, IMED Biotech Unit, AstraZeneca, Waltham, MA 02451 USA; 9 The Sir Peter MacCallum Center, Department of Oncology, University of Melbourne, Parkville, VIC 3000 Australia; 10 Oncology, IMED Biotech Unit, AstraZeneca, Alderley Park, SK10 4TG UK; 11 Pharmaceutical Sciences, IMED Biotech Unit, AstraZeneca, Waltham, MA 02451 USA; 12 Present Address: LifeMine Therapeutics, Cambridge, MA USA; 13 Present Address: Surface Oncology, Cambridge, MA USA; 14 Present Address: Alkermes, Inc., Waltham, MA USA; 15 Present Address: Fulcrum Therapeutics, Cambridge, MA USA; 16 Present Address: Nurix, Inc., San Francisco, CA USA

## Abstract

Mcl-1 is a member of the Bcl-2 family of proteins that promotes cell survival by preventing induction of apoptosis in many cancers. High expression of Mcl-1 causes tumorigenesis and resistance to anticancer therapies highlighting the potential of Mcl-1 inhibitors as anticancer drugs. Here, we describe AZD5991, a rationally designed macrocyclic molecule with high selectivity and affinity for Mcl-1 currently in clinical development. Our studies demonstrate that AZD5991 binds directly to Mcl-1 and induces rapid apoptosis in cancer cells, most notably myeloma and acute myeloid leukemia, by activating the Bak-dependent mitochondrial apoptotic pathway. AZD5991 shows potent antitumor activity in vivo with complete tumor regression in several models of multiple myeloma and acute myeloid leukemia after a single tolerated dose as monotherapy or in combination with bortezomib or venetoclax. Based on these promising data, a Phase I clinical trial has been launched for evaluation of AZD5991 in patients with hematological malignancies (NCT03218683).

## Abstract (web summary)

High expression of Mcl-1 promotes tumorigenesis and resistance to anticancer therapies. Here they report a macrocyclic molecule with high selectivity and affinity for Mcl-1 that exhibits potent anti-tumor effects as single agent and in combination with bortezomib or venetoclax in preclinical models of multiple myeloma and acute myeloid leukemia.

## Introduction

Apoptosis is a highly regulated program of cell death critical for normal development and tissue homeostasis. Impaired apoptosis plays a major role in cancer development and underpins resistance to conventional cytotoxic as well as targeted therapies^1–3^. Three subsets of Bcl-2 proteins interact to determine whether cells commit to apoptosis. The signaling cascade is initiated by upregulation of pro-apoptotic BH3-only Bcl-2 proteins (for example, Bim, Bid, Puma, Noxa) in response to cellular stresses, such as DNA damage or oncogene activation. The BH3-only proteins then associate with anti-apoptotic Bcl-2 relatives (Mcl-1, Bcl-2, Bcl-xL, Bcl-w, Bfl-1/A1, Bcl-b) preventing their binding and inactivation of Bak and Bax (effector Bcl-2 proteins) which can then form oligomeric pores at the outer mitochondrial membrane causing cytochrome c release and caspase activation. Thus, the balance between pro-apoptotic and anti-apoptotic Bcl-2 proteins determines the onset of apoptosis and cell death.

Although the pro-survival Bcl-2 family members share several functions and structural features, the distinctive regulation of Mcl-1 makes this anti-apoptotic protein unique. In contrast to other anti-apoptotic Bcl-2 proteins, Mcl-1 has a large unstructured amino-terminus core that contains multiple phosphorylation, ubiquitination^4^ and caspase cleavage^5,6^ sites that tightly control Mcl-1’s short protein half-life (1–4 h)^7^, fine-tuning its activity in response to pro-apoptotic and anti-apoptotic stimuli^8^.

*MCL1* is within one of the most frequently amplified gene regions in human cancers^9^ and its expression is often associated with resistance to cytotoxic agents and relapse in patients^10^. Several tumor types have been described as being dependent on Mcl-1, in particular multiple myeloma (MM)^11^, acute myeloid leukemia (AML)^12^, chronic myeloid leukemia^13^, B-cell acute lymphoblastic leukemia^14^, hepatocellular carcinoma^15^, and certain non-small cell lung cancers^16^. Mcl-1 also drives innate and acquired resistance to several cytotoxic agents^17–19^ and targeted therapies, including the Bcl-2 selective inhibitor venetoclax^20,21^. This large body of evidence underscores the potential of Mcl-1 inhibitors as anticancer drugs.

Despite the remarkable interest in developing selective Mcl-1 inhibitors over the past two decades, verified Mcl-1 inhibitors have been slow to enter the clinic [https://ClinicalTrials.gov/show/NCT02675452], [https://ClinicalTrials.gov/show/NCT02979366]. The long shallow hydrophobic protein–protein interaction interface has proven challenging to drug with a small molecule and while many inhibitors have been reported in the literature and even in clinical trials, off-target effects have been shown to drive phenotypic activity for many compounds^22^.

Here, we describe the discovery, mechanism of action, and preclinical efficacy of an Mcl-1 inhibitor, AZD5991, in MM and AML models that support clinical evaluation of AZD5991 in patients with hematological malignancies [https://ClinicalTrials.gov/show/NCT03218683].

## Results

### Discovery of macrocyclic Mcl-1 inhibitors

**Fig. 1:**
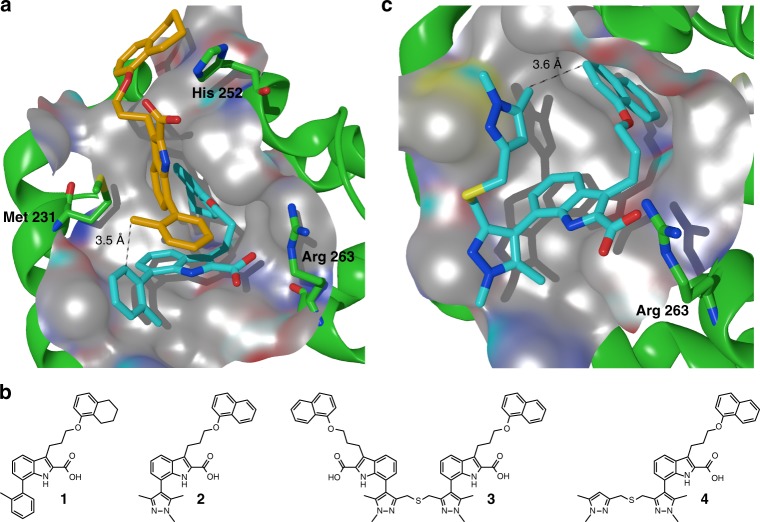
Structure-based design of macrocyclic inhibitors of Mcl-1. **a** Crystal structure of **1** in complex with Mcl-1. Note 2:1 ligand:protein stoichiometry in binding site (pdb id: 6FS2). The protein surface around the highlighted residues has been hidden for clarity. **b** Literature compounds **1** and **2**, dimeric target **3** and compound synthesized from byproduct, **4**. **c** Crystal structure of **4** in complex with Mcl-1 (pdb id: 6FS1). The protein surface and alpha helix ribbon at the front of the pocket has been hidden for clarity

Given the known challenges of designing a small molecule inhibitor for Mcl-1, we initiated multiple parallel lead generation strategies, including (i) fragment-based lead generation (FBLG), (ii) identification from a DNA-encoded library (DEL) screen^23^, (iii) building from known literature compounds, including a new mode of covalent inhibition^24^, and (iv) using structure-based drug design (SBDD). One avenue began with analysis of a series of indole-2-carboxylic acids which have been reported by others^25–27^. Investigating one such literature compound, **1**, we were able to obtain a co-crystal structure in complex with Mcl-1 (Fig. 1a). Surprisingly, we observed two inhibitors bound to the BH3-binding domain of Mcl-1. The first high-affinity binding (cyan molecule in Fig. 1a) overlays well with reported crystal structures^27^, with the 2-carboxylic acid forming an ionic interaction with Arg263 of Mcl-1 (dotted line) and the naphthyl group occupying an induced-fit pocket. The second molecule, with lower affinity-binding mode (orange molecule in Fig. 1a), binds in close proximity to the first molecule, with the methyl group of the 2-toluyl substituent of the second molecule only 3.5 Å from the 6-carbon of the 2-toluyl substituent of the first molecule (solid line). To our knowledge, this 2:1 stoichiometry has not been observed previously with this series of compounds and results in a conformational change in Mcl-1 protein residues (e.g., Met231 side chain and larger movement in the Leu246 to Asp256 helix) to enlarge the binding pocket and accommodate the second binding molecule. 2D protein-observed NMR for a related compound, **2** (Fig. 1b)^26^, also demonstrated two binding events (binding event 1 *K*_d_ < 5 µM, binding event 2 *K*_d_ = 140 ± 8 µM) (Supplementary Fig. 1a–b), with the second weaker binding event supporting the fact that the crystal structure showed no additional strong polar interactions for the second molecule.

Since this larger pocket may be more druggable, we hypothesized that a new hybrid molecule able to take advantage of both binding events would have vastly improved potency compared to **1** or **2** (Mcl-1 TR-FRET IC_50_s = 1.1 and 0.29 µM, respectively). Given the short distance between these molecules, we reasoned that a two-atom linker would be appropriate to link the two monomers. Because the lipophilicity of a dimeric molecule analogous to **1** may lead to solubility that would be too low to allow for in vitro testing, we instead designed a synthetic route to the dimer of **2** in target molecule, **3** (Fig. 1b). Upon synthesis and testing, we found that **3** had no improvement in potency vs. its monomeric congener (Mcl-1 TR-FRET IC_50_ = 0.77 µM for **3** vs. 0.29 µM for **2**).

However, during the synthesis of **3**, we also isolated **4**, resulting from a byproduct in the Suzuki coupling step (see Supplementary Note). We then tested whether **4** could occupy more of the BH3 binding domain of Mcl-1, which should lead to improved potency. Indeed, we observed an improvement in Mcl-1 potency arising from the additional pyrazole substituent (IC_50_ = 0.042 µM). It is known that additional interactions could be made within the BH3-binding groove of Mcl-1^28–30^, but it seemed that the pyrazole substituent would not extend far enough to reach the P4 pocket. A co-crystal structure of **4** in complex with Mcl-1 again revealed movement in several residue sidechains of Mcl-1 to accommodate the terminal pyrazole ring, further opening the induced-fit hydrophobic pocket, similar to what had been observed in the crystal structure of **1**. Interestingly, **4** adopted a “U”-shaped conformation, with the pendant pyrazole 5-methyl group only 3.6 Å from the naphthyl 3-carbon atom (Fig. 1c). We hypothesized that constraining the molecule within a macrocycle would deliver an entropic benefit to potency, if the solution-free conformation of a macrocycle could more closely resemble the bound conformation (c.f., what appeared to be a somewhat unfavorable conformation of the linking atoms in **4**).

Given the hydrophobic nature of the induced-fit pocket and the potential that polar heteroatoms within the linker would pay a desolvation penalty upon binding, we designed molecules with non-polar atoms in the linker. After prioritization of targets based on alignment of docked poses with crystal structures and synthetic tractability, **5** was synthesized in 11 linear steps with an overall yield of 20% from known compounds (see supplementary note). Notably, macrocycle **5** (Mcl-1 FRET IC_50_ = 19 nM) demonstrated improved binding affinity over its acyclic analog, **4** (Mcl-1 FRET IC_50_ = 42 nM).

### Discovery of the macrocyclic inhibitor (*R*_a_)-7

**Fig. 2:**
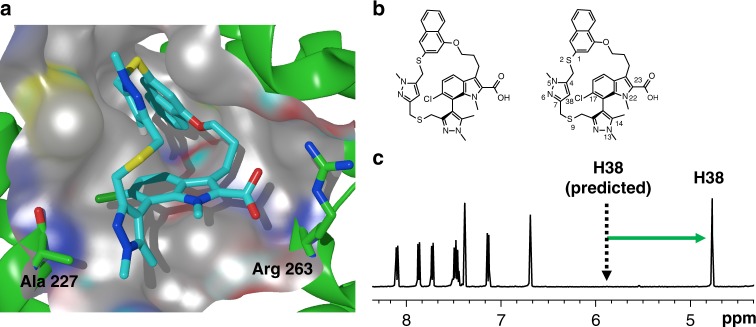
Chemical and crystal structure of (*R*_a_)-**7**. **a** Crystal structure of (*R*_a_)-**7** in complex with Mcl-1. Note coplanar alignment of Arg 263 and the carboxylic acid and the interaction between 17-Cl of (*R*_a_)-**7** and the backbone carbonyl of Ala227 (pdb id: 6FS0). The protein surface around the highlighted residues has been hidden for clarity. **b** Structure of (*R*_a_)-**7** and numbering of atoms. **c** ^1^H NMR of (*R*_a_)-**7**, highlighting the upfield chemical shift of H38 indicative of a conformation that is rigid and consistent with the active binding conformation depicted in **a**

We investigated other substituents within and appended to the macrocyclic ring but ultimately chose to optimize **5**; stepwise improvements led to N-Me indole derivative, **6** (Mcl-1 FRET IC_50_ = 4 nM) and 6-Cl indole derivative, **7** (Mcl-1 FRET IC_50_ < 3 nM) (Table 1 and Supplementary Table 1). A crystal structure of **7** confirmed that the new macrocyclic conformation bound as expected to Mcl-1; only the (*R*_a_)-enantiomer was observed to bind, despite the racemate being used for the co-crystallization (Fig. 2a). The incorporation of both the 6-Cl substituent and indole-1-*N*-Me substituent led to restricted rotation around the biaryl bond and therefore atropisomers could be separated and were stable at room temperature^31^. In addition, the indole-1-*N*-Me forces the carboxylic acid to be orthogonal to the indole, thereby improving the interaction with Arg263 of Mcl-1, whereas the 6-Cl is only 3.2 Å from the backbone carbonyl oxygen of Ala227—likely a favorable halogen–carbonyl bond^32^. The ^1^H NMR chemical shift of the pyrazole 3-H of (*R*_a_)-**7** (H38 in Fig. 2b) was shifted drastically upfield (*δ* 4.75 ppm) vs. the predicted chemical shift from the 2D structure^33^ (*δ* 5.83 ppm, Fig. 2c). This shift was indicative of strong anisotropic shielding and we suspected the macrocyclic structure adopted a rigid conformation in solution. To further explore this, we carried out an extensive NMR solution structural analysis, which demonstrated that (*R*_a_)-**7** adopted a free ligand conformation very similar to the bound conformation observed in the Mcl1 co-crystal structure (see Supplemental Tables 3 and 4 for proton assignment, nOe results and analysis). It was apparent that the upfield chemical shift of H38 was due to its interaction with the pi-cloud of the indole phenyl ring. These data validated the hypothesis of macrocyclic design leading to a dominant bioactive free ligand conformation with improved binding affinity driven by rapid on-rate kinetics (vide infra). As expected, the *R*_a_ enantiomer of **7** was also far more potent, with any activity of the *S*_a_ enantiomer likely a result of residual impurity of (*R*_a_)-**7** ((Sa)-7 was purified by chiral SFC to an enantiomeric excess (e.e.) of >98.8% (see Methods)).

**Table 1:** In vitro activity of Mcl-1 inhibitors

	Compound	2	4	5, R1, R2 = H	6, R1 = Me, R2 = H	(R_a_)-7, R1 = Me, R2 = Cl	(S_a_)-7, R1 = Me, R2 = Cl	8, A-1210477
	Mcl-1 SPR, *K*_d_ (µM)	0.65	0.098	0.047	0.008	0.00017	0.98	0.011
	Mcl-1 FRET, IC_50_ (µM)	0.29	0.042	0.019	0.004	0.0007	6.3	0.006
	MOLP-8, EC_50_ (µM)	>31.5	>31.5	>31.5	0.66	0.033	>16	3.55
	MV4-11, EC_50_ (µM)	>31.5	>20	>31.5	1.05	0.024	>16	1.86
	NCI-H23, EC_50_ (µM)	>31.5	>31.5	nd	5.75	0.19	>25	20.9

### (*R*_a_)-7 is a potent and selective inhibitor of Mcl-1

We then evaluated the selectivity of (*R*_a_)-**7** against pro-survival Bcl-2 family members using FRET assays. (*R*_a_)-**7** was selective for Mcl-1 (IC_50_ 0.72 nM, *K*_i_ = 200 pM) vs. Bcl-2 (IC_50_ = 20 µM, *K*_i_ = 6.8 µM), Bcl-xL (IC_50_ = 36 µM, *K*_i_ = 18 µM), Bcl-w (IC_50_ = 49 µM, *K*_i_ = 25 µM), and Bfl-1 (IC_50_ = 24 µM, *K*_i_ = 12 µM) (Supplementary Fig. 2). Binding of (*R*_a_)-**7** to Mcl-1 was verified by a surface plasmon resonance (SPR) assay which confirmed binding affinity (*K*_d_ = 170 pM, Supplementary Fig. 3a–g) and rapid on-rate binding kinetics (*k*_on_ = 3.8 × 10^6^ M^−1^ s^−1^). It is noteworthy that binding to rodent homologs of Mcl-1 is weaker than to human Mcl-1 with a ~25-fold reduction in *K*_d_ with mouse Mcl-1 and ~4-fold reduction in *K*_d_ with rat Mcl-1, but with equivalent binding affinity to human, dog, and cynomolgus Mcl-1 (SPR). This is consistent with previous reports^30,34^ and caused by differences in the amino acid sequences in the ligand-binding pocket as recently demonstrated by Zhao et al.^34^.

After sub-nanomolar-binding affinity for Mcl-1 was demonstrated, evaluation of activity in cells became a more differentiating factor between molecules; (*R*_a_)-**7** was evaluated vs. **2**, **4**, **5**, **6**, and literature Mcl-1 inhibitor, A-1210477^28^ in a panel of Mcl-1-dependent cell lines. As shown in Table 1, (*R*_a_)-**7** exhibited superior potency across a range of Mcl-1-dependent cell lines. The reduction in potency from binding to cellular assays results from a combination of factors, including the stoichiometry of the Bcl-2 family proteins and how they are complexed to one another within the cell^35^, and physico-chemical properties of AZD5991, such as high plasma protein binding (0.1% free in fetal calf serum). The latter is evident by examining the caspase-inducing activity of AZD5991 in MOLP-8 cells with varying amounts of fetal calf serum in the assay media (EC_50_ = 0.001, 0.008, 0.033 µM in 0%, 2%, and 10% serum conditions, respectively).

**Fig. 3:**
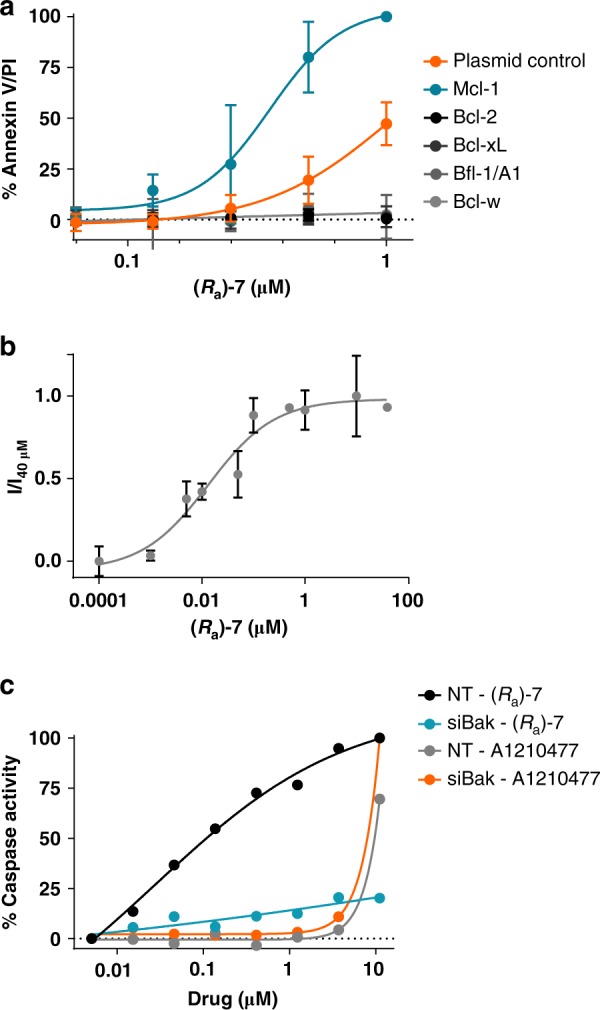
(*R*_a_)-**7** induces on-target intrinsic apoptosis. **a** Apoptosis induction in Eμ-Myc lymphoma cells stably expressing human Mcl-1, Bcl-2, Bcl-xL, Bfl-1/A1, or Bcl-w and treated with (*R*_a_)-**7** for 24 h. Data are shown as mean ± SD (*n* = 3). **b** Isothermal dose–response curve of Mcl-1 at 48 °C plotted against varying concentrations of (*R*_a_)-**7**. I/I_40 μM_: ratio of the signal intensity for each particular sample to the signal intensity obtained with (*R*_a_)-**7** at 40 μM. Data are shown as mean ± SD (*n* = 3). **c** Caspase-3/7 activity in NCI-H23 cells treated with siRNA targeting *Bak* mRNA for 72 h before treatment with (*R*_a_)-**7** for 6 h. Representative data of two independent experiments are shown

We previously showed that (*R*_a_)-**7** has no measurable binding to other Bcl-2 family members in biochemical assays (Supplementary Fig. 2). To assess selectivity in a cellular context, we tested (*R*_a_)-**7** in Eμ-*Myc* lymphoma cell lines stably expressing the prosurvival Bcl-2 proteins Mcl-1, Bcl-2, Bcl-xL, Bfl-1/A1, or Bcl-w. While increased levels of Mcl-1 enhanced the sensitivity to apoptosis induction by (*R*_a_)-**7** compared to control (EC_50_ control = 1.07 μM vs. EC_50_ Mcl-1 = 0.35 μM), the activity of (*R*_a_)-**7** was blocked by overexpression of Bcl-2, Bcl-xL, Bfl-1/A1, or Bcl-w (Fig. 3a and Supplementary Fig. 4a). Similarly, enhanced expression of Bcl-xL in the sensitive cell line NCI-H23 led to resistance to (*R*_a_)-**7**-induced cell death (Supplementary Fig. 4b–c). We confirmed that (*R*_a_)-**7** binds to Mcl-1 in cells using a cellular thermal shift assay (CETSA). This assay is based on the biophysical principle of ligand-induced thermal stabilization of target proteins^36^. Thermal melt profile generated for (*R*_a_)-**7** in cell lysate from MV4-11 cells demonstrated stabilization of Mcl-1 protein in the presence of (*R*_a_)-**7** when compared to untreated cells (Supplementary Fig. 4d). An isothermal dose–response at 48 °C further demonstrated that Mcl-1 protein is stabilized by (*R*_a_)-**7** in a concentration-dependent manner. The CETSA EC_50_ value of 13 nM (95% CI 0.004328–0.03125 nM) (Fig. 3b and Supplementary Fig. 4e) represents the half-maximal concentration for stabilizing Mcl-1 by (*R*_a_)-**7** at 48 °C and is therefore a relative quantification of target engagement in intact cells.

We then investigated whether (*R*_a_)-**7** activity relies on Bak for induction of the mitochondrial apoptotic pathway. We found that depletion of Bak in NCI-H23 cells confers resistance to (*R*_a_)-**7**-induced caspase-3/7 activation and cell death (Fig. 3c and Supplementary Fig. 4f). These findings are in contrast with the minimal and Bak-independent activity seen with the Mcl-1 inhibitor A1210477^37^. Our results demonstrate that (*R*_a_)-**7** promotes apoptosis in cancer cells via on-target activity in a Bak-dependent mechanism.

### Kinetics of apoptosis and cell death induction by (*R*_a_)-7

**Fig. 4:**
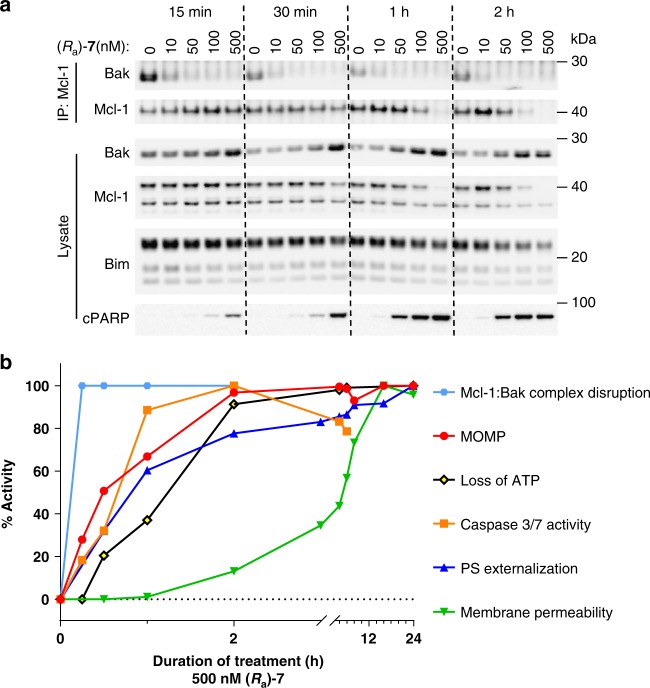
Kinetics of apoptosis and cell death induction by (*R*_a_)-**7**. **a** Whole cell extract (bottom) and IP (top) of Mcl-1 from lysates of MOLP-8 cells treated with (*R*_a_)-**7** at indicated concentrations followed by immunoblot analysis. **b** Summary of the kinetic of (*R*_a_)-**7** effect on Mcl-1:Bak complex disruption, MOMP, cellular loss of ATP, caspase-3/7 activity, phosphatidyl-serine externalization and cell membrane permeability in MOLP-8 cells upon treatment with (*R*_a_)-**7** at 500 nM. Representative data of two independent experiments are shown

To better understand the kinetics of events leading to (*R*_a_)-**7**-induced cell death and given that dissociation of pro-apoptotic Bcl-2 proteins from Mcl-1 initiates mitochondrial apoptosis, we first tested the effect of (*R*_a_)-**7** on Mcl-1 binding to Bak at different time points. MOLP-8 cells that were treated with (*R*_a_)-**7** and endogenous Mcl-1 complexes, were detected by co-immunoprecipitation (co-IP) and immunoblot. Mcl-1 was dissociated from Bak within 15 min even at the low concentration of 10 nM (Fig. 4a). Consistent with the robust activation of caspase-3/7 in this cell line (Table 1), disruption of Mcl-1-Bak complex assembly by (*R*_a_)-**7** was accompanied by increased levels of cleaved PARP. Reduced levels of Mcl-1 protein observed after 1 h of treatment were reversed by treatment with the caspase-3 inhibitor Z-DEVD-FMK suggesting that enhanced caspase activity leads to decreased Mcl-1 protein levels in this sensitive cell line (Supplementary Fig. 5a). Next, we found that AZD5991 reduces the levels of Mcl-1 protein in AZD5991-sensitive but not in AZD5991-resistant MM cell lines further supporting the notion that activation of caspases by AZD5991 reduces Mcl-1 protein levels in AZD5991-sensitive cell lines (Supplementary Fig. 5b).

We next evaluated the effect over time of (*R*_a_)-**7** on markers of apoptosis and cell death in MOLP-8 cells. In less than 2 h of treatment and preceded by Mcl-1 complex disruption, (*R*_a_)-**7** achieved a maximum increase in the mitochondrial outer membrane permeabilization (MOMP), activation of caspase-3/7, and phosphatidyl-serine exposure. These events induced a rapid loss of cellular ATP and maximum increase in cell membrane permeability within 20 h of treatment (Fig. 4b).

Together, our data demonstrate that (*R*_a_)-**7** kills cancer cells by specific and direct inhibition of Mcl-1 that triggers disruption of the Mcl-1-Bak complex and subsequent activation of the Bak-dependent mitochondrial apoptotic pathway. (*R*_a_)-**7** was later nominated as the clinical candidate AZD5991.

### Hematological cells are preferentially killed by AZD5991

**Fig. 5:**
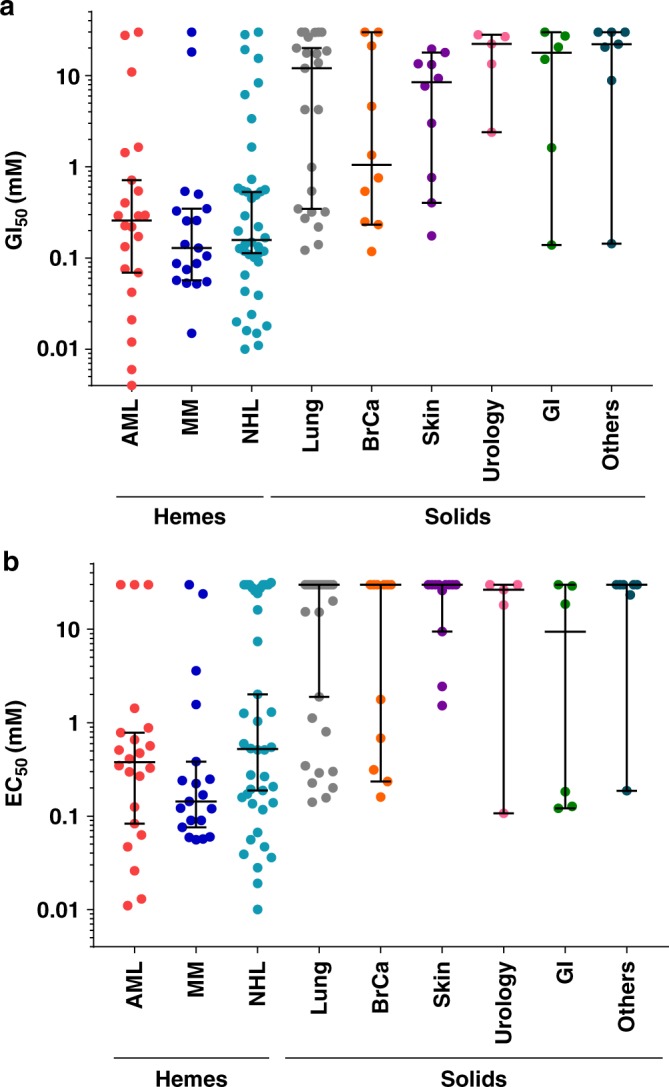
Hematological cell lines are preferentially sensitive to AZD5991. **a** Viability (*n* = 142) and **b** caspase-3/7 induction (*n* = 154) evaluated in cancer-derived cell lines treated with AZD5991 for 24 or 6 h, respectively. Data are shown as median with 95% confidence interval

Next, we investigated the cell killing activity of AZD5991 in a panel of cancer-derived cell lines of hematological or solid tumor origin. As expected for a specific Mcl-1 inhibitor, the cell growth inhibitory activity of AZD5991 correlated closely with the drug’s ability to activate caspase-3/7 in these cell lines (Fig. 5a, b and Supplementary Data 1). Among the cells tested, hematological cell lines were preferentially sensitive to AZD5991. Activity was also seen in subsets of solid tumor cell lines as NSCLC and breast cancer consistent with a previous report^30^.

### AZD5991 exhibits potent anti-tumor efficacy in MM models

**Fig. 6:**
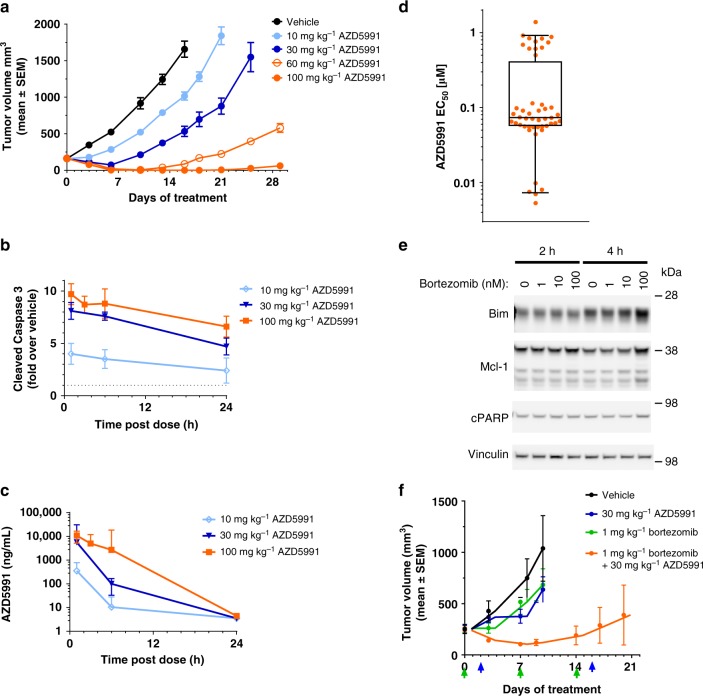
AZD5991 exhibits potent anti-tumor efficacy in MM models. **a** Subcutaneous tumor growth in the MOLP-8 tumor model treated with a single i.v. dose of AZD5991 10–100 mg kg^−1^. Tumor volumes are presented as mean ± SEM, seven mice were evaluated per group. **b** MOLP-8 tumor lysates prepared from mice dosed with a single i.v. dose of AZD5991 at 10, 30, or 100 mg kg^−1^ were evaluated for expression of cleaved caspase-3 by MesoScale discovery assay. Mean ± SD are shown. **c** In vivo plasma concentration of AZD5991 following a single i.v. dose of AZD5991 10–100 mg kg^−1^ to mice with subcutaneous MOLP-8 tumors. AZD5991 plasma concentrations were assessed through the first 24 h following compound administration. Concentrations of AZD5991 (ng  mL^-1^) are plotted on a log_10_ scale as mean ± SD (*n* = 3). **d** Apoptosis in mononuclear cells isolated from bone marrow aspirate of MM patients (*n* = 48) treated with increasing concentrations of AZD5991 for 24 h and evaluated by Annexin V by flow. Each dot represents a unique patient sample. center line indicates the median, bounds of box denote 25% (lower) and 75% (upper) percentile, and whiskers encompass 5–95 percentile. **e** NCI-H929 cells were treated with bortezomib at the indicated concentrations, whole-cell lysates prepared after 2 or 4 h of treatment and protein expression evaluated by immunoblotting. **f** Subcutaneous tumor growth in the NCI-H929 tumor model treated with AZD5991 in combination with bortezomib. Both drugs were dosed intravenously. Arrows indicate day of dosing for AZD5991 (blue) and bortezomib (green). Tumor volumes are presented as mean ± SEM, six mice were evaluated per group

Based on these findings and given that MM cells express high levels of Mcl-1 and its expression level determines clinical outcome^10,38^, we evaluated AZD5991 for its efficacy against growth of MOLP-8 tumors in vivo. A single intravenous (i.v.) dose of AZD5991 led to a dose-dependent antitumor effect ranging from tumor growth inhibition (TGI) to tumor regression (TR) (Fig. 6a). Ten days after treatment, AZD5991 showed 52% and 93% TGI (*p* < 0.0001) at 10 and 30 mg kg^−1^, respectively. At the same time point, AZD5991 at 60 mg kg^−1^ led to 99% TR with no detectable tumors in 6 out of 7 mice, while complete TR was seen in 7 out of 7 mice in the 100 mg kg^−1^ dose group. AZD5991 also showed a dose-dependent duration of response with tumors in the 100 mg kg^−1^ group growing back later than those in the 60 mg kg^−1^ group. The magnitude of in vivo tumor efficacy was correlated with activation of caspase-3 in the tumor (Fig. 6b) and concentration of AZD5991 in plasma (Fig. 6c). Treatment with AZD5991 was well tolerated at all dose levels with no significant body weight loss (Supplementary Fig. 6a), although this should be put in the context of the weaker binding to mouse Mcl-1 (vide supra). In a separate study, we tested if tumors that grow back are still sensitive to AZD5991. A single dose of AZD5991 36 days after the first dose caused tumor regression in 4 out of 4 mice (Supplementary Fig. 6b). In addition, we found that in mice dosed with AZD5991 at 100 mg kg^−1^ on day 0 and day 1, tumors grow back later than those dosed with a single dose of AZD5991 at the same dose level (Supplementary Fig. 6b). These data are consistent with the second dose killing an additional fraction of the small population of cancer cells within the tumor that survived the first dose. Body weight changes were within the acceptable range (Supplementary Fig.6c). We then tested AZD5991 in the MM tumor model, NCI-H929. In line with the data obtained with the MOLP-8 model, a single i.v. dose of AZD5991 at 100 mg kg^−1^ also resulted in complete tumor regression in four out of four mice (Supplementary Fig. 6d).

Next, we evaluated the activity of AZD5991 in primary MM cells. To test this, mononuclear cells isolated from bone marrow of 48 MM patients were treated with AZD5991 for 24 h followed by Annexin V assessment by flow. We found that 71% of MM primary samples display EC_50_ values below 100 nM confirming the high sensitivity to AZD5991 in primary MM bone marrow cells (Fig. 6d).

Together, these findings indicate that AZD5991 kills MM cells in vitro and in vivo and its anticancer activity is driven by activation of apoptosis in the tumor.

We observed that treatment of NCI-H929 cells with the proteasome inhibitor bortezomib enhances the expression of the apoptosis activator Bim in vitro (Fig. 6e). Given that the pro-apoptotic activity of Bim is blocked by association with anti-apoptotic Bcl-2 proteins including Mcl-1, we hypothesized that AZD5991 would enhance the efficacy of bortezomib by displacing Bim from Mcl-1, allowing it to then activate Bak/Bax and promote the mitochondrial apoptotic pathway. To test our hypothesis in vivo, we investigated whether the combination of AZD5991 with bortezomib displays enhanced antitumoral activity compared with single agents alone in the NCI-H929 model grown subcutaneously. Since 100 mg kg^−1^ of AZD5991 drives tumor regression in this model (Supplementary Fig. 6d), we selected a sub-efficacious dose (30 mg kg^−1^) for this combination study. While minimal TGI activity was seen with single agents at the selected dose levels, the combination treatment of AZD5991 and bortezomib induced 88% TR (*p* < 0.05) after 9 days of treatment (Fig. 6f). Together, our data demonstrate that bortezomib primes and sensitizes MM cells to AZD5991 treatment, resulting in enhanced tumor cell killing and prolonged antitumor response. Monotherapy and combination treatments were tolerated based on minimal changes in animal body weight throughout the duration of the study (Supplementary Fig. 6e).

### AZD5991 drives tumor regression in AML models

**Fig. 7:**
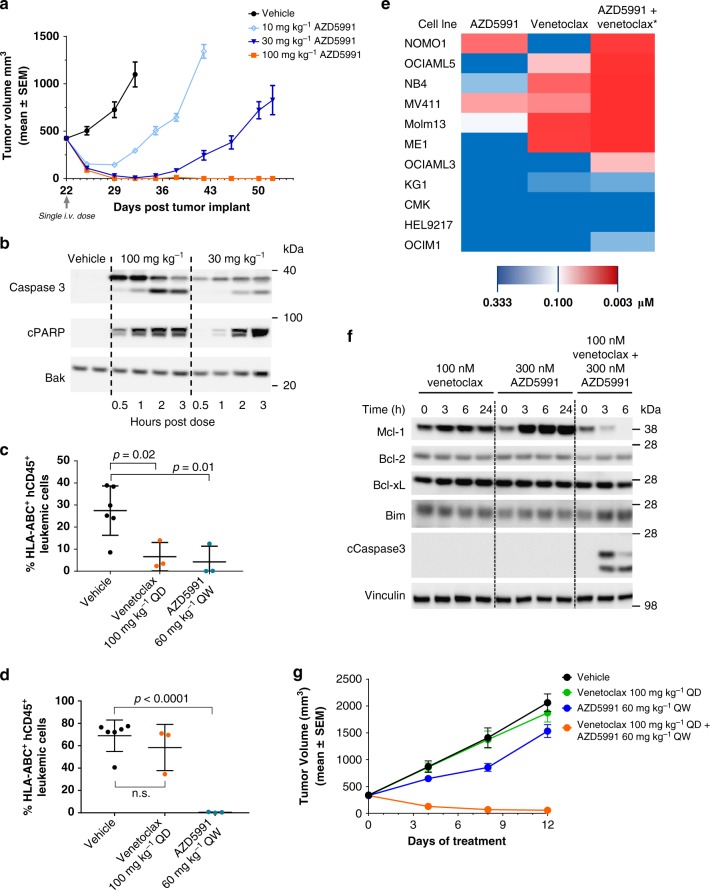
AZD5991 causes tumor regression in AML models. **a** Subcutaneous tumor growth in the MV4-11 tumor model treated with a single i.v. dose of AZD5991 at 10, 30, or 100 mg kg^−1^. Values are presented at mean ± SEM, six mice were tested per group. **b** MV4-11 tumor lysates prepared from mice dosed with a single i.v. dose of AZD5991 at 30 mg kg^−1^ (*n* = 4), 100 mg kg^−1^ (*n* = 4), or vehicle control (*n* = 2) were evaluated for expression of caspase 3 and cleaved PARP by western blotting. Leukemic cells (HLA-ABC^+^ hCD45^+^) were assessed by flow cytometry in peripheral blood (**c**) or bone marrow (**d**) obtained from mice engrafted with MOLM-13 leukemia cells and treated with vehicle (*n* = 6), venetoclax at 100 mg kg^−1^ per oral daily (*n* = 3) or AZD5991 at 100 mg kg^−1^ i.v. once weekly (*n* = 3). Analysis were performed on day 10 after treatment initiation. A non-parametric, unpaired, two-tailed *t*-test was used to calculate significance. **e** Heatmap representing EC_50_ values for caspase activation at 6 h in 11 AML cell lines after treatment with AZD5991 or venetoclax monotherapy or combination. EC_50_ values represented for combination were determined with venetoclax at 160 nM and variable concentrations of AZD5991. **f** OCI-AML3 whole-cell lysates prepared before or after 3, 6, or 24 h of treatment with venetoclax, AZD5991, or combination were evaluated for expression of indicated proteins by immunoblotting. **g** Subcutaneous tumor growth in the OCI-AML3 tumor model treated with AZD5991 (i.v.) in combination with venetoclax (oral) or corresponding single agents. Tumor volumes are presented as mean ± SEM, 10 mice were evaluated per group

Since Mcl-1 is critical for survival and expansion of AML cells^12^, and high sensitivity to AZD5991 was seen in AML-derived cell lines in vitro (Fig. 5a, b), we tested whether AZD5991 has antitumor activity in xenografts derived from the AML cell line MV4-11. A single i.v. dose of AZD5991 exerted dose-dependent anti-tumor activity in this AML model, with doses of 10 and 30 mg kg^−1^ leading to transient TR 7 days after treatment (66% and 93%, *p* < 0.0001), while 100 mg kg^−1^ AZD5991 induced complete TR in six out of six mice within the same dosing period (Fig. 7a). The antitumor efficacy of AZD5991 was directly correlated with a dose-dependent induction of caspase-3 and cleaved PARP (Fig. 7b) demonstrating that AZD5991’s antitumoral activity is caused by induction of apoptosis in the tumor.

Given that AML and MM are bone marrow diseases, we next investigated whether AZD5991 has activity in this compartment by assessing AZD5991’s effect in bone marrow leukemic cells in CIEA-NOG mice engrafted with the AML disseminated model MOLM-13. Mice were dosed with venetoclax at 100 mg kg^−1^ p.o. daily, AZD5991 at 60 mg kg^−1^ i.v. weekly or vehicle control, and analysis performed after 10 days of treatment. We found that both AZD5991 and the specific Bcl-2 inhibitor venetoclax, significantly reduced the percentage of HLA-ABC^+^ hCD45^+^ leukemic cells in peripheral blood compared to vehicle-treated mice (Fig. 7c). Interestingly, AZD5991 but not venetoclax reduced the percentage of leukemic cells in bone marrow in this model (Fig. 7d).

Since the binding affinity of AZD5991 is about 25-fold lower for mouse Mcl-1 vs. human Mcl-1 but only four-fold lower for rat Mcl-1, we investigated the antitumor activity and tolerability of AZD5991 in the rat subcutaneous xenograft model MV4-11. We found that AZD5991 led to a dose-dependent antitumor response in this model with TR observed when dosed at 30 mg kg^−1^ with no significant body weight loss (Supplementary Fig. 7a and b). To further understand whether AZD5991 would be tolerated at efficacious doses, we tested AZD5991 in mice harboring GFP-labeled murine Eµ-Myc lymphoma tumors^39^. AZD5991 dosed at 100 mg kg^−1^ on day 3 and day 10 post-transplantation caused depletion of leukemic cells from peripheral blood on day 11 and prolonged survival compared to vehicle control group (Supplementary Fig. 7c and d). Together, these data indicate that AZD5991 has antitumor efficacy at tolerated doses in models where AZD5991 is expected to have comparable activity in host versus tumor cells.

We then tested whether efficacy of AZD5991 can be enhanced by combination with venetoclax. We tested AZD5991 or venetoclax monotherapy, or combination in a panel of 11 AML cell lines in a caspase activation assay after 6 h of treatment. We found a broad range of sensitivity to single agents with combination benefit observed more frequently in those cell lines that were sensitive to at least one of the drugs when dosed as a single agent (Fig. 7e and Supplementary Table 2). An exception was OCI-AML3 that was inherently resistant to AZD5991 or venetoclax monotherapy, however, combination of these drugs led to potent activation of caspase 3/7. As previously reported in other venetoclax-resistant AML cells^40,41^, treatment with venetoclax caused accumulation of Mcl-1 as soon as 3 h post-treatment (Fig. 7f). We also observed a rapid increase in Mcl-1 levels upon AZD5991 treatment in agreement with our previous observations in AZD5991-resistant cells (Supplementary Fig. 5c). Co-treatment with venetoclax plus AZD5991 caused rapid activation of caspase 3 and sharp reduction in the levels of Mcl-1 with no changes in protein expression observed for other Bcl-2 proteins.

We then evaluated the antitumor activity of AZD5991/venetoclax combination in the OCI-AML3 subcutaneous mouse xenograft model. Consistent with the in vitro data, we found minimal growth inhibition activity with monotherapies while TR were seen in eight out of eight mice in the combination arm (Fig. 7g). Changes in body weight were within the acceptable range (Supplementary Fig. 7e).

Together, these data suggest that combination of AZD5991 with the selective Bcl-2 inhibitor, venetoclax, could be effective at overcoming resistance associated with their monotherapy treatments in AML.

## Discussion

The evasion of apoptosis is one of the hallmarks of cancer^1^, highlighting the important role of this pathway in tumorigenesis. It follows that therapies aimed at reversing or preventing this alteration might be effective as anti-cancer agents. This has been recently confirmed by the success seen in the clinic with the Bcl-2 antagonist venetoclax^42,43^.

Mcl-1 has attracted attention because of its established role in preventing cell death by binding and sequestering pro-apoptotic BH3 proteins, thereby mediating cancer cell survival, as well as intrinsic and acquired resistance against cytotoxic and some targeted therapies, including venetoclax^17,18,44–47^. Here we report discovery, mechanism of action and cellular and in vivo activity of AZD5991, a first generation Mcl-1 inhibitor that is currently in a phase I clinical trial for evaluation in patients with hematological malignancies^24^.

Application of structure-based drug design to a new binding mode of a known inhibitor scaffold bound to Mcl-1 led to identification of an induced-fit pocket in Mcl-1. This protein movement, along with the conformation of the crystallized inhibitor allowed us to design and synthesize a series of macrocyclic inhibitors of Mcl-1, ultimately leading to the identification of (*R*_a_)-**7**, now the clinical development candidate, AZD5991. The rigid macrocyclic conformation of AZD5991 resulted in rapid on-rate of binding to Mcl-1 (SPR *k*_on_ = 3.8 × 10^6^ M^−1^ s^−1^), while optimized interactions with Mcl-1 resulted in slow off-rate (SPR residence time = 30 min) and high binding affinity. The specific nature of the binding interactions with and unique flexibility of Mcl-1 led to high selectivity of binding over other anti-apoptotic Bcl-2 family proteins (Bcl-2, Bcl-xL, Bcl-w, and Bfl-1).

Our mechanistic studies demonstrated that AZD5991 binds directly to Mcl-1 in cells, as evidenced by CETSA and co-IPassays, releasing Bak from the Mcl-1:Bak complex within 15 min of treatment. This event rapidly initiates the mitochondrial apoptotic cascade by increasing MOMP, caspase activation and externalization of phosphatidyl-serine that ultimately leads to loss of ATP and disruption of the cell membrane in less than 24 h. The cytotoxic activity of AZD5991 observed in hematological as well as solid tumor cell lines tightly correlated with activation of markers of apoptosis. Previous studies have shown that chemotherapy-induced apoptosis promotes cleavage of Mcl-1 by caspases and enhances Mcl-1 protein turnover in various cancer cell lines^5,6^. In agreement with these reports, we found that apoptosis induction by AZD5991 reduces Mcl-1 protein levels by a mechanism dependent on caspase activity. This effect was seen in sensitive but not resistant cell lines further supporting on-target mechanism of cytotoxicity by AZD5991 in these cell lines.

In vivo, AZD5991 exhibits potent anti-tumor activity with complete (100%) TR in several MM and AML mouse and rat xenograft models after a single tolerated intravenous injection. In these in vivo studies, the cytotoxic activity of AZD5991 tightly correlated with induction of the mitochondrial apoptotic pathway as evidenced by cleavage of caspase-3 and PARP. In addition, AZD5991 was more efficacious than venetoclax clearing cancer cells from bone marrow as determined in a disseminated model of AML. These data are consistent with previous findings that showed dependency on Mcl-1, but not on the pro-survival Bcl-2 proteins Bcl-xL, Bcl-2 or Bcl-w, in a mouse model of AML^12^, and that inhibition of Mcl-1 by pharmacological, gene editing or peptide-based approaches kills MM^30,48^ and AML^30^ cells in vitro and in vivo. In addition, AZD5991 shows enhanced efficacy against growth of MM and AML subcutaneous tumors when combined with agents that enhance Bim activity and/or Mcl-1-dependency, such as bortezomib or venetoclax.

In conclusion, we have discovered AZD5991, a potent and direct inhibitor of Mcl-1 with high selectivity versus other Bcl-2 family proteins. AZD5991 displays the hallmarks of a true Mcl-1 inhibitor: (i) sub-nanomolar-binding affinity; (ii) target engagement in cells; (iii) cytotoxic activity in Mcl1-dependent but not Mcl-1-independent cell lines; and (iv) rescue of cell killing by depletion of Bak. The remarkable cytotoxic activity of AZD5991 in in vitro and in vivo models of MM and AML at tolerated doses supported selection of AZD5991 as a clinical candidate for the treatment of patients with hematological malignancies.

We anticipate that AZD5991 will serve as a useful probe not only to gain knowledge on the design of protein–protein interaction inhibitors but also further understand Mcl-1 biology. If the safety and efficacy for AZD5991 are confirmed in the clinic, AZD5991 may provide a therapeutic option for patients with hematological malignancies and solid cancers that rely on Mcl-1 for survival, and serve as an ideal partner for combination therapies designed to overcome and even prevent the emergence of resistant clones.

## Methods

### Chemistry

Synthetic schemes, detailed procedures, and characterization of compounds **3** and **4** (Supplementary Figure 14), **5** and **6** (Supplementary Figure 15), and (*R*_a_)-**7** and (*S*_a_)-**7** (Supplementary Figure 16) can be found in the Supplementary Information as well proton assignment and nOe data for (*R*_a_)-**7** (Supplementary Tables 3 and 4).

### Crystallography

Crystallization conditions for compounds **1**, **4** and **7** in complex with mouse–human chimeric Mcl-1 can be found in the Supplementary Information, including a summary of structural and refinement statistics (Supplementary Table 5). The crystal structures have been deposited to the RCSB protein data bank. The crystal structure of **1** bound to Mcl-1 (pdb id: 6FS2) was obtained as a gallery structure from Proteros (http://www.proteros.de).

### Labeled expression and purification of Mcl-1

The 6His-TEV-Mcl-1(162–326) construct in a pET vector was expressed in BL21-GOLD cells and inducted by IPTG. ^15^N-labeled GS-Mcl-1 was obtained by growing cells in Isogro-15N (Sigma-Aldrich) media (5 g L^-1^) containing 50 µg mL^-1^ Kanamycin and 12.5 µg mL^-1^ Tetracycline. Expression was induced at OD^600^ = 0.8 with 0.1 mM IPTG. Cells were then incubated overnight at 30 °C, harvested by centrifugation and lysed by sonication in buffer H (40 mM Hepes pH 8.0, 300 mM NaCl, 10 mM imidazole, 1 mM TCEP), supplemented with 0.3 mg mL^-1^ lysozyme, 2.5 U mL^-1^ benzonase, and EDTA-free protease inhibitors (Roche). The sample was centrifuged and supernatant collected and incubated with Ni-NTA resin (60 min at 4 °C). Next, resin was separated from the flow-through and washed with buffer H. Bound protein was eluted with buffer H containing 200 mM imidazole. The His-tag was cleaved from the Mcl-1 sequence by the addition of TEV protease with the mixture left at 4 °C dialyzing overnight into buffer H. The sample was then re-loaded onto a re-equilibrated Ni-NTA resin to separate cleaved His-tag, un-cleaved protein and TEV protease from cleaved Mcl-1 protein. After His-tag removal, the protein construct contained two extra residues (Gly-Ser) before the start of the native Mcl-1 sequence. The Mcl-1 sample was then loaded onto a Superdex 75 size-exclusion column equilibrated with gel-filtration buffer (50 mM HEPES, pH 7.4, 50 mM NaCl, 1 mM TCEP, 0.1 mM EDTA, 0.02% NaN_3_). The pooled fractions from the purified Mcl-1 were concentrated using an Amicon stirred cell using 10 kDa molecular weight cut-off membrane.

### NMR-binding studies of Mcl-1

NMR spectra were recorded at 298 K on a Bruker Avance 600 MHz spectrometer running Topspin 2.3 equipped with a 5 mm TCI Cryoprobe with *Z*-axis gradients. The Mcl-1 sample comprised 80 µM ^15^N-labeled protein in 500 µL of 50 mM Tris pH 7.4, 50 mM NaCl, 1 mM TCEP, 0.1 mM EDTA, 0.02% NaN_3_, and 5% D_2_O. Binding was detected by ^1^H–^15^N 2D Transverse relaxation optimized spectroscopy (TROSY) spectra of the protein (F2 × F1) 2048 × 50 complex pairs (in Echo-Antiecho mode), 12019 × 1800 Hz sweep width, 85.2 ms × 27.8 ms acquisition times. Compound titration was recorded from fresh compound stock in DMSO-d6, and spectra were recorded at ligand concentrations of 0.04, 0.08, 0.16, 0.32, 0.63, and 1.24 mM. The affinity of the compounds was determined via simultaneous nonlinear fitting of chemical shift perturbations, of four residues, that showed fast exchange shifts, versus compound concentration (total compound added minus 80 µM which is required to create the 1:1 complex) using the law of mass action.

### Mcl-1 binding by SPR

A Biacore T200 instrument (GE Healthcare) was used to monitor binding interactions using a direct binding assay format. 6His-tagged Mcl-1 protein (E171-G327) was immobilized using NTA capture-coupling at a flow rate of 10 μL min^-1^ and using an immobilization running buffer containing 10 mM HEPES, 300 mM NaCl, 1 mM TCEP, and 0.05% Tween-20 at 25 °C. Briefly, the sensor surface was activated with a 1 min injection of 0.5 mM NiCl_2_ and a 7 min injection of a mixture of 11.5 mg mL^-1^ *N*-hydroxysuccinimide with 75 mg mL^-1^ 1-ethyl-3-(3-dimethylaminopropyl)carbodiimide hydrochloride. Approximately 300 response units of Mcl-1 protein (2 μg mL^-1^ in immobilization running buffer) were immobilized using the ‘aim for’ function in the T200 Control Software (GE Healthcare). Remaining reactive esters were blocked using a 7 min injection of 1 M ethanolamine. Reference flow cells were prepared without protein. All binding measurements were performed in 10 mM Tris, pH 7.5, 300 mM NaCl, 1 mM TCEP, 1% DMSO, and 0.02% Tween-20 at 25 °C at a flow rate of 30 μL min^-1^. Buffer was primed through the instrument overnight to stabilize the surface before subsequent assay steps. Prior to kinetic analysis, solvent calibration and double referencing subtractions were made to eliminate bulk refractive index changes, injection noise, and data drift. Affinity and binding kinetic parameters were determined by global fitting to a 1:1-binding model within the Biacore T200 Evaluation Software (GE Healthcare). SPR sensograms are shown in Supplementary Fig. 3.

### TR-FRET-binding assays and *K*_i_ calculation

*N*-terminal GST-tagged-Mcl-1 protein from Mcl-1 (E171-G327), *N*-terminal GST-tagged Bcl-xL protein from Bcl-xL (1-209), N-Terminal 6His-tagged Bfl-1 protein from Bfl-1 (M1-K151), N-Terminal 6His-tagged Bcl-w protein from Bcl-w (M1-R171), and C-Terminal 6His-tagged Bcl-2 protein from Bcl-2 (M1-F212) were expressed as a tagged fusion protein in *E. coli* and subsequently purified via Glutathione Sepharose-affinity or Ni-NTA resin purification, and size-exclusion chromatography.

For TR-FRET, tagged fusion proteins were incubated with a Europium-labeled antibody and a HyLite Fluor 647-labeled peptide, or a biotin-labeled peptide with a streptavidin-labeled ulight fluorophore allowing the assembly of donor and acceptor dye pairs for use in protein-binding assays.

The assay was performed in 384-well plates and IC_50_ values were assessed from a 10-point, half-log_10_ dilution starting at 100 or 10 µM of compound. The reaction final concentrations for Mcl-1 were 1.5 nM GST-Mcl-1, 0.5 nM LanthaScreen Eu tagged GST antibody (LifeTechnologies cat#PV5594), and 4 nM HyLite Fluor 647-labeled Bim peptide C (Hilyte647 C2 Maleimide)-WIAQELRRIGDEFN; for Bcl-xl were 2 nM GST-Bcl-xl, 2 nM LanthaScreen Eu-tagged GST antibody, and 10 nM HyLite Fluor 647-labeled BAK peptide C(Hilyte647 C2 Maleimide)-GGGQVGRQLAIIGDDINR; for Bfl-1 were 4 nM His-Bfl-1, 0.5 nM LanthaScreen Eu-tagged HIS antibody (PerkinElmer cat#AD0205), and 10 nM HyLite Fluor 647-labeled BIM peptide C(Hilyte647-C2-Maleimide)-WIAQELRRIGDEFN; for Bcl-2 and Bcl-w: 2 nM His-Bcl-2, or 2 nM His-Bcl-w, with 0.5 nM LanthaScreen Eu-tagged HIS antibody, 10 nM biotin-labeled Bim peptide (Biotin-lc-GGMRPEIWIANELRRIGDEFNA), and 2.4 nM a streptavidin labeled ULight Fluorophore (PerkinElmer cat#TRF0102-D). Reactions were incubated at 24 °C for 120–180 min before reading on a Tecan (InfiniteM1000 spectrofluorometer) with excitation at 340 nm and emission at 612 and 665 nm. Ratio of fluorescent emission intensity at 665–620 nm was calculated for each reaction (equation 1). Percent inhibition was calculated based upon min (control compound) vs. max (DMSO) according to Eq. (2) and the IC_50_ was derived the smart fit curve (Genedata screener) of % inhibition vs. concentration. *K*_i_ values were calculated from Eq. (3) and the parameters in Table 2.

1 $${\mathrm {Test}}\,{\mathrm{ratio = Emission}}\,{\mathrm{665}}\,{\mathrm{nm/Emission}}\,{\mathrm{612}}\,{\mathrm{nm}} \ast {\mathrm{10,000}}$$

2 $${{\%}{\mathrm{inhibition}} = 100 - [ ({\rm{Test}}\,{\rm{ratio}}-{\mathrm{Min}}\,({\mathrm{compound}}\,{\mathrm {control}} ) )/} \\ { ( {\mathrm{Max}}\,( {\mathrm{DMSO}}\,{\mathrm{control}} ) - {\mathrm{Min}}\,( {\mathrm {compound}}\,{\mathrm{control}} ) ) ]}$$

3 $$K_{\mathrm {i}} = \frac{{{\mathrm {IC}}_{50}}}{{\frac{L}{{K_{\mathrm {d}}}} + 1}}$$

**Table 2:** TR-FRET assay-binding parameters of Bim or Bak peptides to Bcl-2 pro-survival proteins used in *K*_i_ calculations

		Peptide, *K*_d_ (nM)	Peptide conc. (nM)
Mcl-1	Hylite Bim peptide	1.5	4
Bcl-xL	Hylite Bak peptide	10	10
Bcl-2	Biotin Bim peptide(biotin-Ic-)	5	10
Bcl-w	Biotin Bim peptide(biotin-Ic-)	10	10
Bfl-1	Hylite Bim peptide	10	10

### Cell lines used in this study

Cells used in these studies tested negative for mycoplasma contamination and were authenticated by short tandem repeat profile. See full list of cell lines and origin in Table 3.

**Table 3:** List and origin of cell lines used in these studies

Cell Line	Source	Cell line	Source	Cell line	Source	Cell line	Source
5637	ATCC	JVM2	DSMZ	NCI-H146	ATCC	RCK8	DSMZ
647V	DSMZ	Karpas422	DSMZ	NCI-H1568	ATCC	REC1	ATCC
A101D	ATCC	Karpas620	DSMZ	NCI-H1703	ATCC	RI1	DSMZ
A2058	ATCC	Kasumi1	ATCC	NCI-H1734	ATCC	RPCIWM1	RPCI
A253	ATCC	Kasumi3	ATCC	NCI-H196	ATCC	RPMI8226	ATCC
A375	ATCC	KG1	ATCC	NCI-H1975	ATCC	RT4	ATCC
AMO1	DSMZ	KG1a	ATCC	NCI-H209	ATCC	SBC5	JCRB
ARH77	ATCC	KMS11	JCRB	NCI-H2110	ATCC	SKBR3	ATCC
Bjab	DSMZ	KMS12BM	DSMZ	NCI-H2122	ATCC	SKLU1	ATCC
BT20	ATCC	KMS12PE	DSMZ	NCI-H2126	ATCC	SKMEL2	ATCC
BT549	ATCC	KMS26	JCRB	NCI-H2171	ATCC	SKMEL24	ATCC
Calu1	ATCC	KMS34	JCRB	NCI-H2286	ATCC	SKMEL3	ATCC
CMK	DSMZ	L363	DSMZ	NCI-H23	ATCC	SKOV3	ATCC
Colo205	ATCC	LK2	JCRB	NCI-H322	ATCC	SNU1197	KCLB
COLO829	ATCC	LnCAP	ATCC	NCI-H345	ATCC	SNU16	ATCC
Daudi	ATCC	LP1	DSMZ	NCI-H358	ATCC	Sudhl10	DSMZ
DLD1	ATCC	LUDLU1	ECACC	NCI-H446	DSMZ	Sudhl16	ATCC
DMS114	ATCC	MALME3M	ATCC	NCI-H460	ATCC	Sudhl2	ATCC
DMS53	ATCC	MAVER1	ATCC	NCI-H526	ATCC	Sudhl4	DSMZ
DMS79	ATCC	MCF7	DSMZ	NCI-H647	ATCC	Sudhl5	DSMZ
DOHH2	DSMZ	MDAMB231	ATCC	NCI-H82	ATCC	Sudhl6	DSMZ
DU145	DSMZ	MDAMB468	ATCC	Nomo1	DSMZ	Sudhl8	DSMZ
EJM	DSMZ	ME1	DSMZ	OCIAML2	DSMZ	T47D	ATCC
EOL1	DSMZ	MEWO	ATCC	OCIAML3	DSMZ	THP-1	ATCC
EVSAT	DSMZ	MINO	ATCC	OCIAML5	DSMZ	TMD8	GmbH
FaDu	ATCC	ML2	DSMZ	OCILY1	DSMZ	Toledo	ATCC
G361	ATCC	MM1R	NU	OCILY10	NHI/NCI	U266B1	ATCC
GIST 430/654	DFCI	MM1S	NU	OCILY19	DSMZ	U2932	DSMZ
GIST T1	CB USA	MOLM13	DSMZ	OCILY3	NIH/NCI	U2OS	ATCC
GRANTA519	DSMZ	MOLP8	DSMZ	OCILY7	DSMZ	ULA	DSMZ
HBL1	ATCC	MONOMAC6	DSMZ	OCIM1	DSMZ	VAL	DSMZ
HCC1187	ATCC	MUTZ3	DSMZ	OE21	ECACC	Will2	DSMZ
HCC1954	ATCC	MV411	ATCC	OPM2	DSMZ	WM2664	ATCC
HCC827	ATCC	MWCL1	MC	OV90	ATCC	WSUDLCL2	DSMZ
HEL92.1.7	JCRB	Namalwa	DSMZ	OVCAR3	ATCC	WSUNHL	DSMZ
HL60	ATCC	NB4	DSMZ	Pfeiffer	ATCC	Z-138	ATCC
HMCB	ATCC	NCI-H929	ATCC	PL21	DSMZ	ZR751	ATCC
JEKO1	ATCC	NCI-H1048	ATCC	Raji	ATCC		
JJN3	DSMZ	NCI-H1395	ATCC	Ramos	ATCC		

*ATCC* American Tissue Culture Collection, *KCLB* Korean Cell Line Bank, *JCRB* Japanese Collection of Research Bioresources Cell Bank, *DSMZ* German Collection of Microorganisms and Cell Cultures, *NU* Northwestern University, *NIH/NCI* Dr. Staudt at NIH/NCI, *GmbH* Dr. Krappman at GmbH, *MC* Mayo Clinic, *DFCI* Dr. Fletcher at DFCI, *CB USA* CosmoBio USA, *RPCI* Roswell Park Cancer Institute, *ECACC* The European Collection of Cell Cultures

### Caspase activation and cell viability assay

Cells were plated at 3000 cells/well in 384-well white plates in corresponding cell growth media. Cells were treated with compounds for 2 or 6 h for caspase-3/7 activation assays or 24 h for cell viability assays with a final DMSO concentration of 0.3%. Caspase-3/7 activation was subsequently determined using a Caspase-Glo 3/7 Reagent (Promega) and viability was assayed using the CellTiter-Glo Reagent (Promega) as described in manufacturer’s instructions. Dose–response curves for caspase-3/7 activation and viability were plotted and analyzed (including EC_50_ and GI_50_ determination) using GraphPad Prism. Percentage of caspase activation was calculated against the maximum caspase activation value (100%) obtained with a proprietary cell kill control. Results from the cell viability assays were normalized to the samples without treatment at time 0.

### CETSA

Melt curves for Mcl-1 were determined by incubating MV4-11 cells with (*R*_a_)-**7** at 40 μM final concentration or DMSO at 1% (control) for 15 min at 37 °C. Cell viability was measured via Trypan Blue exclusion before and after compound incubation. Cells were washed once, resuspended in PBS, divided into aliquots and subjected to a 11-step heat challenge between 37 and 62 °C for 3 min. After a 1 min cool down period, NP-40 was added to a final concentration of 0.4% followed by immediate cell lysis via three rounds of freeze–thawing using liquid N_2_. Samples were centrifugated and an aliquot of the supernatant was analyzed by western blotting as described below.

Isothermal dose–response (ITDR) curves were determined with MV4-11 cells treated as above. Cell suspension was divided into aliquots and (*R*_a_)-**7** was added. (*R*_a_)-**7** was tested at concentrations ranging from 40 μM to 100 pM with DMSO at 1% as a control. Cells were incubated with ligand at 37 °C for 15 min. Aliquots were heated for 3 min at 48 °C, as determined previously from the Mcl-1 melt curve, and lysed as described above. Samples were centrifugated and an aliquot of the supernatant was analyzed by western blotting as described below. Band intensities were quantified using a GE ImageQuant LAS-4000. Luminescence counts were normalized against control at 37 °C for the melt curve, and the ITDR data was normalized against vinculin and then compared as a ratio to the maximum compound concentration. Dose–response curves were fitted using the sigmoidal dose response algorithm in GraphPad Prism. The obtained CETSA EC_50_ values represent the half-maximal concentration of the ligands for stabilizing Mcl-1 at 48 °C. The quoted EC_50_ value with 95% confidence interval is therefore a relative measure of target engagement of compound available for binding to Mcl-1 in MV4-11 cells.

### Generation of Bak-knockdown NCI-H23 cells

NCI-H23 cells were trypsinized and resuspended in growth medium. SiRNA targeting Bak (cat#S1881, ThermoFisher Scientific) or non-targeting siRNA (cat#4390847, ThermoFisher Scientific), and Lipofectamine RNAiMAX (ThermoFisher Scientific) were mixed in OPTIMEM and incubated at room temperature for 20 min. Next, cell suspension was added to each siRNA master mix, dispensed into each well of a 96-well plate and incubated for 72 h. Following this incubation, (*R*_a_)-**7** was added at various concentrations to transfected NCI-H23 cells with a final DMSO concentration of 0.3% and samples taken to assess Bak protein knockdown by western blotting. Caspase-3/7 activation was measured 6 h after (*R*_a_)-**7** addition using the Caspase-Glo 3/7 Assay (Promega) as described above.

### Generation of Eμ-Myc stable cell lines

The retroviral plasmids to express human Bcl-2 family members in murine Eμ-*Myc* lymphoma cells are MSCV-hMCL-1-GFP, MSCV-hBCL-2-GFP, MSCV-hBCL-XL-GFP, MSCV-hBFL-1/A1-GFP, and MSCV-hBCL-w-GFP. To produce viruses, constructs of interest were co-transfected into HEK293T cells with the plasmid encoding the packaging viruses (p-CL-AMPHO provided by Phillip Darcy from Peter MacCallum Cancer Center, Melbourne, Australia) using standard calcium phosphate transient transfection technique. The retroviruses were harvested, filtered and used to infect target cells following standard procedures. Following 72 h incubation, cells were FACS-sorted for fluorescent protein positive expression and returned to exponential growth culture for in vitro interrogation.

### Generation of doxycycline-inducible Bcl-xL stable cell line

The lentiviral plasmid pTRIPZ-FLAG-BCL-XL was used for generation of NCI-H23 cells stably expressing doxycyline-inducible Bcl-xL. To produce viruses, the construct of interest was co-transfected into HEK293T cells with a plasmid encoding the packaging viruses (pPACKH1 Lentivector Packaging Kit, cat#LV500A1 SystemBioscience) using Lipofectamine transfection reagent (ThermoFisher Scientific). The lentiviruses were harvested, filtered, and used to infect target cells following standard procedures. Transduced cells were then subject to puromycin selection for 3 weeks before evaluation in apoptosis and cell proliferation assays.

### Apoptosis assays by flow cytometry

Cells treated with (*R*_a_)-**7** or DMSO control were centrifuged, washed once with PBS and stained with tetramethylrhodamine ethyl ester (TMRE) (cat#564696, BDBioscience) for 20 min at 37 °C, 7-amino-actinomycin D (7-AAD) (cat#51-68981E, BDPharmigen) or Annexin V-FITC (cat#556419, BDPharmigen) and propidium iodide (cat#P3566, Invitrogen) for 15 min in the dark at room temperature. Samples were then centrifuged, washed twice with PBS and analyzed by flow cytometry in a BD LSRFortessa. FACSDiva software was used for data collection and FlowJo software for data analysis. Gating strategies are shown in Supplementary Figure 8.

Data presented in Fig. 4b was calculated as follows:

4 $${{\mathrm {TMRE}}:( {\mathrm {TMRE}}\,{\mathrm {negative}}\,{\mathrm {sample}} - {\mathrm {TMRE}}\,{\mathrm {negative}}\,{\mathrm {DMSO}})} \\ {/ ( {\mathrm{TMRE}}\,{\mathrm {negative}} \,500 \,{\mathrm {nM}}\,{\mathrm{AZD5991@24h}} - {\mathrm {TMRE}}\,{\mathrm {negative}}\,{\mathrm {DMSO}} ) \times 100}$$

5 $${\mathrm{Annexin\hskip 2pt V:( Annexin\hskip 2pt V\hskip 2pt positive\hskip 2pt sample ) - Annexin\hskip 2pt positive\hskip 2pt DMSO{/}}}\\ {\mathrm{( Annexin\hskip 2pt positive\hskip 4pt 500nM\hskip 2pt AZD5991@24h ) - Annexin\hskip 2pt positive\hskip 2pt DMSO \times 100}}$$

6 $${\mathrm{7AAD:( {7AAD\hskip 2pt positive\hskip 2pt sample} ) - 7AAD\hskip 2pt positive\hskip 4pt 500nM\hskip 2pt AZD5991@0.5h/}}\\ {\mathrm{( {7AAD\hskip 2pt positive\hskip 4pt 500nM\hskip 2pt AZD5991@16h} ) - 7AAD\hskip 2pt positive\hskip 2pt 500nM\hskip 2pt AZD5991@0.5h \times 100}}$$

### Immunodetection of cleaved Caspase-3

Tumors were lysed in lysis buffer (25 mM Tris–HCl pH 7.4, 130 mM NaCl, 2.7 mM KCl, 1% NP-40, protease, and phosphatase inhibitors cocktails (Roche)) and protein concentration was measured as mentioned above. Lysates were prepared for immunodetection of cleaved Caspase-3 by using the Cleaved (Asp175)/total Caspase-3 Assay Kit (cat#K151CFD, MesoScale) according to manufacturer’s instructions and analyzed using Sector Image 2400.

### IP

MOLP-8 cells were treated with (*R*_a_)-**7** or DMSO control for 30 min. Then samples were centrifuged and pellet resuspended in ice-cold lysis buffer (10 mM HEPES pH 7.0, 150 mM NaCl, 1% CHAPS, 1 mM EDTA, 5 mM MgCl_2_, protease and phosphatase inhibitors cocktails (Roche)) and incubated for 20 min on ice with vortexing every 5 min. Next, samples were centrifuged and protein concentration assessed as mentioned above. Samples were pre-cleared for 30 min using rotation at 4 °C with 50% slurry of Protein A/G magnetic beads (ThermoFisher Scientific) followed by incubation with anti-Mcl-1 antibody (cat#MABC43, EMD Millipore) overnight at 4 °C with rotation. Protein A/G magnetic beads were then added for 1 h at 4 °C with rotation. Beads were washed four times with lysis buffer / PBS (1:1), then 10% sample reducing agent (LifeTechnologies) was added to each IP pellet followed by western blotting analysis.

### Western blotting

Lysate or IP samples were heated at 100 °C for 10 min and run on Bis–Tris NuPAGE in MES buffer. Protein was transferred onto nitrocellulose membranes using the Invitrogen iBlot (LifeTechnologies) system. Blots were blocked with 5% milk in Tris-buffered saline with 0.05% Tween (TBST) for 1 h at room temperature. Blots were incubated with rocking at 4 °C overnight with the following primary antibodies in TBST with milk at 5%: Mcl-1 (cat#sc-819, Santa Cruz Biotechnology), Bak (cat#1542-1, Epitomics or cat#ab32371, Abcam), Bax (cat#2772, Cell Signaling Technology), Cleaved-Parp (cat#9541, Cell Signaling Technology), Cleaved-Caspase-3 (cat#9661, Cell Signaling Technology), Caspase-3 (cat#9662, Cell Signaling Technology), Noxa (cat#1036S, Epitomics or cat#ab13654, Abcam), Bim (cat#ab32158, Abcam), Bcl-2 (cat#1071-1, Epitomics or cat#ab32124, Abcam), Bcl-xL (cat#54H6 or cat#2764, Cell Signaling Technology), Puma (cat#sc-19187, Santa Cruz Biotechnology), and vinculin (cat#V9131, Sigma-Aldrich; cat#4650, Cell Signaling Technology). After incubation, blots were washed three times with TBST. For Mcl-1, blots were incubated with a 1:200 dilution of Clean-Blot IP Detection Reagent (cat#21230, ThermoScientific) for 1 h at room temperature. For all others, blots were incubated with a 1:4000 dilution of secondary antibody (HRP-conjugated cat#A0545, Sigma; HRP-conjugated goat anti-rabbit IgG, Fc fragment cat#111-035-046, Jackson ImmunoResearch or HRP-conjugated horse anti-mouse IgG cat#7076, Cell Signaling Technology) for 1 h at room temperature. Blots were washed four times in TBST. SuperSignal WestDura (ThermoScientific) or Immobilon Western Chemiluminescent HRP Substrate (cat#WBKLS0500, EMD Millipore) was used as the HRP substrate. Bands were detected using a GE ImageQuant LAS 4000. All uncropped blots are shown in Supplementary Figures 9–13.

### Ex vivo studies in primary MM bone marrow samples

All samples were collected following relevant ethical regulations according to the Emory University Institutional Review Board-approved study protocol. Informed consent was obtained from all patient participants. Bone marrow aspirates from consenting myeloma patients were diluted to 25 mL with PBS, filtered and underlaid with lymphocyte separation medium (cat#25-072-CV, Mediatech). The buffy coat was collected, washed with PBS, resuspended in RPMI-1640 supplemented with 10% heat- inactivated FBS, 100 U  mL^-1^ penicillin, 100 g mL^-1^ streptomycin, and 2 mM l-glutamine at a concentration of 2.5 × 10^5^ cells mL^-1^ and incubated with various concentrations of AZD5991 for 24 h. Apoptosis was measured by flow cytometry after staining with anti-CD38-PE, anti-CD45-APC-Cy7, and Annexin-V-FITC. In samples from patients recently treated with daratumamab, cells were stained with multi-epitope anti-CD38-FITC, anti-CD45-APC-Cy7, and Annexin-V-PacificBlue. Data were acquired on a BD FACSCantoII cytometer and analyzed using FACSDiva software. Gating strategy is shown in Supplementary Figure 8. EC_50_ values were determined by linear regression analysis of the dose–response curves.

### Efficacy studies in mice and rat xenograft models

Female C.B.-17 SCID mice and female nude rats were purchased from Charles River Laboratories. Mice were 5–8 weeks old and rats were 7 weeks old at the time of tumor implantation. All xenograft studies were conducted at our Association for the Assessment and Accreditation of Laboratory Animal Care accredited facility in Waltham, MA in accordance with ethical regulations described in the guidelines established by the internal Institutional Animal Care and Use Committee. AZD5991 was formulated in 30% 2-Hydroxypropyl-beta-cyclodextrin (HPBCD) at pH 9, bortezomib was formulated in saline solution (0.9% NaCl) and venetoclax in 10% ethanol, 30% polyethanolglycol (PEG) 400, 60% Phosal PG50. In mice, drugs were dosed intravenously in a volume of 5 mL kg^-1^ except for venetoclax that was dosed orally in a volume of 10 mL kg^-1^. One million MV4-11, five million MOLP-8, ten million NCI-H929 or five million OCI-AML3 cells were injected subcutaneously in the right flank of mice in a volume of 0.1 mL. In rats, AZD5991 was dosed intravenously in a volume of 10 mL kg^-1^. Ten million MV4-11 cells were injected subcutaneously in the right flank of rats in a volume of 0.1 mL. Tumor volumes (measured by caliper), animal body weight, and tumor condition were recorded twice weekly for the duration of the study. The tumor volume was calculated using the formula: length (mm) × width (mm)^2^/0.52. For efficacy studies, growth inhibition from the start of treatment was assessed by comparison of the differences in tumor volume between control and treated groups. Statistical significance was evaluated using non-parametric, unpaired, two-tailed *t*-test. For efficacy studies, animals were randomized based on tumor volumes using stratified sampling, and enrolled into control and treatment groups. Dosing began when mean tumor size reached ~230 mm^3^ in mice and 900 mm^3^ in rats. Mice were euthanized once tumor size reached 2000 mm^3^. Mice were excluded from the study upon body weight loss > 10% of the body weight at the initiation of the study. For pharmacokinetic analysis, plasma samples were precipitated and diluted with acetonitrile containing internal standard and analyzed by LC–MS/MS versus a calibration curve of known concentrations.

### Efficacy studies in mice-disseminated models

*MOLM-13:* These studies complied with all relevant ethical regulations described in the guidelines established by GV-SOLAS Institutional Animal Care and Use Committee. Female CIEA-NOG mice were purchased from Taconic. Mice between 5 and 8 weeks old were tail vein injected with five million MOLM-13 cells. Treatment started 3 days after implantation. AZD5991, venetoclax, and vehicle were formulated and administered as mentioned above. Bone marrow and peripheral blood were collected 10 days post-treatment initiation, followed by red cell lysis (ACK lysis buffer), washes with FC buffer (PBS + 2% FCS) and cell staining with AquaZombie live/dead stain (cat#423102, Biolegend) containing antibodies against human CD45 (cat#564105, BDBiosciences) and human HLA-ABC (cat#561346, BDBiosciences) or isotype controls (mIgG1 cat#552834, BDBiosciences; mIgG1 cat#561504, BDBiosciences). Samples were washed with FC buffer and data acquired on an Attune NXT cytometer and analyzed using FlowJo software. Gating was performed on single/live/FCS vs. SSC/HLA-ABC vs. hCD45 double positive cells (Supplementary Figure 8).

*Eµ-Myc:* These studies complied with all ethical regulations in accordance with the guidelines established by the Institutional Animal Experimentation Ethics Committee, Peter MacCallum Cancer Center. Non-irradiated C57BL/6 mice between 6 and 8 weeks old were transplanted by tail vein injection with 100,000 GFP-labeled Eμ-Myc lymphoma cells^39^ harvested from lymph nodes of a syngeneic mouse. Treatment started 3 days after implantation. AZD5991 and vehicle were formulated and administered as mentioned above. Peripheral blood was collected by retro-orbital sampling 11 days post-transplantation and GFP-expressing leukemic cells assessed by flow cytometry using a BD FACSCanto II cytometer and data analyzed using Flowlogic software. Gating strategy is shown in Supplementary Figure 8.

## Electronic supplementary material

Supplementary Information

Description of Additional Supplementary Files

Supplementary Data 1

## Data Availability

All relevant data is available from the authors upon reasonable request. The crystal structures have been deposited to the RCSB protein data bank: [https://www.rcsb.org].
