# Supplementary material for: Discovery of Mcl-1-specific inhibitor AZD5991 and preclinical activity in multiple myeloma and acute myeloid leukemia
Source: Nat Commun. 2018 Dec 17;9:5341. doi: 10.1038/s41467-018-07551-w (PMC6297231; doi:10.1038/s41467-018-07551-w)
Supplement: Supplementary file 1 — Supplementary Information [file 41467_2018_7551_MOESM1_ESM.pdf]

Supplementary Information

Discovery of Mcl-1-specific inhibitor AZD5991 and preclinical activity in multiple myeloma and acute myeloid leukemia

Tron et al.

Supplementary Figure 1

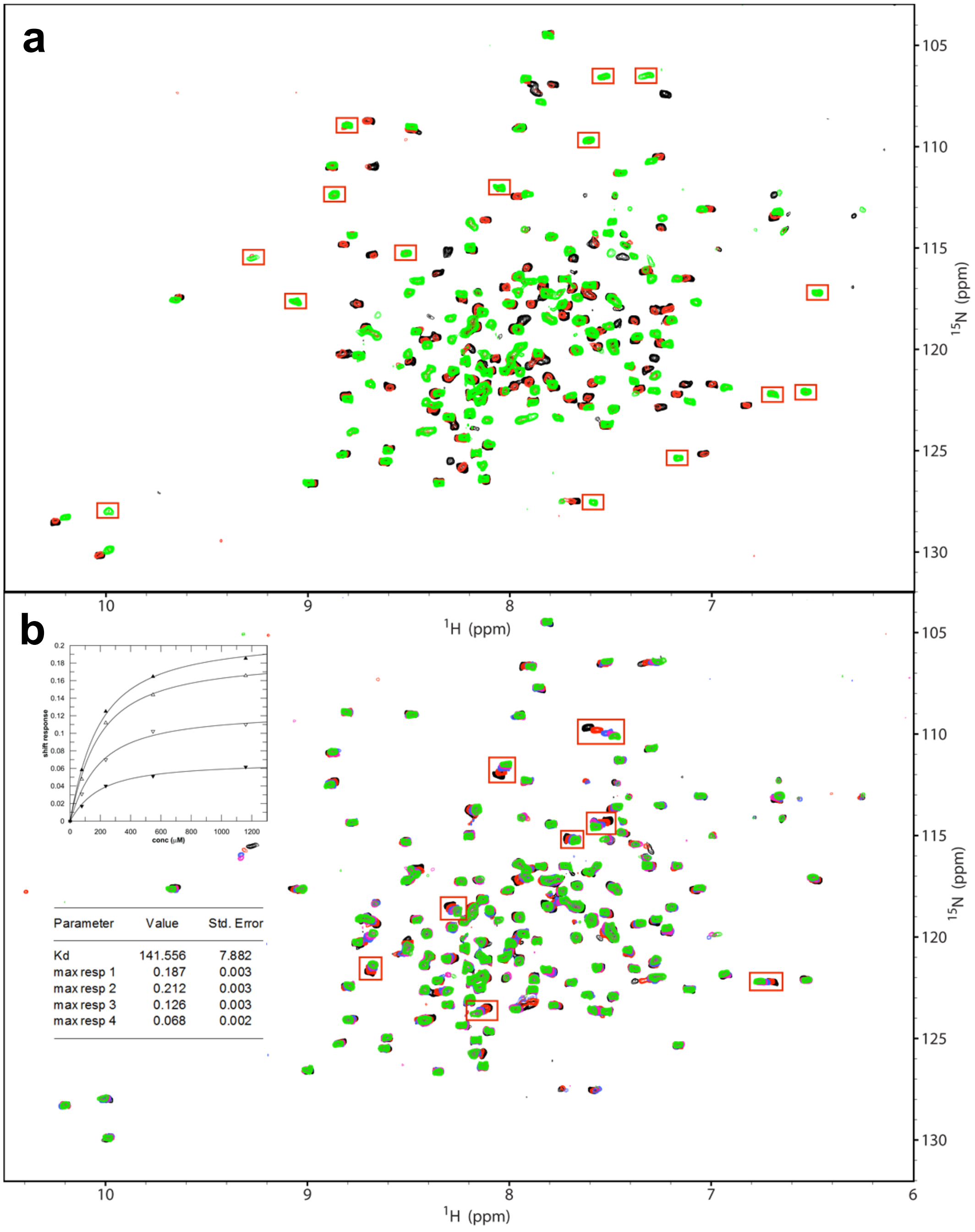

Supplementary Figure 1. 2D NMR titration of 2 binding to Mcl-1 (a) TROSY spectrum of Mcl-1, 80 μM, titrated with **2** at concentrations of 0, 40, 80 μM (black, red and green peaks, respectively) show slow exchange binding on the NMR time scale consistent with binding at sub 5 μM affinity (some of the new peaks that occur upon binding are indicated by red boxes) (b) titration of **2** at concentrations of 80, 158, 316, 628 and 1228 μM (black, red, blue, magenta and green peaks, respectively). Additional, fast exchange shifts (some shifts highlighted with red boxes) indicate a second binding event. Inset plot illustrates the simultaneous fitting of 4 shifting resonances with the determined affinity of 141 ± 8 μM for the second binding event.

Supplementary Table 1

Compound	**2**	**4**	**5**	**6**	(*R*$_a$)-**7**	(*S*$_a$)-**7**	**8** A-1210477
MCL1 SPR pK_d ± SD (n)	6.19 ± 0.22 (12)	7.01 ± 0.14 (5)	7.33 ± 0.08 (6)	8.10 ± 0.62 (8)	9.78 ± 0.21 (23)	6.01 ± 0.01 (3)	7.94 ± 0.30 (4)
MCL1 FRET pIC_{50} ± SD (n)	6.54 ± 0.28 (6)	7.37 ± 0.18 (10)	7.72 ± 0.23 (8)	8.37 ± 0.04 (4)	9.14 ± 0.22 (4)	5.2 (1)	8.20 ± 0.07 (3)
MOLP-8 caspase pEC_{50} ± SD (n)	<4.5 (6)	<4.5 (9)	<4.5 (2)	6.18 ± 0.37 (6)	7.48 ± 0.12 (11)	<4.8 (7)	5.45 ± 0.18 (18)
MV4-11 caspase pEC_{50} ± SD (n)	<4.5 (4)	<4.7 (6)	<4.5 (2)	5.98 ± 0.53 (3)	7.62 ± 0.29 (14)	<4.8 (5)	5.73 ± 0.26 (19)
NCI-H23 caspase pEC_{50} ± SD (n)	<4.5 (2)	<4.5 (3)	nd	5.24 ± 0.34 (2)	6.71 ± 0.14 (19)	<4.6 (3)	4.68 ± 0.10 (6)

nd = not determined

Supplementary Table 1. Repeat data and standard deviations. Individual measurements of K_d, IC_{50} and EC_{50} data were log transformed and standard deviation (SD) was calculated. Standard deviations were not calculated where one or more of the data EC_{50}s was reported beyond the concentration range tested. Number of data points used to calculate mean and standard deviation are shown in parentheses.

Supplementary Figure 2.

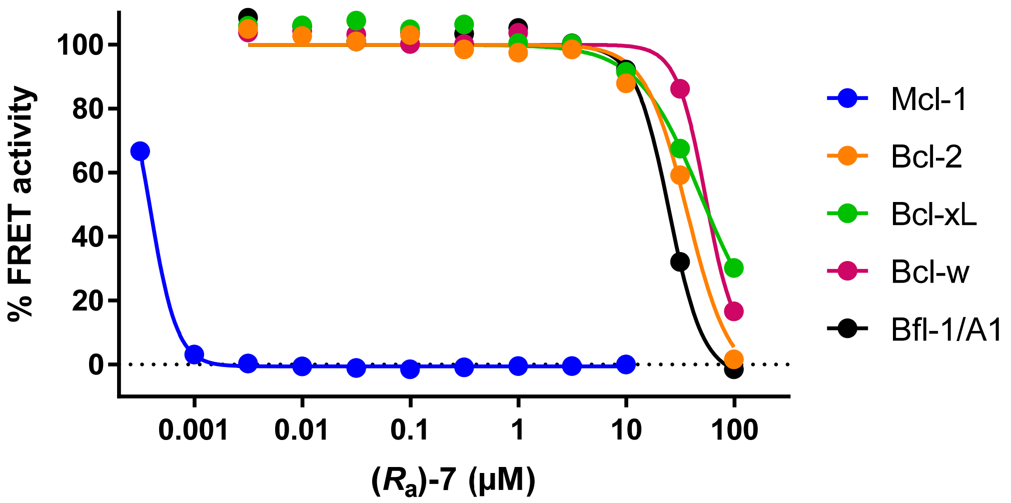

Supplementary Figure 2. (R_a)-7 is a potent inhibitor and selective for Mcl-1 vs. other Bcl-2 family members. Binding activity of (R_a)-7 to Bcl-2 pro-survival proteins determined by TR-FRET binding assays performed with increasing concentrations of the compound.

Supplementary Figure 3

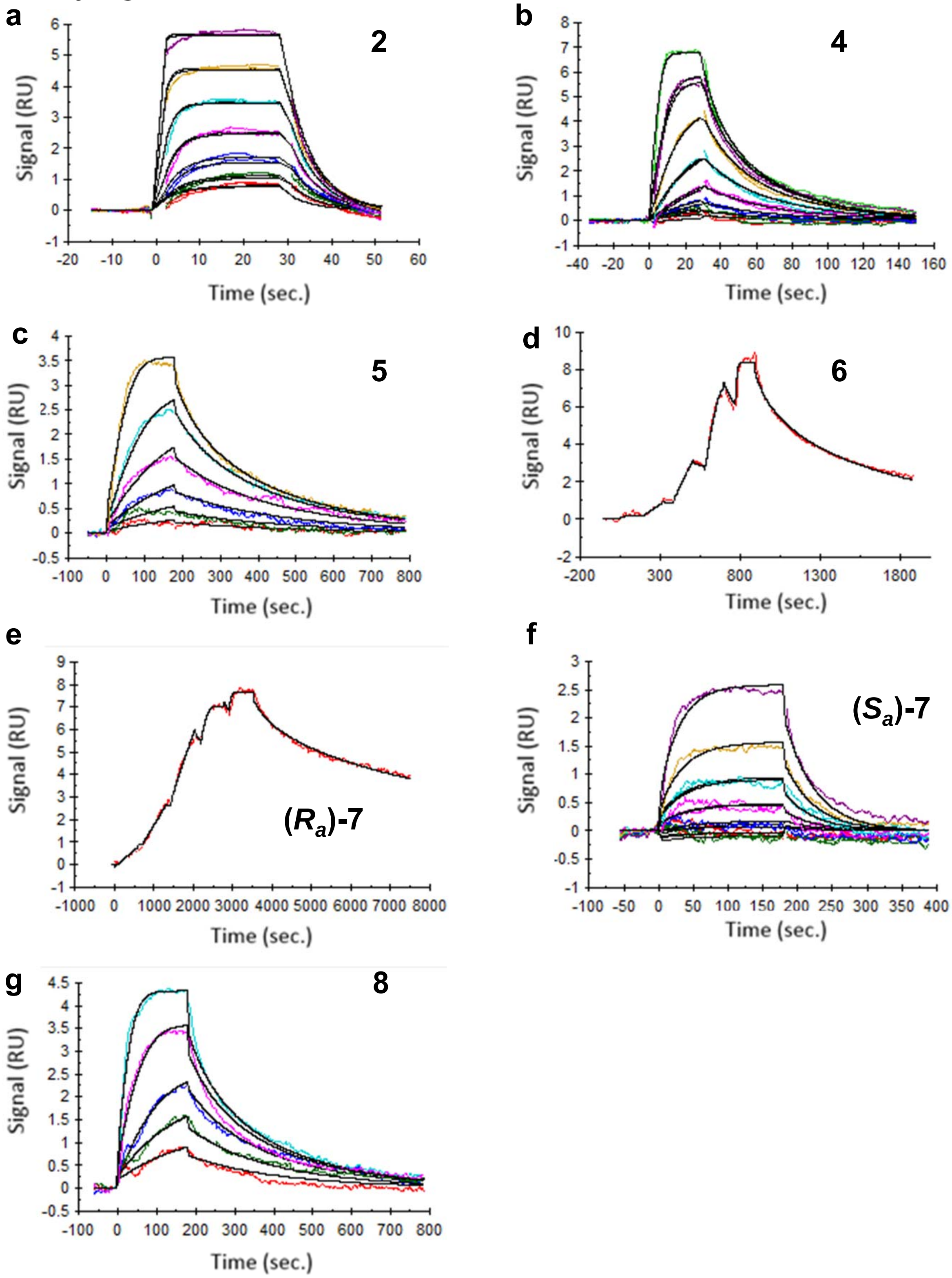

Supplementary Figure 3: Sensorgrams of compounds 2, and 4-8 binding to Mcl-1 by SPR
Sensorgrams depict SPR binding signal over time for varying concentrations of compounds **2** and **4-8** as coloured lines and the global fit as black lines. (a-c and f-g) Sensorgrams for compounds **2**, **4-5**, (S_a)-**7** and **8** were generated using multi-cycle kinetics. The association phase starts at time zero and dissociation phase starts at either 30 or 180 seconds. (d-e) Due to slow dissociation kinetics, binding data for compounds **6** and (R_a)-**7** were generated using single-cycle kinetics by serial injections of five increasing concentrations followed by a single dissociation phase.

Supplementary Figure 4

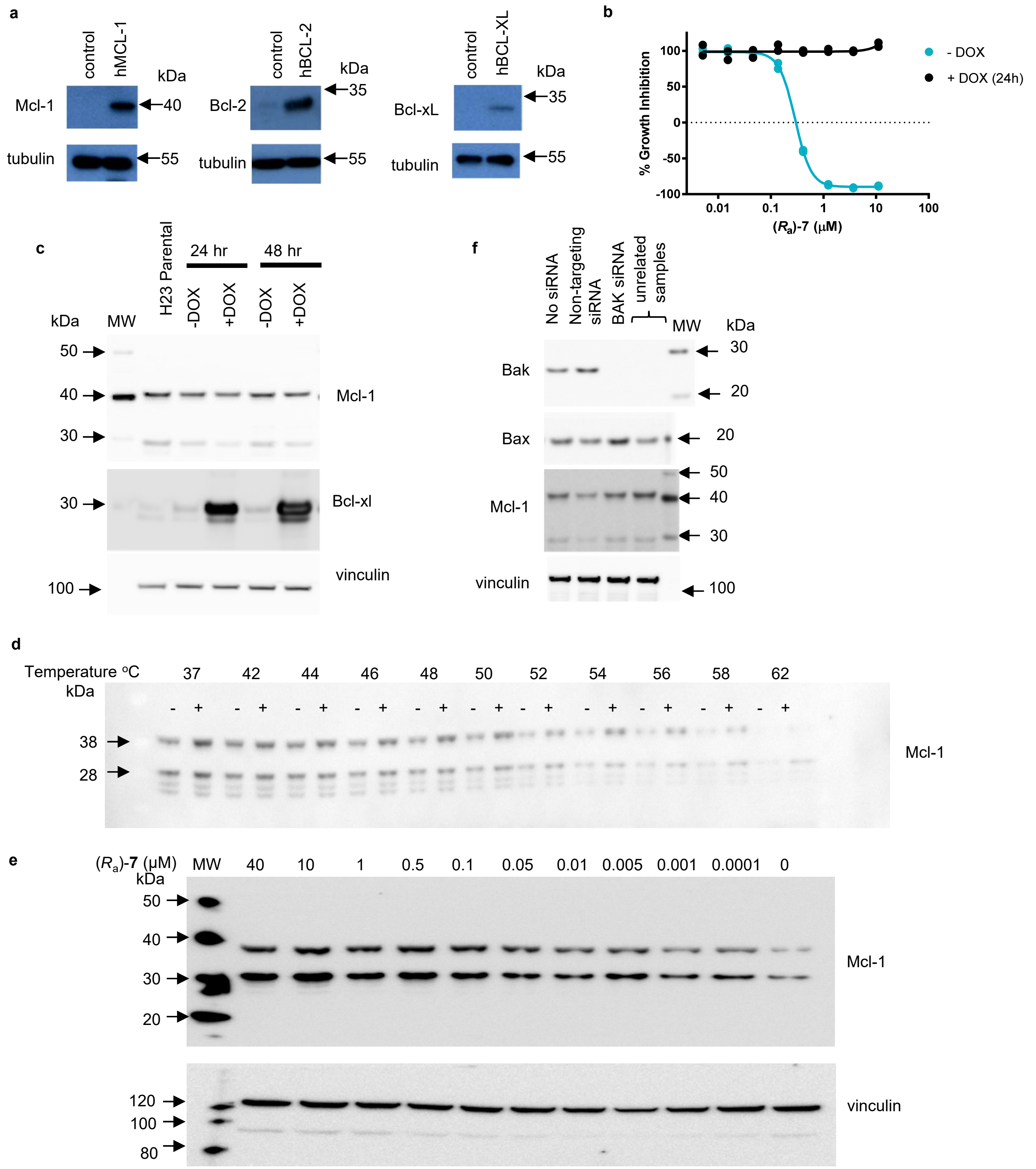

Supplementary Figure 4. Mechanism of action of (R_a)-7. (a) Overexpression of human Mcl-1, Bcl-2 and Bcl-xL proteins in Eμ-Myc lymphoma cells assessed by immunoblotting. (b) NCI-H23 cells stably expressing doxycycline-inducible Bcl-xL were treated with doxycycline for 24 h followed by a 48 h incubation with (R_a)-**7** at various concentrations from 0.005 μM to 11.110 μM. Cell growth was determined using CellTiter Glo and GraphPad Prism for data analysis. Data shown is the average of 2 experiments. (c) Overexpression of Bcl-xL in NCI-H23 cells was evaluated by immunoblotting. (d) MV4-11 cells were treated with (R_a)-**7** at 40 μM (+) or left untreated (-) for 15 minutes at the indicated temperatures followed by western blotting analysis. (e) MV4-11 cells were treated with increasing concentrations of (R_a)-**7** for 15 minutes at 48ºC followed by western blotting analysis (isothermal dose-response assay). (f) Immunoblot showing protein expression levels of Bak in non-targeting and siBak transfected NCI-H23 cells. MW: molecular weight marker.

Supplementary Figure 5

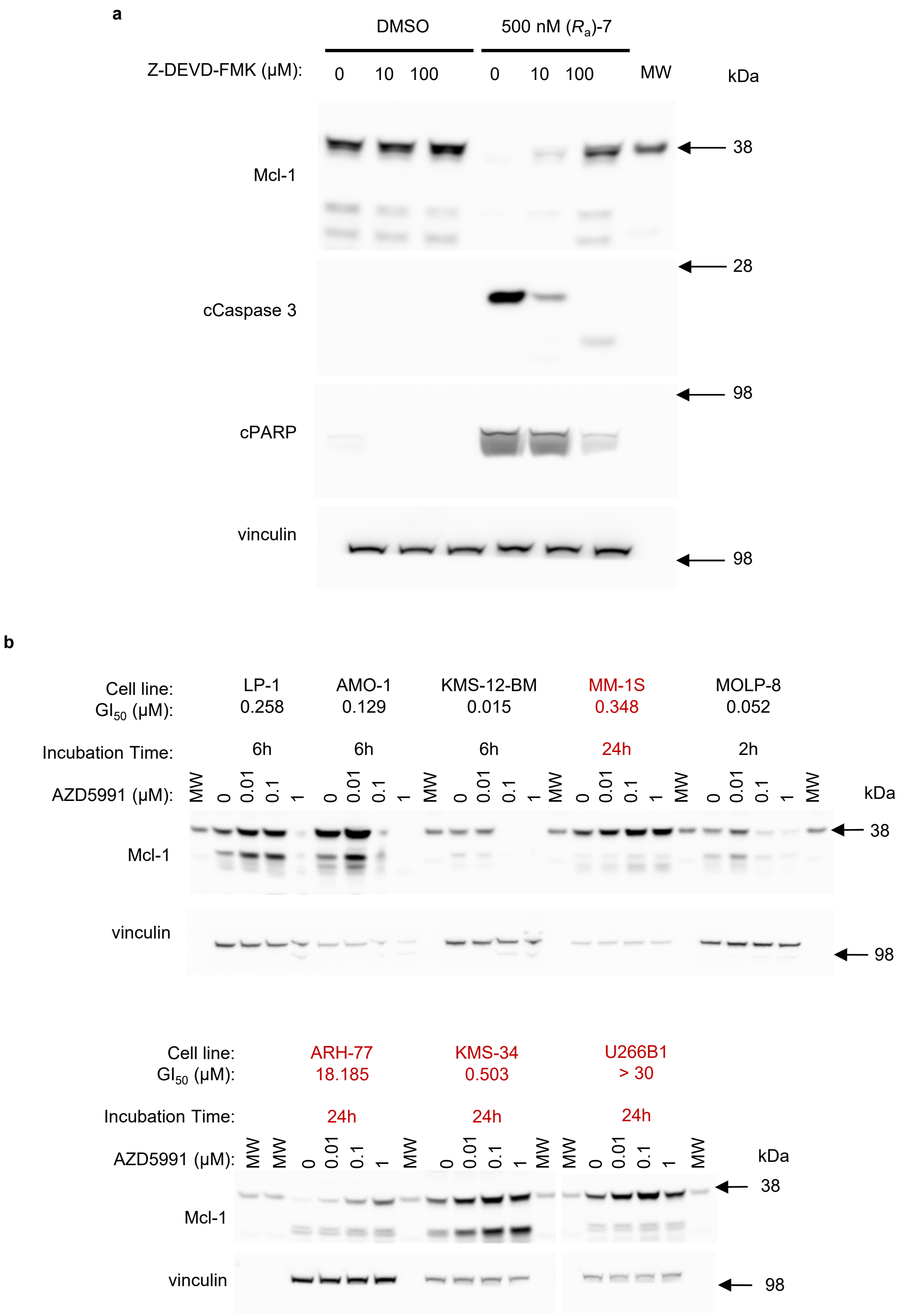

Supplementary Figure 5. Inhibition of caspase 3 activity prevents reduction of Mcl-1 protein level in (*R*$_a$)-7 (AZD5991) sensitive cell lines. (a) MOLP-8 cells were treated with the caspase 3 inhibitor Z-DEVD-FMK at the indicated concentrations for 4 h prior to addition of (*R*$_a$)-**7** at 500 nM or DMSO control for the last 2 h of treatment and analyzed by immunoblot (b) MM cell lines were treated with AZD5991 for the indicated times before assessing the protein level of Mcl-1 by immunoblotting. The incubation times for the sensitive cell lines labelled in black correspond to the time when maximum effect on caspase 3 activation is achieved, as determined by kinetic studies (data not shown). Cell lines less sensitive or insensitive to AZD5991 are labeled in red and were treated for 24 h.

Supplementary Figure 6

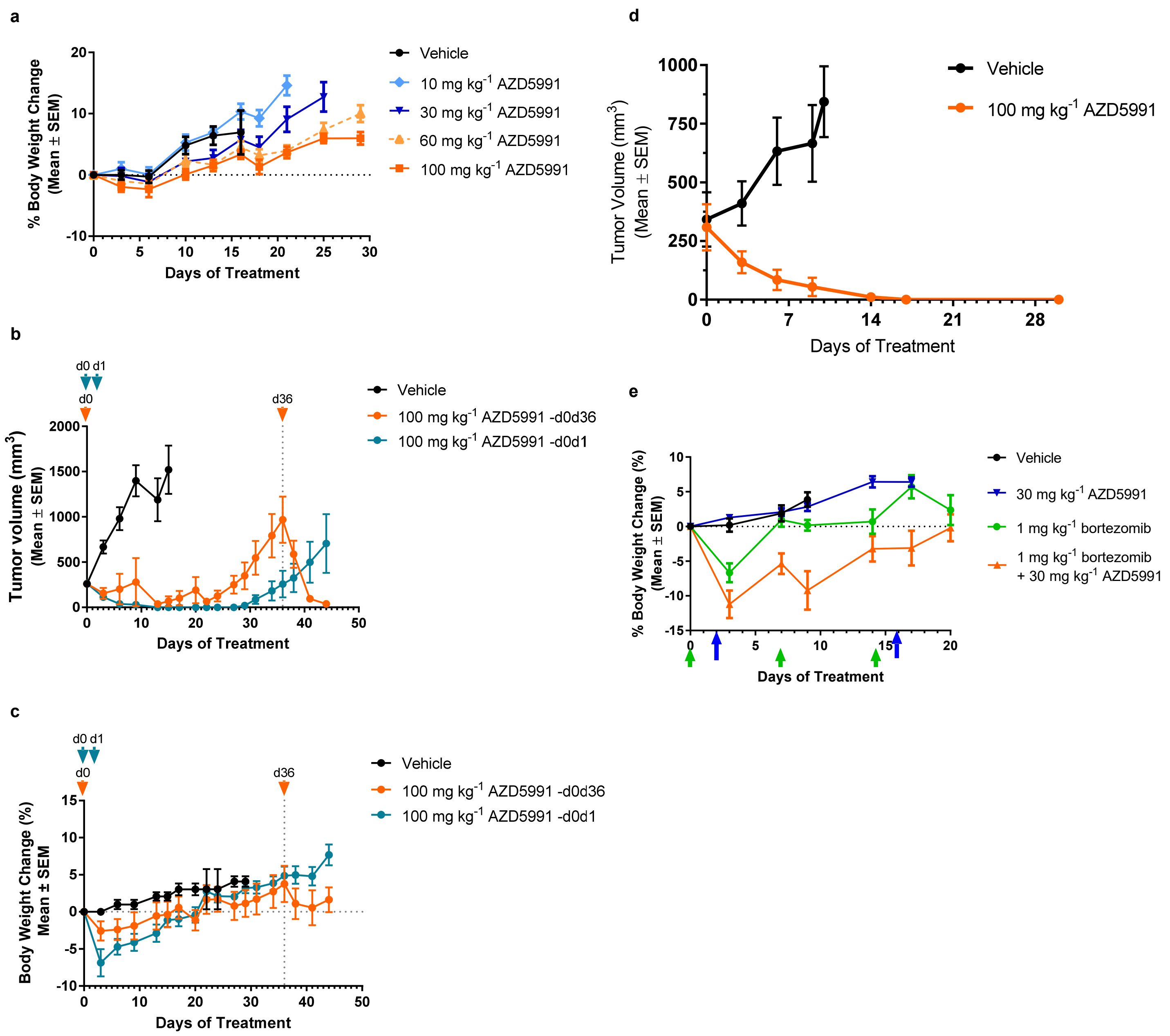

Supplementary Figure 6. Activity of AZD5991 in MM mice xenograft models as single agent or in combination with bortezomib. (a) Body weight evaluation of mice harboring MOLP-8 tumors that were treated with a single i.v. dose of vehicle or AZD5991 at 10 mg kg^{-1}, 30 mg kg^{-1}, 60 mg kg^{-1} or 100 mg kg^{-1}. Mean ± SEM body weight of 7 animals per treatment group are shown. Subcutaneous tumor growth (b) and body weight evaluation (c) in mice harboring MOLP-8 subcutaneous tumors treated with AZD5991 i.v. at 100 mg kg^{-1} on day 0 and day 1 (light blue) or day 0 and day 36 (orange). Tumor volumes and % body weight change are presented as mean ± SEM, 7 mice were evaluated per group. (d) Tumor growth evaluation in mice harboring NCI-H929 subcutaneous tumors treated with a single i.v. dose of AZD5991 at 100 mg kg^{-1} or vehicle. Mean ± SEM tumor volume of 4 animals per treatment group are shown. (e) Body weight evaluation of mice harboring NCI-H929 subcutaneous tumors that were treated with AZD5991 every other week or bortezomib once weekly as single agents or in combination. Arrows indicate day of dosing for AZD5991 (blue) and bortezomib (green). Mean ± SEM body weight of 6 animals per treatment group are shown.

Cell Line	AZD5991 6h caspase EC_{50}	venetoclax 6h caspase EC_{50}	AZD5991 + venetoclax 6h caspase EC_{50}
NOMO1	0.03248	> 0.333	0.00885
OCIAML5	> 0.333	0.07305	0.00492
NB4	0.20083	0.02750	0.00333
MV411	0.05632	0.04522	0.00333
MOLM13	0.10796	0.00966	0.00333
ME1	> 0.333	0.01024	0.00333
OCIAML3	> 0.333	> 0.333	0.07032
KG1	> 0.333	0.26867	0.23540
CMK	> 0.333	> 0.333	> 0.333
HEL9217	> 0.333	> 0.333	> 0.333
OCIM1	> 0.333	> 0.333	0.21141

Supplementary Table 2. AZD5991 and venetoclax activity in AML cancer cell lines as single agent or in combination. AML cancer cell lines were treated with a range of concentrations of AZD5991 and venetoclax as single agents or with AZD5991 in combination with 160 nM venetoclax and caspase 3/7 activation was assessed by CaspaseGlo after 6 h of treatment. Data analysis was performed using GraphPad Prism. Data shown are representative of ≥ 2 independent experiments.

Supplementary Figure 7

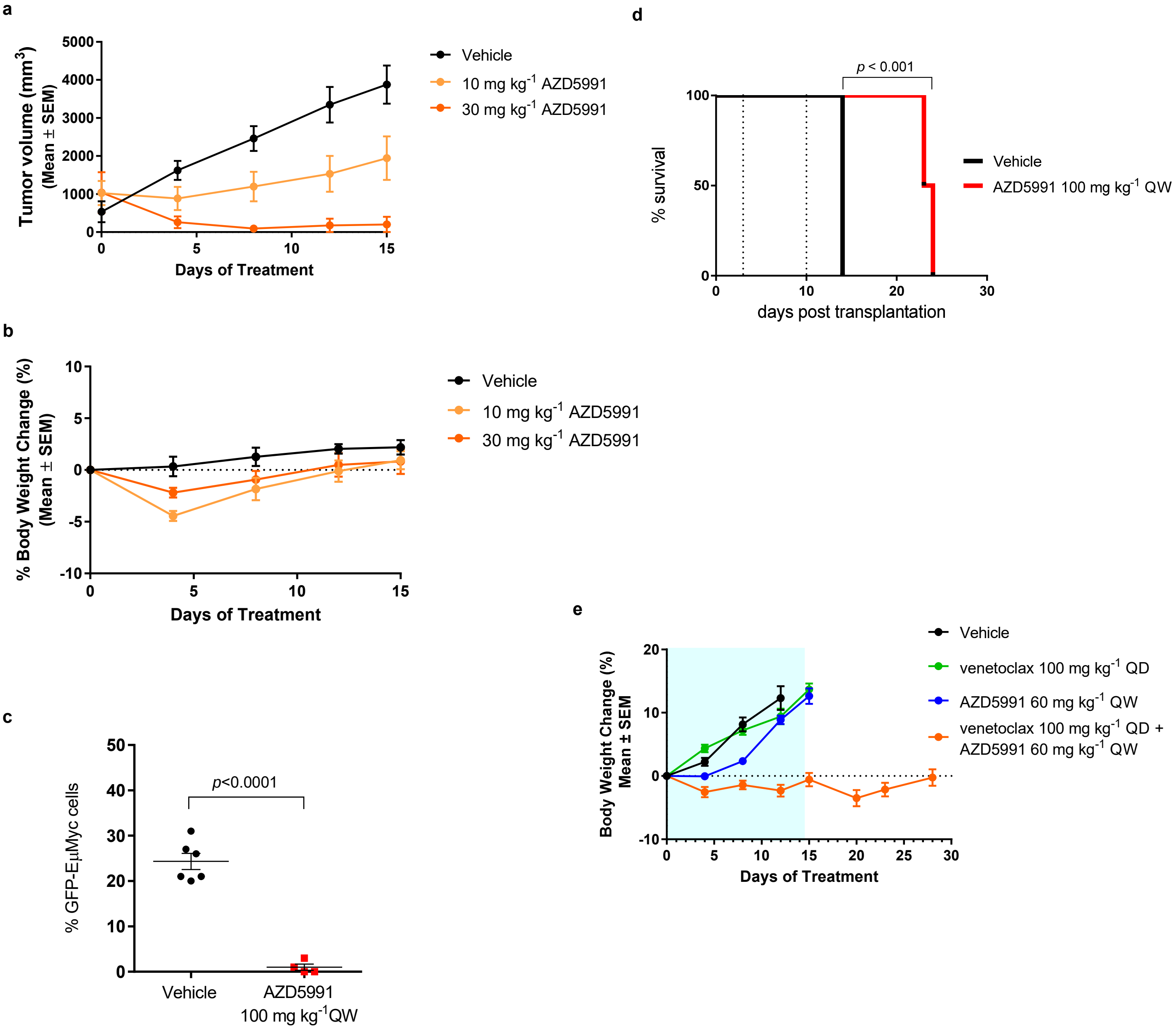

Supplementary Figure 7. Activity of AZD5991 in AML xenograft models as single agent or in combination with venetoclax. Subcutaneous tumor growth (a) and body weight change (b) in the MV4-11 rat tumor model treated with a single i.v. dose of AZD5991 at 10 or 30 mg kg^{-1}. Tumor volumes and % body weight change are presented as mean ± SEM, 3 rats were evaluated per group. (c) Leukemic cells were evaluated in peripheral blood from mice engrafted with GFP-labeled Eμ-Myc leukemic cells and treated with vehicle (n=6) or AZD5991 at 100 mg kg^{-1} once weekly (n=4). Analysis were performed on day 11 after transplantation. A non-parametric, unpaired, two-tailed t-test was used to calculate significance (d) Kaplan-Meier curve showing overall survival of mice engrafted with GFP-labeled Eμ-Myc leukemic cells and treated with vehicle (n=6) or AZD5991 at 100 mg kg^{-1} once weekly (n=4). Dashed lines depict days of AZD5991 i.v. administration. A non-parametric, unpaired, two-tailed t-test was used to calculate significance. (e) Body weight evaluation of mice harboring OCI-AML3 subcutaneous tumors that were treated with AZD5991 (i.v.) once a week or venetoclax (oral) every day as single agents or in combination. Mean ± SEM of % body weight change of 10 animals per treatment group are shown.

Supplementary Figure 8

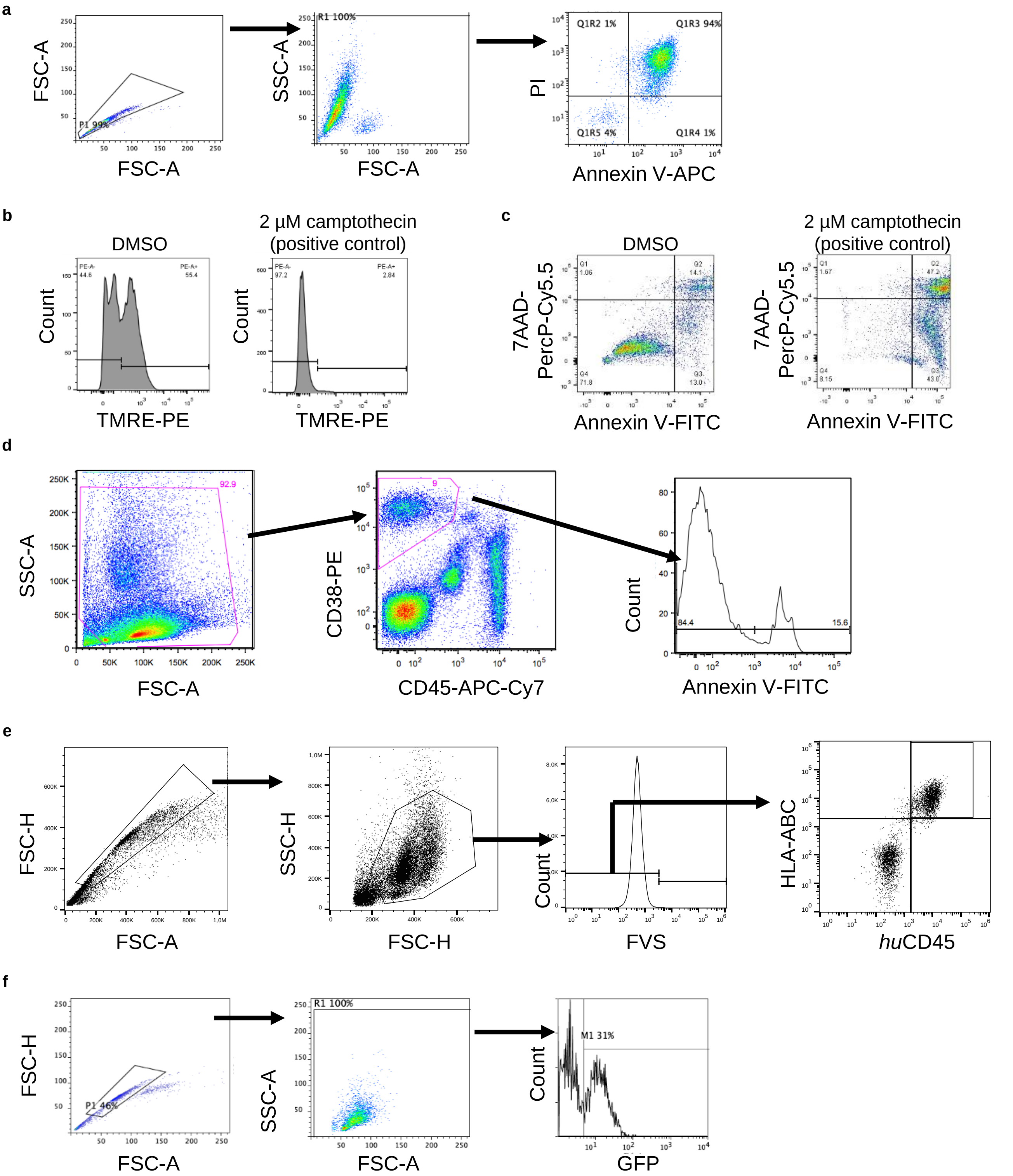

Supplementary Figure 8. Gating strategy used for cell analysis. (a) Gating strategy to assess the percentage of apoptotic (Annexin V/PI) cells shown in Fig. 3a. Gating strategy to determine the percentage of TMRE (MOMP) negative cells (b), and Annexin V (PS externalization) and 7-AAD (membrane permeabilization) positive cells (c) presented on Fig. 4b. (d) Gating strategy to assess apoptosis in primary MM cells (Annexin V +) shown in Fig. 6.d (e) Gating strategy to determine the percentage of HLA-ABC^{+} hCD45^{+} leukemic cells present in peripheral blood and bone marrow of mice engrafted with MOLM13 (AML) cells. Data shown in Fig. 7c-d. FVS: Fixable Viability Dye. (f) Gating strategy to assess the percentage of GFP-labeled cells in peripheral blood of mice engrafted with GFP-labeled Eµ-Myc leukemic cells. Data presented in Supplementary Fig. 7c

Supplementary Figure 9

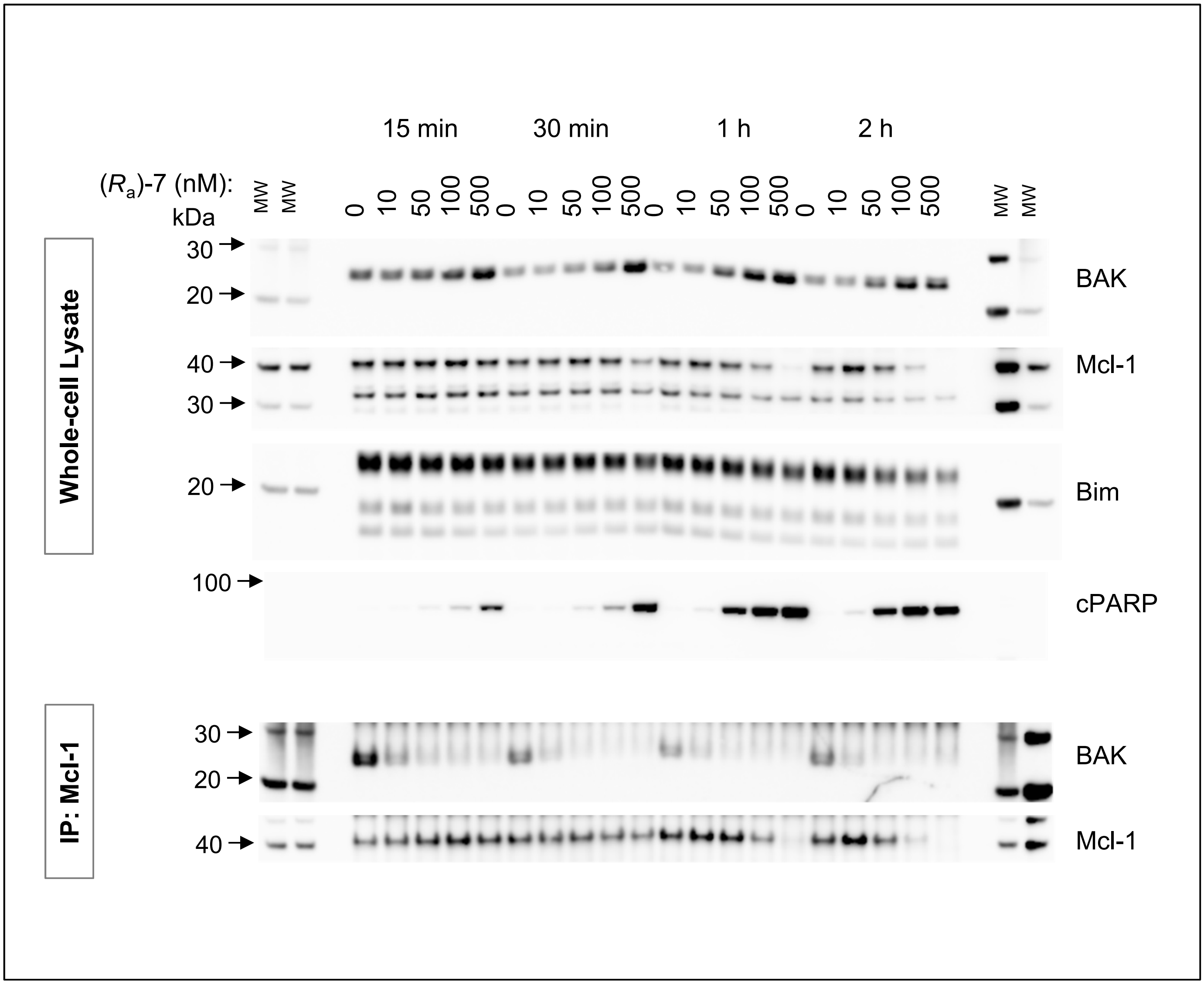

Supplementary Figure 9. Uncropped gel images corresponding to Fig. 4a

Supplementary Figure 10

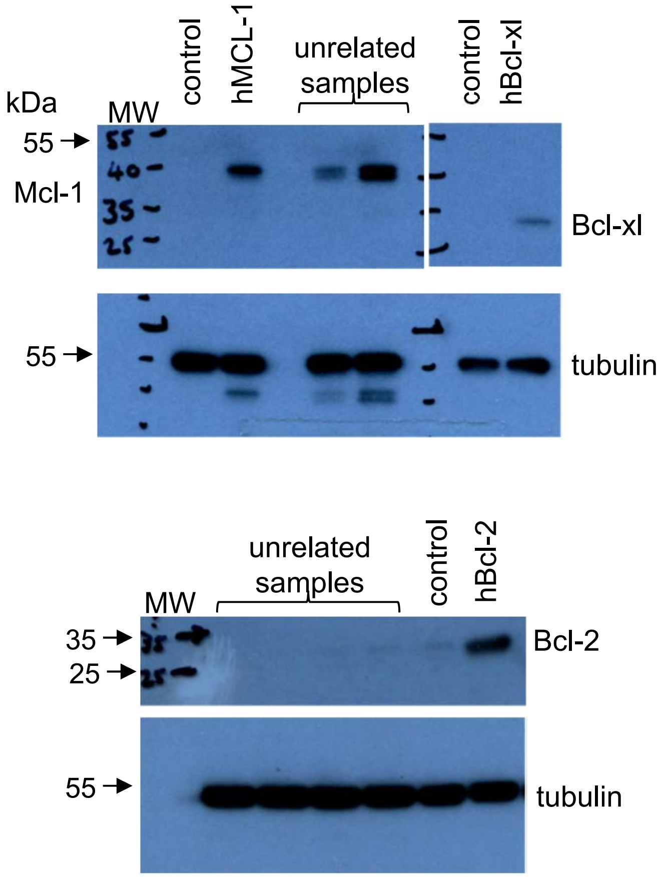

Supplementary Figure 10. Uncropped gel images corresponding to Supplementary Fig. 4a.

Supplementary Figure 11

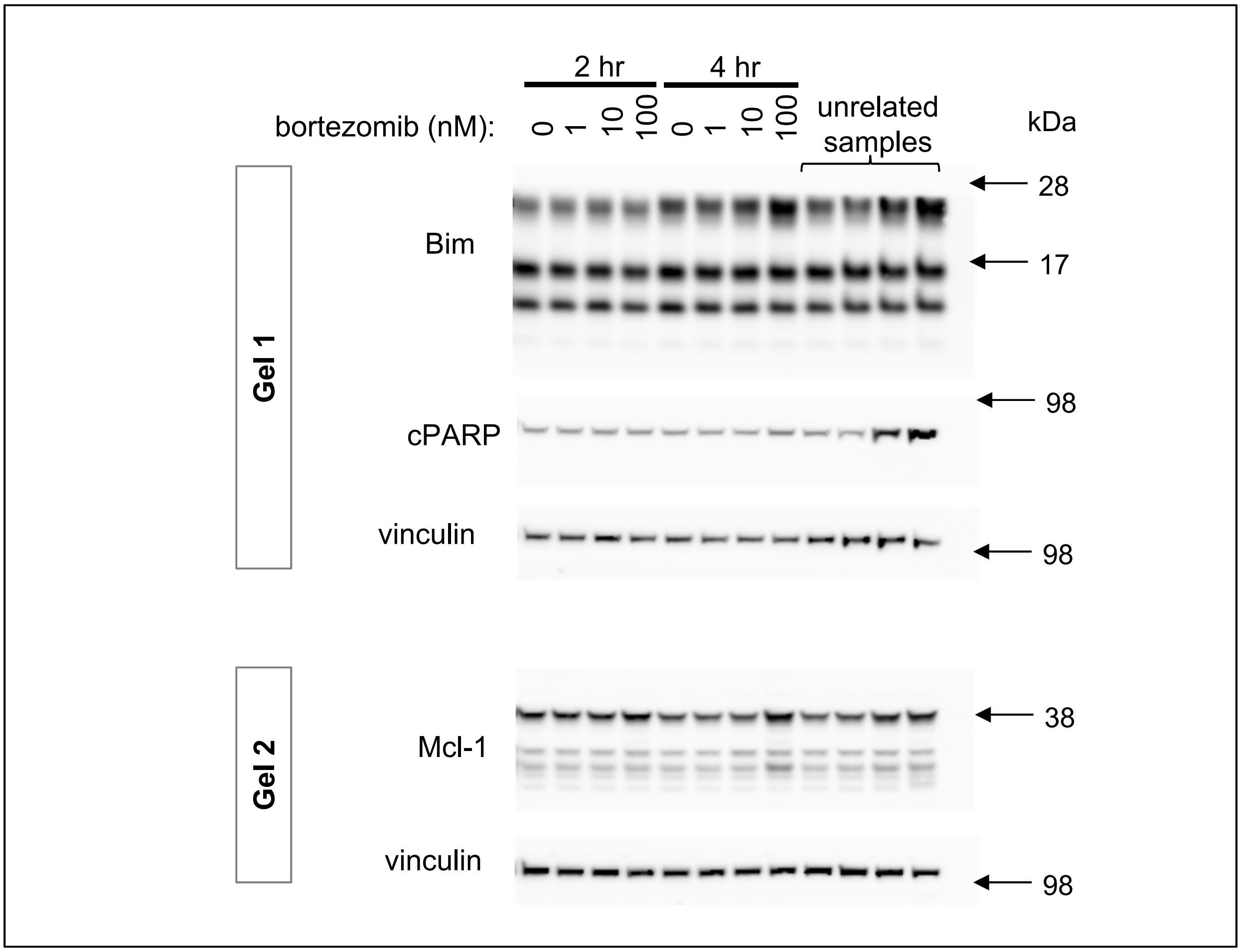

Supplementary Figure 11. Uncropped gel images corresponding to Fig. 6e

Supplementary Figure 12

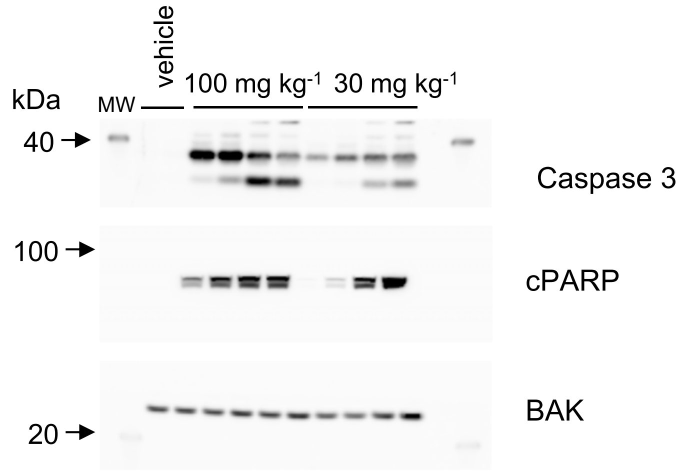

Supplementary Figure 12. Uncropped gel images corresponding to Fig. 7b

Supplementary Figure 13

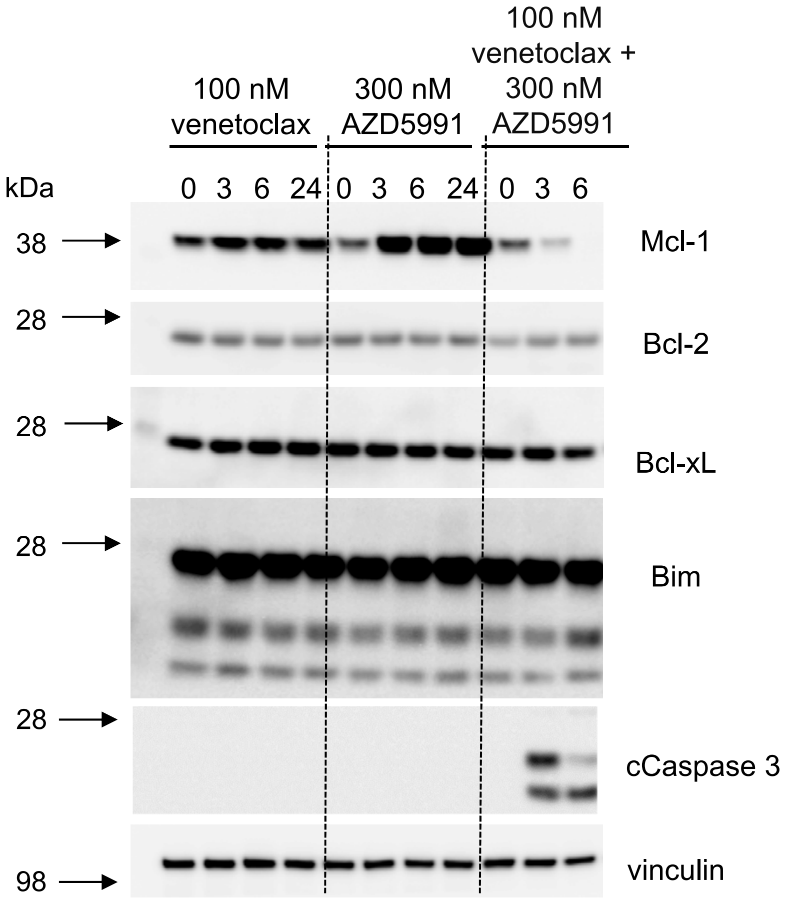

Supplementary Figure 13. Uncropped gel images corresponding to Fig. 7f

Synthesis and characterization of compounds 3, 4, 5, 6 and 7

General Synthetic Methods

Compounds **1**, **2** and **8** were synthesized by procedures reported in the literature.[1,2]

Unless stated otherwise:

(i) all syntheses were carried out at ambient temperature, *i.e.* in the range 17 to 25 ºC and under an atmosphere of an inert gas such as nitrogen unless otherwise stated;

(ii) evaporations were carried out by rotary evaporation or utilizing Genevac equipment or Biotage v10 evaporator under reduced pressure;

(iii) silica gel chromatography purifications were performed on an automated Teledyne Isco CombiFlash® Rf or Teledyne Isco CombiFlash® Companion® using prepacked RediSep Rf Gold™ Silica Columns (20-40 µm, spherical particles), GraceResolv™ Cartridges (Davisil® silica) or Silicycle cartridges (40 - 63 µm).

(iv) chiral preparative chromatography was performed on a Waters Prep 100 SFC-MS instrument with MS- and UV- triggered collection or a Thar MultiGram III SFC instrument with UV collection.

(v) chiral analytical chromatography was performed on either a Waters X5 SFC-MS with UV detection or a Waters UPC2 SFC-MS with UV and ELSD detection.

(vi) NMR chemical shift values were measured on the delta scale, using the solvent residual peak as the internal standard [proton magnetic resonance spectra were determined using a Bruker Avance 500 (500 MHz), Bruker Avance 400 (400 MHz), Bruker Avance 300 (300 MHz) or Bruker DRX (300 MHz) instrument]; measurements were taken at ambient temperature unless otherwise specified; the following abbreviations have been used: s, singlet; d, doublet; t, triplet; q, quartet; m, multiplet; dd, doublet of doublets; ddd, doublet of doublet of doublet; dt, doublet of triplets; bs, broad signal; AB. d., AB double.

(vii) UPLC-MS were carried out using a Waters UPLC fitted with a Waters SQ mass spectrometer (Column temp 40 ºC, UV = 220-300 nm or 190-400 nm, Mass Spec = ESI with positive/negative switching) at a flow rate of 1 mL/min using a solvent system of 97% A + 3% B to 3% A + 97% B over 1.50 min (total run time with equilibration back to starting conditions, etc., 1.70 min), where A = 0.1% formic acid or 0.05% trifluoroacetic acid in water (for acidic work) or 0.1% ammonium hydroxide in water (for basic work) and B = acetonitrile. For acidic analysis the column used was a Waters Acquity HSS T3 (1.8 µm, 2.1 x 50 mm), for basic analysis the column used was a Waters Acquity BEH C18 (1.7 µm, 2.1 x 50 mm). Alternatively, UPLC was carried out using a Waters UPLC fitted with a Waters SQ mass spectrometer (Column temp

30 °C, UV = 210-400 nm, Mass Spec = ESI with positive/negative switching) at a flow rate of 1mL/min using a solvent gradient of 2 to 98% B over 1.5 min (total run time with equilibration back to starting conditions 2 min), where A = 0.1% formic acid in water and B = 0.1% formic acid in acetonitrile (for acidic work) or A = 0.1% ammonium hydroxide in water and B = acetonitrile (for basic work). For acidic analysis the column used was a Waters Acquity HSS T3 (1.8 µm, 2.1 x 30 mm), for basic analysis the column used was a Waters Acquity BEH C18 (1.7 µm, 2.1 x 30 mm); The reported molecular ion corresponds to the [M+H]+ unless otherwise specified; for molecules with multiple isotopic patterns (Br, Cl, etc.) the reported value is the one obtained with highest intensity unless otherwise specified.

(viii) intermediate purity was assessed by thin layer chromatographic, mass spectroscopy, liquid chromatography/mass spectroscopy (LCMS), ultra high performance liquid chromatography/mass spectroscopy (UPLC/MS), high performance liquid chromatography (HPLC) and/or nuclear magnetic resonance (NMR) analysis;

(ix) the following abbreviations have been used:

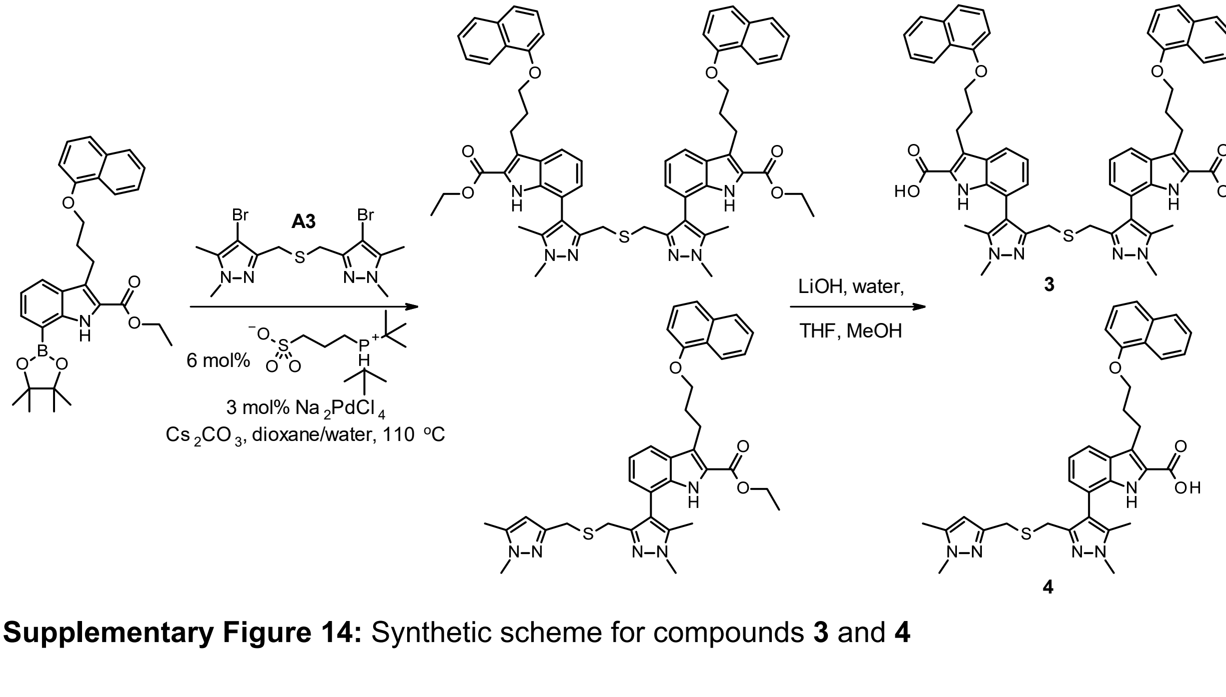

Supplementary Figure 14: Synthetic scheme for compounds **3** and **4**

(4-Bromo-1,5-dimethyl-1H-pyrazol-3-yl)methanol (**A1**)

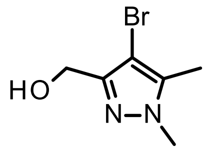

N-Bromo succinimide (NBS, 47.4 g, 266 mmol) was added portionwise over 30 min to a solution of (1,5-dimethyl-1H-pyrazol-3-yl)methanol (32.0 g, 253 mmol) in dichloromethane (DCM, 500 mL) at 0 °C. The resulting mixture was stirred at 25 °C for 1 h. The reaction mixture was diluted with DCM (200 mL), and washed sequentially with water (250 mL) and brine (150 mL). The organic layer was dried over Na_2SO_4, filtered and concentrated to afford a residue which was washed with petroleum ether (PE)/EtOAc (1:1) (10 mL) to afford (4-bromo-1,5-dimethyl-1H-pyrazol-3-yl)methanol **A1** (48.0 g, 92.0%), which was used without further purification.

^{1}H NMR (300 MHz, CHLOROFORM-*d*) δ 2.08 (s, 1H), 2.26 (s, 3H), 3.79 (s, 3H), 4.63 (s, 2H); ^{13}C NMR (75 MHz, CHLOROFORM-*d*) δ 10.05, 37.03, 57.18, 92.45, 137.95, 148.34; m/z (electrospray mode (ES$^+$)), [M+H]$^+$ calcd for $C_6H_9BrN_2O$, 204.99; found 205.0.

4-Bromo-3-(chloromethyl)-1,5-dimethyl-1H-pyrazole (**A2**)

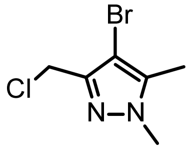

(4-Bromo-1,5-dimethyl-1H-pyrazol-3-yl)methanol **A1** (2.08 g, 10.1 mmol) in anhydrous DCM (20 mL) was stirred at 0 °C. Thionyl chloride (1.10 mL, 15.2 mmol) was added. The solution was allowed to warm to room temperature (RT) and stirred at RT for 1 h. The reaction mixture was concentrated to dryness. DCM (20 mL) was added. The resulting organic solution was washed with sat. $NaHCO_3$ solution, dried over Na_2SO_4, filtered and concentrated to dryness to give 4-bromo-3-(chloromethyl)-1,5-dimethyl-1H-pyrazole **A2** (2.20 g, 97.0%) as a white solid.

^{1}H NMR (300 MHz, CHLOROFORM-*d*) δ 2.21 (s, 3H), 3.74 (s, 3H), 4.59 (s, 2H); ^{13}C NMR (75 MHz, CHLOROFORM-*d*) δ 10.24, 37.15, 37.34, 93.77, 138.34, 145.23; m/z (ES$^+$), [M+H]$^+$ calcd for $C_6H_8BrClN_2$, 222.99; found 222.9.

Bis((4-bromo-1,5-dimethyl-1H-pyrazol-3-yl)methyl) sulfane (**A3**)

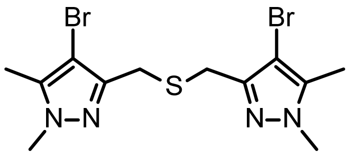

A solution of Na_2S (128 mg, 1.64 mmol) in acetone (4 mL) and H_2O (1.5 mL) was added to a solution of 4-bromo-3-(chloromethyl)-1,5-dimethyl-1H-pyrazole **A2** (447 mg, 2.00 mmol) in acetone (4 mL), then MeOH (10 mL) was added. After stirring at RT overnight, the mixture was concentrated to dryness. The residue was purified by silica gel column chromatography (hexanes/EtOAc) to give bis((4-bromo-1,5-dimethyl-1H-pyrazol-3-yl)methyl)sulfane **A3** (383 mg, 94.0 %).

^{1}H NMR (300 MHz, CHLOROFORM-*d*) δ 2.21 (s, 6H), 3.72 (s, 4H), 3.75 (s, 6H); ^{13}C NMR (75 MHz, CHLOROFORM-*d*) δ 10.28, 27.52, 37.13, 93.42, 137.72, 146.10; m/z (ES$^+$), [M+H]$^+$ calcd for $C_{12}H_{16}Br_2N_4S$, 406.95; found 406.9.

7,7'-((Thiobis(methylene))bis(1,5-dimethyl-1H-pyrazole-3,4-diyl))bis(3-(3-(naphthalen-1-yloxy)propyl)-1H-indole-2-carboxylic acid (**3**)

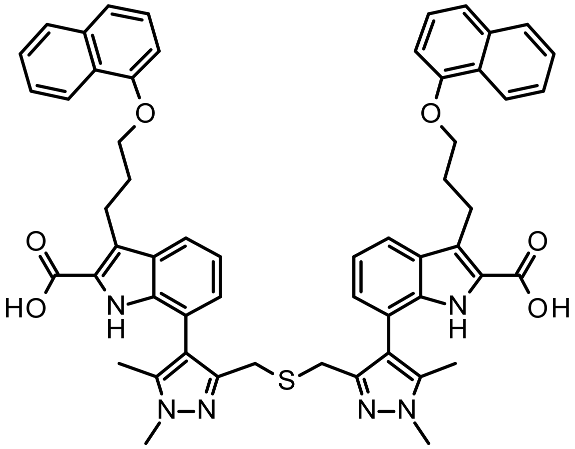

7-(3-(((1,5-Dimethyl-1H-pyrazol-3-yl)methoxy)methyl)-1,5-dimethyl-1H-pyrazol-4-yl)-3-

(3-(naphthalen-1-yloxy)propyl)-1H-indole-2-carboxylic acid (**4**)

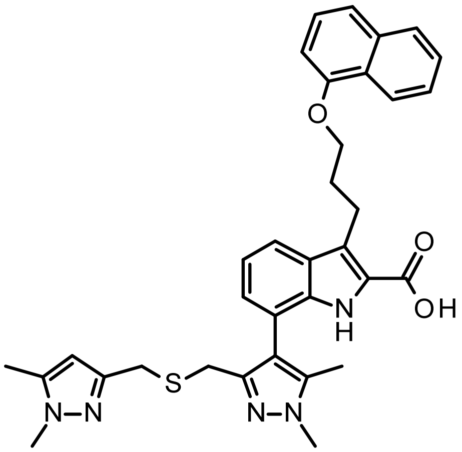

Cesium carbonate (909 mg, 2.79 mmol), 3-(di-*tert*-butylphosphanyl)propane-1-sulfonic acid (42.8 mg, 0.16 mmol), sodium tetrachloropalladate (II) (23.4 mg, 0.08 mmol), bis((4-bromo-1,5-dimethyl-1H-pyrazol-3-yl)methyl)sulfane **A3** (163 mg, 0.40 mmol), and ethyl 3-(3-(naphthalen-1-yloxy)propyl)-7-(4,4,5,5-tetramethyl-1,3-dioxolan-2-yl)-1H-indole-2-carboxylate (200 mg, 0.40 mmol) were added into a vial. Dioxane (2.00 mL) and water (0.50 mL) were added. The mixture was degassed, filled with N_2 and stirred at 110 °C for 6 h. After cooling to RT, the mixture was diluted with EtOAc (10 mL) and washed with sat. $NaHCO_3$ solution (10 mL). The organic layer was dried over $MgSO_4$ and concentrated to dryness. The residue was purified by silica gel chromatography (hexanes/EtOAc) to yield 61 mg of a mixture of diethyl 7,7'-((thiobis(methylene))bis(1,5-dimethyl-1H-pyrazole-3,4-diyl))bis(3-(3-(naphthalen-1-yloxy)propyl)-1H-indole-2-carboxylate) and ethyl 7-(3-((((1,5-dimethyl-1H-pyrazol-3-yl)methyl)thio)methyl)-1,5-dimethyl-1H-pyrazol-4-yl)-3-(3-(naphthalen-1-yloxy)propyl)-1H-indole-2-carboxylate. This mixture was dissolved in H_2O (200 µL), tetrahydrofuran (THF, 200 µL) and MeOH (200 µL). Lithium hydroxide (29.4 mg, 1.23 mmol) was added. The mixture was stirred at 60 °C for 6 h. After cooling to RT, the mixture was concentrated to dryness and acidified with 1N HCl (1.2 mL). The aqueous layer was extracted with EtOAc (3 x 2 mL). The organic phase was concentrated to dryness. The residue was purified by reverse-phase chromatography (50-95% CH_3CN in H_2O with 0.1% trifluoroacetic acid (TFA)) to give 7,7'-((thiobis(methylene))bis(1,5-dimethyl-1H-pyrazole-3,4-diyl))bis(3-(3-(naphthalen-1-yloxy)propyl)-1H-indole-2-carboxylic acid **3** (18.0 mg, 31.3%) and 7-(3-((((1,5-dimethyl-1H-pyrazol-3-yl)methyl)thio)methyl)-1,5-dimethyl-1H-pyrazol-4-yl)-3-(3-(naphthalen-1-yloxy)propyl)-1H-indole-2-carboxylic acid **4** (10.0 mg, 27.4%).

3: ^{1}H NMR (500 MHz, DMSO-d_6) δ 1.99 (s, 6H), 2.21 (s, 4H), 3.40 - 3.78 (m, 14H), 4.20 (s, 4H),

6.79 - 7.08 (m, 6H), 7.26 - 7.72 (m, 12H), 7.76 - 7.95 (m, 2H), 8.09 - 8.30 (m, 2H), 11.29 - 11.50 (m, 2H); ^{13}C NMR (126 MHz, DMSO-d_6) δ 9.90, 21.00, 27.91, 30.37, 35.65, 67.45, 104.95, 114.26, 117.99, 119.02, 119.54, 119.72, 121.59, 124.75, 125.04, 125.26, 126.23, 126.39, 127.42, 127.79, 134.04,137.36, 145.00, 154.15, 163.26; m/z (ES$^+$), $[M+H]^+$ calcd for $C_{56}H_{52}N_6O_6S$, 937.37; found 937.1.

4: ^1H NMR (300 MHz, CD_3OD) δ 2.06 (s, 3H), 2.15 (s, 3H), 2.24 - 2.43 (m, 2H), 3.41- 3.60 (m, 9H), 3.85 (s, 3H), 4.21 (t, *J* = 1.0 Hz, 2H), 5.54 (s, 1H), 6.78 (d, *J* = 1.0 Hz, 1H), 7.00 - 7.12 (m, 2H), 7.30 (t, *J* = 1.0 Hz, 1H), 7.38 (d, *J* = 1.0 Hz, 1H), 7.41 - 7.53 (m, 2H), 7.67 - 7.74 (m, 1H), 7.74 - 7.84 (m, 1H), 8.13 - 8.41 (m, 1H); ^{13}C NMR (126 MHz, CD_3OD) 10.26, 12.68, 22.46, 26.06, 27.37, 31.79, 35.77, 36.40, 68.60, 105.59, 107.25, 116.53, 118.56, 120.92, 120.99, 121.13, 122.99, 125.42, 125.65, 126.02, 127.04, 127.07, 127.27, 128.38, 128.45, 129.72, 136.03, 137.09, 140.31, 142.20, 147.37, 147.45, 156.03, 165.37; m/z (ES$^+$), HRMS calcd for $C_{34}H_{36}N_5O_3S$ $[M+H]^+$, 594.2533; found 594.2519.

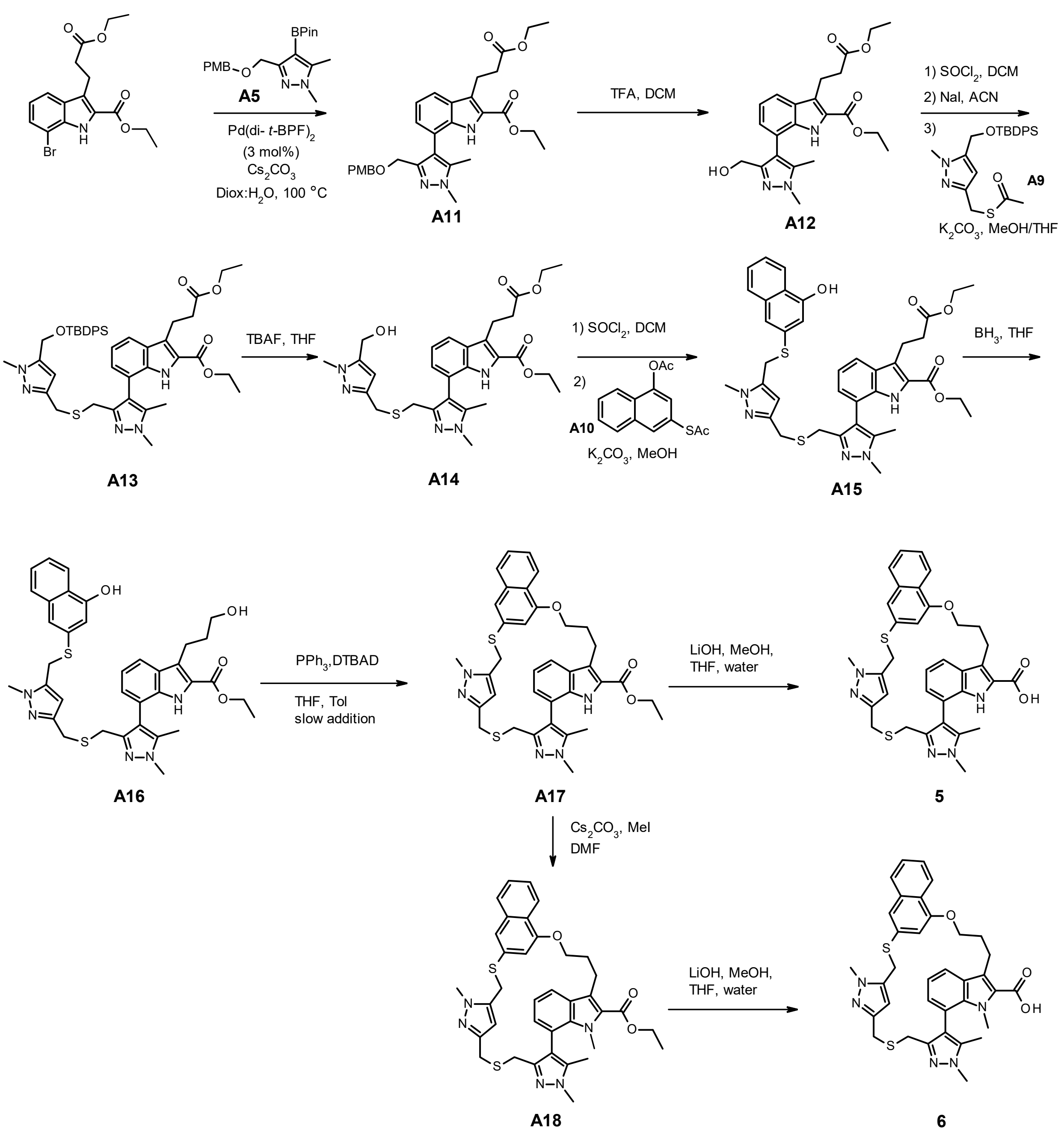

Supplementary Figure 15: Synthetic scheme for compounds **5** and **6**

4-Bromo-3-(((4-methoxybenzyl)oxy)methyl)-1,5-dimethyl-1H-pyrazole (**A4**)

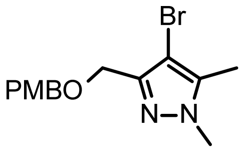

N,N-dimethylformamide (DMF, 112 mL) was added to (4-bromo-1,5-dimethyl-1H-pyrazol-

3-yl)methanol **A1** (3.74 g, 18.3 mmol) and the solution was cooled to 0 °C. NaH (0.84 g, 21.0 mmol) (60% in mineral oil) was added. The mixture was stirred at 0 °C for 10 min, allowed to warm to RT and stirred for 20 min, resulting in a white suspension. 1-(Chloromethyl)-4-methoxybenzene (2.72 mL, 20.1 mmol) and KI (0.30 g, 1.83 mmol) were added and the mixture was stirred for 1 h and concentrated to dryness. Water (50 mL) was added and the mixture was extracted with EtOAc (3 x 20 mL). The combined organic phases were dried over Na_2SO_4, filtered and concentrated to dryness. The residue was purified by silica gel column chromatography (hexanes/EtOAc) to give 4-bromo-3-(((4-methoxybenzyl)oxy)methyl)-1,5-dimethyl-1H-pyrazole **A4** (5.69 g, 96.0%).

^{1}H NMR (400 MHz, CHLOROFORM-*d*) δ 2.26 (s, 3H), 3.80 (s, 3H), 3.81 (s, 3H), 4.47 (s, 2H), 4.53 (s, 2H), 6.85 (d, *J* = 8.7 Hz, 2H), 7.33 (d, *J* = 8.7 Hz, 2H); ^{13}C NMR (126 MHz, CHLOROFORM-*d*) δ 10.17, 37.18, 55.25, 63.65, 71.96, 93.95, 113.67, 129.55, 130.27, 137.81, 145.85, 159.13; m/z (ES$^+$), [M+H]$^+$ calcd for $C_{14}H_{17}BrN_2O_2$, 325.04; found 325.0.

3-(((4-Methoxybenzyl)oxy)methyl)-1,5-dimethyl-4-(4,4,5,5-tetramethyl-1,3,2-dioxaborolan-2-yl)-1H-pyrazole (**A5**)

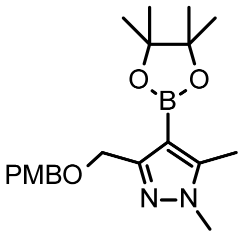

THF (83 mL) was added to 4-bromo-3-(((4-methoxybenzyl)oxy)methyl)-1,5-dimethyl-1H-pyrazole **A4** (3.02 g, 9.29 mmol) and the resulting clear solution was cooled to -78 °C. Butyllithium (6.96 mL, 11.1 mmol) (1.6 M in hexane) was added at -78 °C under Ar. The mixture was stirred at -78 °C for 50 min. 2-Isopropoxy-4,4,5,5-tetramethyl-1,3,2-dioxaborolane (2.65 mL, 13.0 mmol) was added. The acetone/dry ice bath was removed. The mixture was slowly warmed to RT and stirred for 4 h. The mixture was concentrated to dryness and EtOAc (200 mL) was added. The resulting suspension was filtered through a pad of diatomaceous earth, washed with EtOAc (50 mL). The filtrate was concentrated to dryness and the residue was purified by silica gel column chromatography (hexanes/EtOAc) to give 3-(((4-methoxybenzyl)oxy)methyl)-1,5-dimethyl-4-(4,4,5,5-tetramethyl-1,3,2-dioxaborolan-2-yl)-1H-pyrazole **A5** (2.76 g, 80.0%).

^{1}H NMR (400 MHz, CHLOROFORM-*d*) δ 1.28 (s, 12H), 2.40 (s, 3H), 3.76 (s, 3H), 3.80 (s, 3H), 4.57 (s, 2H), 4.61 (s, 2H), 6.86 (d, *J* = 8.7 Hz, 2H), 7.33 (d, *J* = 8.7 Hz, 2H); ^{13}C NMR (75 MHz,

CHLOROFORM-*d*) δ 11.22, 24.82, 35.64, 55.18, 65.30, 72.14, 82.53, 113.47, 129.40, 131.00, 147.18, 154.24, 158.89; m/z (ES$^+$), [M+H]$^+$ calcd for $C_{20}H_{29}BN_2O_4$, 373.22; found 373.4.

Ethyl 5-(((*tert*-butyldiphenylsilyl)oxy)methyl)-1H-pyrazole-3-carboxylate (**A6**)

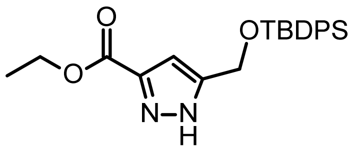

1-Hydroxypropan-2-one (34.9 mL, 463 mmol) was dissolved in anhydrous DMF (150 mL) under Ar. Imidazole (34.1 g, 501 mmol) and 4-dimethylaminopyridine (DMAP, 2.37 g, 19.3 mmol) were added and the solution was cooled to 0 °C. *Tert*-butyldiphenylsilyl chloride (TBDPSCl, 100 mL, 386 mmol) was added slowly. The mixture was stirred at 0 °C for 15 min, then at RT for 18 h under Ar. Water (1 L) was added and the aqueous phase was extracted with hexanes (4 x 200 mL). The combined organic phases were washed with brine, dried over Na_2SO_4, filtered and concentrated to give crude 1-((*tert*-butyldiphenylsilyl)oxy)propan-2-one (120 g, 100%). This material was used without further purification.

THF (1.50 L) was added to potassium *tert*-butoxide (69.0 g, 570 mmol) and the solution was cooled to 0 °C. Diethyl oxalate (78.1 g, 570 mmol) was added slowly, maintaining the temperature below 0 °C. The solution was stirred for 30 min at 0 °C. 1-((*tert*-Butyldiphenylsilyl)oxy)propan-2-one (150 g, 480 mmol) was added slowly. The reaction mixture was stirred at 0 °C for 1 h, and then EtOAc (300 mL) was added. The resulting mixture was acidified with 1 N HCl to pH=2 to 3. The phases were separated and the aqueous phase was extracted with EtOAc (4 x 300 mL). The combined organic phases were washed with brine, dried over Na_2SO_4, filtered and concentrated to dryness to give crude ethyl 5-((*tert*-butyldiphenylsilyl)oxy)-2-hydroxy-4-oxopent-2-enoate (160 g, 80.0%) which was used without purification.

Ethyl 5-((*tert*-butyldiphenylsilyl)oxy)-2-hydroxy-4-oxopent-2-enoate (350.0 g, 848.0 mmol) was dissolved in ethanol (80.5 mL). The solution was cooled to 0°C and hydrazine monohydrate (53.2 g, 848 mmol, 80 weight percent (wt%)) was added at 0 °C. The mixture was stirred at 80 °C for 2 h. After completion, the mixture was cooled to 60 °C and the solvent was removed under reduced pressure. The residue was diluted with EtOAc (161 mL), and washed with saturated NH_4Cl (64.6 mL). The aqueous layer was extracted with EtOAc (2 x 64.6 mL). The combined organic layers were dried over Na_2SO_4, filtered and evaporated to afford crude product. The crude product was purified by flash silica chromatography (0 to 20% EtOAc in PE)

to give ethyl 5-(((*tert*-butyldiphenylsilyl)oxy)methyl)-1H-pyrazole-3-carboxylate **A6** (176 g, 60.0%).

^{1}H NMR (300 MHz, DMSO-d_6), (reported as a mixture of tautormers) δ 1.00 (s, 9H), 1.24 - 1.31 (m, 3H), 4.08 - 4.43 (m, 2H), 4.71 (s, 1H$_{minor}$), 4.74 (s, 1H$_{minor}$), 6.54 (s, 1H$_{major}$), 6.71 (s, 1H$_{minor}$), 7.42 - 7.50 (m, 6H), 7.62 - 7.65 (m. 4H), 13. 48 (s, 1H$_{major}$) 13.81 (s, 1H$_{minor}$); ^{13}C NMR (75 MHz, DMSO-d_6),(reported as a mixture of tautormers) δ 14.07, 14.16, 18.70, 18.73, 26.49, 26.52, 56.83, 59.77, 59.89, 60.64, 105.80, 107.06, 127.84, 127.90, 129.84, 129.96, 132.45, 132.84, 133.85, 135.01, 143.07, 152.00, 158.89, 162.00; m/z (ES$^-$), [M-H]$^-$ calcd for $C_{23}H_{28}N_2O_3Si$, 407.18; found 407.4.

Ethyl 5-(((*tert*-butyldiphenylsilyl)oxy)methyl)-1-methyl-1H-pyrazole-3-carboxylate (**A7**)

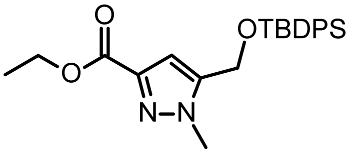

Ethyl 5-(((*tert*-butyldiphenylsilyl)oxy)methyl)-1H-pyrazole-3-carboxylate **A6** (175 g, 428 mmol) was dissolved in anhydrous THF (1750 mL). The solution was cooled to 0 °C and sodium hexamethyldisilazane (NaHMDS, 238 mL, 476 mmol, 2 M in THF) was added at 0 °C under Ar. The resulting mixture was stirred at 0 °C for 10 min then RT for 30 min. Iodomethane (91.0 g, 642 mmol) was added and the mixture was stirred for 2 h. After completion of reaction, the mixture was concentrated to dryness. EtOAc (3500 mL) was added and the resulting solution was washed with saturated aqueous (sat. aq.) NH_4Cl solution (1750 mL). The aqueous phase was extracted with EtOAc (2 x 3500 mL). The combined organic phases were dried over Na_2SO_4, filtered and evaporated to afford ethyl 5-(((*tert*-butyldiphenylsilyl)oxy)methyl)-1-methyl-1H-pyrazole-3-carboxylate **A7** (160 g, 88.0%) which was used without purification.

^{1}H NMR (300 MHz, CHLOROFORM-*d*) δ 1.05 (s, 9H), 1.41 (t, J = 7.2 Hz, 3H), 3.95 (s, 3H), 4.42 (q, J = 7.2 Hz, 2H), 4.68 (s, 2H), 6.56 (s, 1H), 7.37 - 7.50 (m, 6H), 7.61 - 7.69 (m, 4H); ^{13}C NMR (300 MHz, CHLOROFORM-d) δ 14.33, 19.11, 26.62, 37.53, 56.67, 60.74, 108.40, 127.83, 129.98, 132.51, 135.44, 142.04, 142.44, 162.28; m/z (ES$^+$), [M+H]$^+$ calcd for $C_{24}H_{30}N_2O_3Si$, 423.20; found 423.2.

(5-(((*tert*-Butyldiphenylsilyl)oxy)methyl)-1-methyl-1H-pyrazol-3-yl)methanol (**A8**)

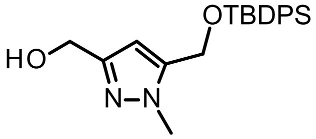

THF (800 mL) was added to ethyl 5-(((*tert*-butyldiphenylsilyl)oxy)methyl)-1-methyl-1H-pyrazole-3-carboxylate **A7** (160 g, 378 mmol) to give an orange solution. The solution was cooled to 0 °C and lithium aluminium hydride (LAH, 189 mL, 47.3 mmol) (2.0 M in THF) was added dropwise, maintaining the temperature below 0 °C. The resulting mixture was stirred at 0 °C for 1 h. The mixture was diluted with diethyl ether (1600 mL) and water (14.4 mL) was added dropwise below 0 °C, followed by 15% aq. NaOH solution (14.4 mL), and water (43 mL). The resulting mixture was stirred at RT for 10 min. Anhydrous Na_2SO_4 was added and the suspension was stirred for 15 min. The mass was filtered through a pad of diatomaceous earth and washed with diethyl ether. The filtrate was concentrated to obtain (5-(((*tert*-butyldiphenylsilyl)oxy)methyl)-1-methyl-1H-pyrazol-3-yl)methanol **A8** (140 g, 97.0%).
^{1}H NMR (300 MHz, CHLOROFORM-*d*) δ 1.06 (s, 9H), 3.85 (s, 3H), 4.62 (s, 2H), 4.64 (s, 2H), 6.02 (s, 1H), 7.35 - 7.53 (m, 6H), 7.62 - 7.72 (m, 4H); ^{13}C NMR (75 MHz, CHLOROFORM-*d*) δ 19.16, 26.68, 36.49, 56.92, 58.67, 103.93, 127.78, 129.90, 132.79, 135.51, 142.01, 150.74; m/z (ES$^+$), [M+H]$^+$ calcd for $C_{22}H_{28}N_2O_2Si$, 381.19; found 381.2.

S-((5-(((*tert*-Butyldiphenylsilyl)oxy)methyl)-1-methyl-1H-pyrazol-3-yl)methyl) ethanethioate (**A9**)

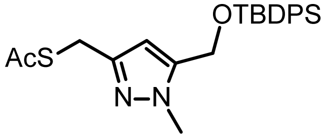

(5-(((*tert*-Butyldiphenylsilyl)oxy)methyl)-1-methyl-1H-pyrazol-3-yl)methanol **A8** (380 g, 998 mmol) was dissolved in DCM (4560 mL). The solution was cooled to 0 °C and thionyl chloride (87.4 mL, 1200 mmol) was added very slowly at 0 °C. The reaction mixture was allowed to warm to RT and stirred for 1 h. In another flask sat. aq. sodium bicarbonate solution (6330 mL) was cooled to 0 °C. The reaction mixture was slowly added to the sodium bicarbonate solution with stirring. The biphasic mixture was stirred until it stopped bubbling. The phases were separated. The organic phase was washed with brine, dried over anhydrous Na_2SO_4, filtered and concentrated to give 5-(((*tert*-butyldiphenylsilyl)oxy)methyl)-3-(chloromethyl)-1-methyl-1H-pyrazole (390 g).

5-(((*tert*-Butyldiphenylsilyl)oxy)methyl)-3-(chloromethyl)-1-methyl-1H-pyrazole (390 g, 977 mmol) was dissolved in acetonitrile (4130 mL). Potassium thioacetate (233 g, 1950 mmol)

and sodium iodide (149 g, 9.42 mmol) were added. The reaction mixture was stirred for 12 h at RT. After completion of reaction, the mixture was filtered through a bed of diatomaceous earth and washed with dichloromethane. The filtrate was concentrated under reduced pressure and the residue was purified by silica gel chromatography (0 to 20% EtOAc in hexane) to obtain S-((5-(((*tert*-butyldiphenylsilyl)oxy)methyl)-1-methyl-1H-pyrazol-3-yl)methyl)ethanethioate **A9** (309 g, 72.0%).

^{1}H NMR (400 MHz, CHLOROFORM-*d*) δ 1.04 (s, 9H), 2.34 (s, 3H), 3.80 (s, 3H), 4.08 (s, 2H), 4.60 (s, 2 H), 5.92 (s, 1H), 7.35 - 7.5 (m, 6H), 7.58 - 7.69 (m, 4H); ^{13}C NMR (75 MHz, CHLOROFORM-*d*) δ 19.04, 26.26, 26.59, 30.10, 36.48, 56.76, 104.97, 127.67, 129.79, 132.65, 135.40, 141.88, 146.61, 194.81; m/z (ES^{+}), [M+H]$^{+}$ calcd for $C_{24}H_{30}N_2O_2SSi$, 439.17; found 439.2.

3-(Acetylthio)naphthalen-1-yl acetate (**A10**)

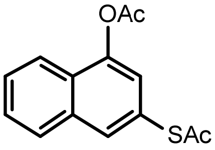

I_2 (38.7 g, 152 mmol) was added in one portion to a mixture of sodium 4-hydroxynaphthalene-2-sulfonate (75.0 g, 305 mmol), Ph_3P (320 g, 1220 mmol) and 18-crown-6 (24.2 g, 91.4 mmol) in toluene (750 mL) at 20 °C under nitrogen. The resulting mixture was stirred at 100 °C for 17 h. 1,4-Dioxane (150 mL) and water (75 mL) were added and the mixture was stirred at 100 °C for another 1 h. Na_2SO_4 was added. The solids were removed by filtration and the filtrate was partially concentrated under vacuum to afford 3-mercaptonaphthalen-1-ol (360 g, 14 wt% in toluene). The product was used without further purification.

Ac_2O (162 mL, 1720 mmol) was added dropwise to a mixture of DMAP (3.49 g, 28.6 mmol), 3-mercaptonaphthalen-1-ol (360 g, 286 mmol, 14 wt% in toluene) and Et_3N (80 mL, 572 mmol) in DCM (1000 mL) at 0 °C over a period of 10 min under nitrogen. The resulting mixture was stirred at 0 °C for 30 min. The reaction mixture was diluted with DCM (200 mL), and washed sequentially with water (4 x 750 mL) and saturated brine (500 mL). The organic layer was dried over Na_2SO_4, filtered and concentrated to dryness. The residue was purified by silica gel column chromatography (PE/EtOAc) to give 3-(acetylthio)naphthalen-1-yl acetate **A10** (40.0 g, 50.0% over 2 steps).

^{1}H NMR (400 MHz, CHLOROFORM-*d*) δ 2.48 (s, 3H), 2.49 (s, 3H), 7.34 (d, *J* = 1.5 Hz, 1H), 7.55 - 7.62 (m, 2H), 7.88 - 7.92 (m, 3H); ^{13}C NMR (75 MHz, CHLOROFORM-*d*) δ 20.76, 30.08, 121.15,

122.84, 124.93, 126.79, 127.05, 127.48, 127.98, 131.99, 134.30, 146.63, 168.92, 193.49; m/z (ES^+), $[M+H]^+$ calcd for $C_{14}H_{12}O_3S$, 261.05; found 261.2.

Ethyl 3-(3-ethoxy-3-oxopropyl)-7-(3-(((4-methoxybenzyl)oxy)methyl)-1,5-dimethyl-1H-pyrazol-4-yl)-1H-indole-2-carboxylate (**A11**)

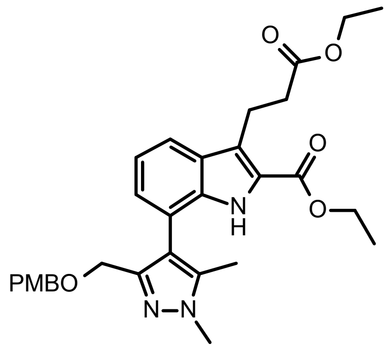

3-((4-Methoxybenzyl)oxy)methyl)-1,5-dimethyl-4-(4,4,5,5-tetramethyl-1,3,2-dioxaborolan-2-yl)-1H-pyrazole **A5** (7.28 g, 19.6 mmol) was dissolved in a mixture of 1,4-dioxane and water (4:1, 136 mL). Cs_2CO_3 (10.6 g, 32.6 mmol), ethyl 7-bromo-3-(3-ethoxy-3-oxopropyl)-1H-indole-2-carboxylate (6.00 g, 16.3 mmol) and dichloro[1,1'-bis(di-*tert*-butylphosphino)ferrocene] palladium(II) (0.33 g, 0.49 mmol) were added. The mixture was degassed and filled with N_2 three times. The resulting brown clear mixture was placed in an oil bath preheated to 100 °C. The mixture was stirred at 100 °C for 30 min. The mixture was cooled to RT and concentrated to dryness. EtOAc (100 mL) and water (50 mL) were added. The layers were separated and the aqueous phase was extracted with EtOAc (3 x 20 mL). The combined organic phases were dried over Na_2SO_4, filtered and concentrated. The residue was purified by silica gel column chromatography (hexanes/EtOAc) to give ethyl 3-(3-ethoxy-3-oxopropyl)-7-(3-(((4-methoxybenzyl)oxy)methyl)-1,5-dimethyl-1H-pyrazol-4-yl)-1H-indole-2-carboxylate **A11** (8.14 g, 93.6%).

^1H NMR (300 MHz, CHLOROFORM-*d*) δ 1.20 (t, J = 7.2 Hz, 3H), 1.33 (t, J = 7.1 Hz, 3H), 2.18 (s, 3H), 2.66 - 2.79 (m, 2H), 3.42 - 3.52 (m, 2H), 3.75 (s, 3H), 3.86 (s, 3H), 4.12 (q, J = 7.2 Hz, 2H), 4.22 - 4.35 (m, 4H), 4.54 (s, 2H), 6.79 (d, J = 7.8 Hz, 2H), 7.07 - 7.20 (m, 4H), 7.70 (d, J = 7.7 Hz, 1H), 9.75 (s, 1H); ^{13}C NMR (75 MHz, CHLOROFORM-*d*) δ 10.06, 13.99, 14.21, 20.32, 35.17, 36.37, 54.98, 60.09, 60.41, 63.21, 72.02, 113.49, 115.81, 117.39, 119.41, 119.99, 122.88, 123.69, 126.89, 127.74, 129.48, 129.53, 135.37, 137.59, 145.50, 158.98, 161.66, 173.03; m/z (ES+), $[M+H]^+$ calcd for $C_{30}H_{35}N_3O_6$, 534.25; found 534.2.

Ethyl 3-(3-ethoxy-3-oxopropyl)-7-(3-(hydroxymethyl)-1,5-dimethyl-1H-pyrazol-4-yl)-1H-indole-2-carboxylate (**A12**)

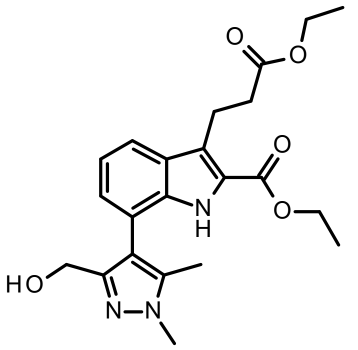

Ethyl 3-(3-ethoxy-3-oxopropyl)-7-(3-(((4-methoxybenzyl)oxy)methyl)-1,5-dimethyl-1H-pyrazol-4-yl)-1H-indole-2-carboxylate **A11** (3.60 g, 5.80 mmol) was dissolved in DCM (5 mL) and TFA (4.47 mL, 58.0 mmol) was added at 0 °C. The ice bath was removed and the reaction was continued at RT for 4 h. The mixture was concentrated to dryness. DCM (100 mL) and sat. aq. $NaHCO_3$ (50 mL) were added to the residue. The biphase was stirred at RT for 4h and separated. The organic phase was washed with sat. aq. $NaHCO_3$ (2 x 50 mL), dried over Na_2SO_4, filtered and concentrated. The residue was purified by silica gel column chromatography (DCM/EtOAc then 10% MeOH in DCM) to give ethyl 3-(3-ethoxy-3-oxopropyl)-7-(3-(hydroxymethyl)-1,5-dimethyl-1H-pyrazol-4-yl)-1H-indole-2-carboxylate **A12** (2.40 g, >98%).

^{1}H NMR (300 MHz, CHLOROFORM-*d*) δ 1.22 (t, 3H), 1.38 (t, *J* = 7.1 Hz, 3H), 2.23 (s, 3H), 2.66 - 2.76 (m, 2H), 3.40 - 3.50 (m, 2H), 3.88 (s, 3H), 4.13 (q, *J* = 7.1 Hz, 2H), 4.38 (q, *J* = 7.2 Hz, 2H), 4.57 (br s, 2H), 7.07 - 7.22 (m, 2H), 7.70 (d, *J* = 7.7 Hz, 1H), 10.42 (s, 1H); ^{13}C NMR (75 MHz, CHLOROFORM-*d*) δ 10.30, 14.14, 14.29, 20.49, 35.37, 36.33, 56.26, 60.27, 60.60, 115.16, 117.29, 119.64, 120.05, 122.62, 124.24, 126.74, 128.12, 135.51, 138.01, 148.34, 162.13, 173.26; m/z (ES$^+$), $[M+H]^+$ calcd for $C_{22}H_{27}N_3O_5$, 414.19; found 414.2.

Ethyl 7-(3-((((5-(((*tert*-butyldiphenylsilyl)oxy)methyl)-1-methyl-1H-pyrazol-3-yl)methyl)thio)methyl)-1,5-dimethyl-1H-pyrazol-4-yl)-3-(3-ethoxy-3-oxopropyl)-1H-indole-2-carboxylate (**A13**)

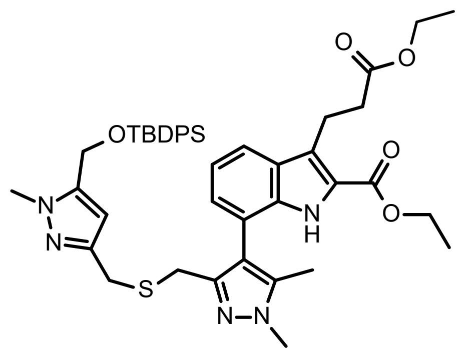

Ethyl 3-(3-ethoxy-3-oxopropyl)-7-(3-(hydroxymethyl)-1,5-dimethyl-1H-pyrazol-4-yl)-1H-indole-2-carboxylate **A12** (0.98 g, 2.36 mmol) was dissolved in anhydrous DCM (5 mL). The solution was cooled to 0 °C. Thionyl chloride (0.19 mL, 2.60 mmol) was added. The mixture was stirred at 0 °C for 1 h, diluted with DCM (10 mL) and then quenched with sat. $NaHCO_3$ solution (10 mL) at 0 °C. The biphases were separated. The organic phase was dried over Na_2SO_4, filtered and concentrated to dryness to give crude ethyl 7-(3-(chloromethyl)-1,5-dimethyl-1H-pyrazol-4-yl)-3-(3-ethoxy-3-oxopropyl)-1H-indole-2-carboxylate. This crude intermediate was dissolved in THF (6 mL). Sodium iodide (1.77 g, 11.8 mmol) was added. The suspension was stirred at RT for 2 h. After the solvent was removed under reduced pressure, EtOAc (20 mL) was added to the residue. The resulting solution was washed with water (10 mL), brine, dried over Na_2SO_4, filtered and concentrated to dryness. MeOH (6 mL) was added the residue, followed by addition of S-((5-(((*tert*-butyldiphenylsilyl)oxy)methyl)-1-methyl-1H-pyrazol-3-yl)methyl)ethanethioate **A9** (1.04 g, 2.36 mmol) in THF (6 mL). The mixture was degassed, filled with N_2 and cooled to 0 °C. Ph_3P (0.06 g, 0.24 mmol) and powder K_2CO_3 (0.33 g, 2.36 mmol) were added. The suspension was stirred at 0 °C for 30 min, then RT for 1h under N_2.
The mixture was concentrated to dryness. EtOAc (50 mL) was added to the residue. The resulting solution was washed with water, brine, dried over Na_2SO_4, filtered and concentrated to dryness. The crude product was purified by silica gel column chromatography (hexanes/EtOAc) to give ethyl 7-(3-((((5-(((*tert*-butyldiphenylsilyl)oxy)methyl)-1-methyl-1H-pyrazol-3-yl)methyl)thio)methyl)-1,5-dimethyl-1H-pyrazol-4-yl)-3-(3-ethoxy-3-oxopropyl)-1H-indole-2-carboxylate **A13** (1.20 g, 64.2%).
^{1}H NMR (300 MHz, CHLOROFORM-*d*) δ 1.04 (s, 9H), 1.21 (t, *J* = 7.0 Hz, 3H), 1.34 (t, *J* = 7.2 Hz, 3H), 2.12 (s, 3H), 2.69 (t, *J* = 8.1 Hz, 2H), 3.35 - 3.63 (m, 4H), 3.68 - 3.77 (m, 5H), 3.83 (s, 3H), 4.12 (q, *J* = 7.2 Hz, 2H), 4.35 (q, *J* = 7.0 Hz, 2H), 4.58 (s, 2H), 5.92 (s, 1H), 7.07 - 7.21 (m, 2H), 7.31 - 7.52 (m, 6H), 7.59 - 7.74 (m, 5H), 9.23 (s, 1H); ^{13}C NMR (75 MHz, CHLOROFORM-*d*) δ 10.23, 14.19, 14.32, 19.21, 20.50, 26.74, 27.39, 29.38, 35.32, 36.46, 36.49, 56.94, 60.31,

60.70, 105.03, 114.77, 117.37, 119.74, 120.33, 123.03, 123.77, 127.53, 127.77, 127.83, 129.93, 132.83, 135.42, 135.56, 137.54, 141.75, 146.30, 147.89, 161.93, 173.23; m/z (ES$^+$), $[M+H]^+$ calcd for $C_{44}H_{53}N_5O_5SSi$, 792.35; found 792.2.

Ethyl 3-(3-ethoxy-3-oxopropyl)-7-(3-((((5-(hydroxymethyl)-1-methyl-1H-pyrazol-3-yl)methyl)thio)methyl)-1,5-dimethyl-1H-pyrazol-4-yl)-1H-indole-2-carboxylate (**A14**)

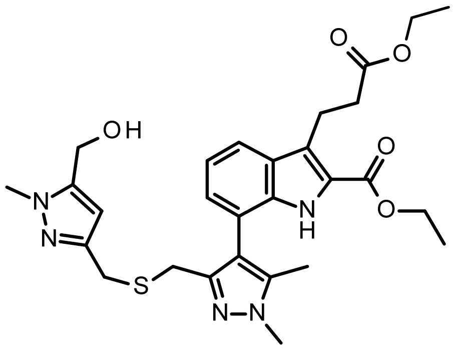

Ethyl 7-(3-((((5-(((*tert*-butyldiphenylsilyl)oxy)methyl)-1-methyl-1H-pyrazol-3-yl)methyl)thio)methyl)-1,5-dimethyl-1H-pyrazol-4-yl)-3-(3-ethoxy-3-oxopropyl)-1H-indole-2-carboxylate **A13** (1.20 g, 1.51 mmol) was dissolved in THF (13.6 mL). Tetrabutylammonium fluoride (TBAF, 1.51 mL, 1.51 mmol) (1M in THF) was added. The mixture was stirred at RT for 2 h and then concentrated to dryness. EtOAc (50 mL) was added to the residue. The resulting solution was washed with water (10 mL), brine (10 mL), dried over Na_2SO_4, filtered and concentrated to dryness. The crude product was purified by silica gel chromatography (hexanes/EtOAc) to give ethyl 3-(3-ethoxy-3-oxopropyl)-7-(3-((((5-(hydroxymethyl)-1-methyl-1H-pyrazol-3-yl)methyl)thio)methyl)-1,5-dimethyl-1H-pyrazol-4-yl)-1H-indole-2-carboxylate **A14** (0.710 g, 85.0%).

^{1}H NMR (500 MHz, CHLOROFORM-*d*) δ 1.20 (t, *J* = 7.2 Hz, 3H), 1.36 (t, *J* = 7.2 Hz, 3H), 2.13 (s, 3H), 2.70 (t, *J* = 7.9 Hz, 2H), 3.40 - 3.50 (m, 2H), 3.53 - 3.69 (m, 4H), 3.72 (s, 3H), 3.84 (s, 3H), 4.11 (q, *J* = 7.2 Hz, 2H), 4.35 (q, *J* = 7.2 Hz, 2H), 4.53 (s, 2H), 5.92 (s, 1H), 7.10 (d, *J* = 7.9 Hz, 1H), 7.15 - 7.22 (m, 1H), 7.70 (d, *J* = 7.9 Hz, 1H), 9.28 (br s, 1H); ^{13}C NMR (126 MHz, CHLOROFORM-*d*) δ 10.19, 14.16, 14.30, 20.45, 27.48, 29.27, 35.27, 36.22, 36.44, 55.41, 60.37, 60.83, 104.85, 114.90, 117.31, 119.78, 120.34, 123.07, 123.69, 127.53, 127.73, 135.46, 137.74, 142.13, 145.96, 148.25, 162.18, 173.23; m/z (ES$^+$), $[M+H]^+$ calcd for $C_{28}H_{35}N_5O_5S$, 554.23; found 534.2.

Ethyl 3-(3-ethoxy-3-oxopropyl)-7-(3-((((5-(((4-hydroxynaphthalen-2-yl)thio)methyl)-1-methyl-1H-pyrazol-3-yl)methyl)thio)methyl)-1,5-dimethyl-1H-pyrazol-4-yl)-1H-indole-2-carboxylate (**A15**)

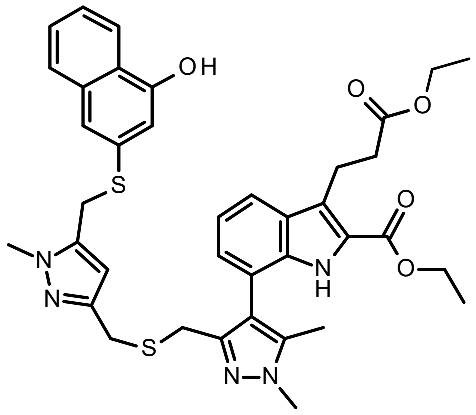

Ethyl 3-(3-ethoxy-3-oxopropyl)-7-(3-((((5-(hydroxymethyl)-1-methyl-1H-pyrazol-3-yl)methyl)thio)methyl)-1,5-dimethyl-1H-pyrazol-4-yl)-1H-indole-2-carboxylate **A14** (88.0 mg, 0.16 mmol) was dissolved in DCM (1 mL) and cooled to 0 °C. Thionyl chloride (0.024 mL, 0.32 mmol) was added. After stirring at 1 h at 0 °C, the mixture was washed with sat. Na_2CO_3 solution (1 mL), dried over Na_2SO_4, filtered and concentrated to dryness to give ethyl 7-(3-((((5-(chloromethyl)-1-methyl-1H-pyrazol-3-yl)methyl)thio)methyl)-1,5-dimethyl-1H-pyrazol-4-yl)-3-(3-ethoxy-3-oxopropyl)-1H-indole-2-carboxylate which was used without purification.
3-(Acetylthio)naphthalen-1-yl acetate **A10** (41.0 mg, 0.16 mmol) was added to a solution of ethyl 7-(3-((((5-(chloromethyl)-1-methyl-1H-pyrazol-3-yl)methyl)thio)methyl)-1,5-dimethyl-1H-pyrazol-4-yl)-3-(3-ethoxy-3-oxopropyl)-1H-indole-2-carboxylate in anhydrous methanol (1 mL). The mixture was degassed and filled with N_2. Triphenylphosphine (2.08 mg, 7.95 µmol) was added, followed by addition of powder K_2CO_3 (44.0 mg, 0.32 mmol) at RT. The suspension was stirred at RT under N_2 for 30 min. The mixture was concentrated and EtOAc (10 mL) was added. The organic phase was washed with water, brine, dried over Na_2SO_4, filtered and concentrated to dryness. The crude product was purified by silica gel chromatography (hexanes/EtOAc) to give ethyl 3-(3-ethoxy-3-oxopropyl)-7-(3-((((5-(((4-hydroxynaphthalen-2-yl)thio)methyl)-1-methyl-1H-pyrazol-3-yl)methyl)thio)methyl)-1,5-dimethyl-1H-pyrazol-4-yl)-1H-indole-2-carboxylate **A15** (90.0 mg, 80.0%) as a dry film.
^{1}H NMR (500 MHz, CHLOROFORM-*d*) δ 1.24 (t, *J* = 7.2 Hz, 3H), 1.31 (t, *J* = 7.2 Hz, 3H), 2.16 (s, 3H), 2.74 (t, *J* = 8.0 Hz, 2H), 3.40 - 3.55 (m, 4H), 3.64 - 3.73 (m, 5H), 3.80 - 3.99 (m, 5H), 4.16 (q, *J* = 7.2 Hz, 2H), 4.34 (q, *J* = 7.2 Hz, 2H), 5.75 (s, 1H), 6.35 (s, 1H), 7.12 (d, *J* = 6.7 Hz, 1H), 7.21 (t, *J* = 7.6 Hz, 1H), 7.42 - 7.55 (m, 2H), 7.64 (s, 1H), 7.68 - 7.82 (m, 2H), 8.24 - 8.41 (m, 1H), 10.53 (s, 1H); ^{13}C NMR (126 MHz, CHLOROFORM-*d*) δ 10.18, 14.19, 14.24, 20.59,

27.39, 29.39, 31.14, 35.37, 35.95, 36.03, 60.38, 60.60, 106.46, 114.10, 116.59, 116.87, 120.12, 120.25, 122.71, 123.02, 124.30, 125.03, 125.17, 125.49, 126.74, 127.18, 127.64, 128.00, 130.32, 134.72, 136.02, 138.32, 139.95, 145.89, 148.71, 153.24, 162.14, 173.39; m/z (ES^+), $[M+H]^+$ calcd for $C_{38}H_{41}N_5O_5S_2$, 712.25; found 712.1.

Ethyl 7-(3-((((5-(((4-hydroxynaphthalen-2-yl)thio)methyl)-1-methyl-1H-pyrazol-3-yl)methyl)thio)methyl)-1,5-dimethyl-1H-pyrazol-4-yl)-3-(3-hydroxypropyl)-1H-indole-2-carboxylate (**A16**)

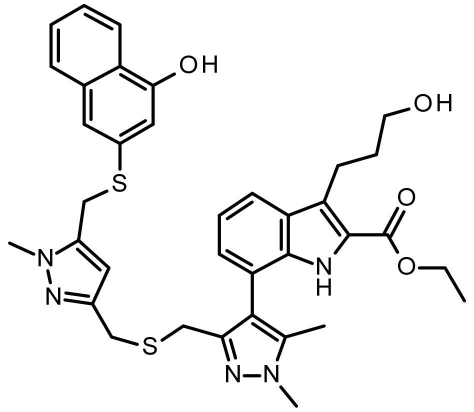

Ethyl 3-(3-ethoxy-3-oxopropyl)-7-(3-((((5-(((4-hydroxynaphthalen-2-yl)thio)methyl)-1-methyl-1H-pyrazol-3-yl)methyl)thio)methyl)-1,5-dimethyl-1H-pyrazol-4-yl)-1H-indole-2-carboxylate **A15** (626 mg, 0.88 mmol) was dissolved into THF (6 mL). The resulting solution was degassed, filled with N_2 and cooled to 0 °C. Borane tetrahydrofuran complex (4.84 mL, 4.84 mmol) (1M in THF) was added slowly. Upon completion of addition, the ice bath was removed and the resulting mixture was stirred at RT for 5 h. The solvent was removed under vacuum to give a white solid. THF (30 mL) and MeOH (10 mL) were add to this solid, followed by addition of 2 grams of Amberlite IRA743 free base and 1 mL of 10% HCl solution at RT. The resulting suspension was stirred at RT for 3 days. The solids were filtered off, washed with DCM (10 mL) and MeOH (10 mL). The filtrate was concentrated to dryness. The residue was purified by silica gel chromatography (50% to100% EtOAc in hexanes, then 20% MeOH in EtOAc) to give ethyl 7-(3-((((5-(((4-hydroxynaphthalen-2-yl)thio)methyl)-1-methyl-1H-pyrazol-3-yl)methyl)thio)methyl)-1,5-dimethyl-1H-pyrazol-4-yl)-3-(3-hydroxypropyl)-1H-indole-2-carboxylate **A16** (543 mg, 92.0%).

^1H NMR (500 MHz, DMSO-d_6) δ 1.28 (t, J = 7.1 Hz, 3H), 1.69 - 1.83 (m, 2H), 2.02 (s, 3H), 3.04 - 3.07 (m, 2H), 3.41 - 3.53 (m, 6H), 3.68 (s, 3H), 3.73 (s, 3H), 4.21 - 4.32 (m, 4H), 4.44 (t, J = 5.1 Hz, 1H), 5.95 (s, 1H), 6.78 (s, 1H), 7.04 - 7.15 (m, 2H), 7.30 (s, 1H), 7.37 (t, J = 7.3 Hz, 1H),

7.43 (t, J = 7.3 Hz, 1H), 7.63 (d, J = 7.6 Hz, 1H), 7.70 (d, J = 8.1 Hz, 1H), 8.02 (d, J = 8.1 Hz, 1H), 10.31 (s, 1H), 10.61 (s, 1H); ^{13}C NMR (126 MHz, DMSO-d_6) δ 10.22, 14.17, 20.92, 26.66, 26.85, 28.35 34.04, 35.99, 36.12, 60.03, 60.70, 105.39, 108.38, 114.18, 117.08, 117.99, 119.17, 119.70, 121.91, 123.25, 123.55, 123.97, 124.43, 126.65, 126.79, 126.89, 127.68, 133.00, 134.49, 135.32, 137.67, 138.40, 144.90, 147.15,153.36, 161.57; m/z (ES$^+$), [M+H]$^+$ calcd for $C_{36}H_{39}N_5O_4S_2$, 670.24; found 670.1.

Ethyl 5,13,14-trimethyl-28-oxa-2,9-dithia-5,6,12,13,22-pentaazaheptacyclo[27.7.1.1^{4,7}.0^{11,15}.0^{16,21}.0^{20,24}.0^{30,35}]octatriaconta-1(37),4(38),6,11,14,16,18,20,23,29,31,33,35-tridecaene-23-carboxylate (**A17**)

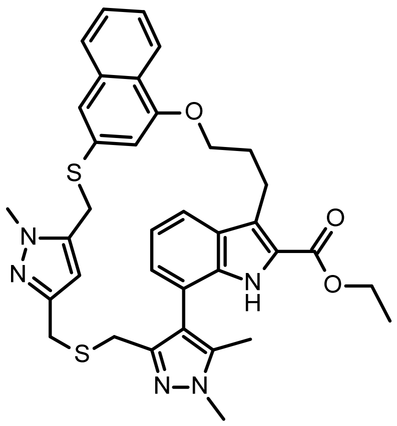

A mixture of ethyl 7-(3-((((5-(((4-hydroxynaphthalen-2-yl)thio)methyl)-1-methyl-1H-pyrazol-3-yl)methyl)thio)methyl)-1,5-dimethyl-1H-pyrazol-4-yl)-3-(3-hydroxypropyl)-1H-indole-2-carboxylate **A16** (500 mg, 0.75 mmol) and di-*tert*-butyl diazene-1,2-dicarboxylate (DTBAD, 344 mg, 1.49 mmol) in toluene (30 mL) was added to a stirred solution of triphenylphosphine (392 mg, 1.49 mmol) in toluene (70 mL) over 1.5 h under N_2. The reaction was continued at RT for 2 h. The mixture was diluted with EtOAc (100 mL), washed with 1M HCl solution, brine, dried over Na_2SO_4, filtered and concentrated to dryness. The residue was purified by silica gel chromatography (0% to 100% EtOAc in hexanes, then 10% MeOH in EtOAc) to yield ethyl 5,13,14-trimethyl-28-oxa-2,9-dithia-5,6,12,13,22-pentaazaheptacyclo[27.7.1.1^{4,7}.0^{11,15}.0^{16,21}.0^{20,24}.0^{30,35}]octatriaconta-1(37),4(38),6,11,14,16,18,20,23,29,31,33,35-tridecaene-23-carboxylate **A17** (367 mg, 75.0%) as a white solid.

^{1}H NMR (300 MHz, DMSO-d_6) δ 1.31 (t, J =7.2 Hz, 3H), 2.10 - 2.25 (m, 1H) 2.30 - 2.50 (m, 1H), 2.72 - 2.81 (m, 1H), 3.03 - 3.18 (m, 2H), 3.29 (s, 3H), 3.37 - 3.43 (m, 2H), 3.54 - 3.59 (m, 1H), 3.67 (s, 3H), 3.71 (s, 3H), 3.80 - 3.86 (m, 1H), 4.06 - 4.11 (m, 1H), 4.22 (s, 2H), 4.30 (q, J = 7.2

Hz, 2H), 4.78 (s, 1H), 6.63 (s, 1H), 6.91 - 6.99 (m, 2H), 7.37 (s, 1H), 7.42 - 7.51 (m, 2H), 7.70 - 7.76 (m, 2H), 8.11 - 8.14 (m, 1H), 10.90 (s, 1H); ^{13}C NMR (126 MHz, DMSO-d_6) δ 10.08, 14.28, 20.02, 26.65, 28.42, 29.57, 36.02, 36.20, 54.89, 59.73, 60.14, 66.99, 104.70, 105.66, 114.37, 118.37, 119.11, 119.77, 121.14, 121.45, 122.29, 123.63, 124.13, 125.39, 126.81, 126.59, 128.13, 131.30, 133.77, 135.66, 137.92, 138.68, 145.18, 146.38, 153.99, 161.58; m/z (ES$^+$), $[M+H]^+$ calcd for $C_{36}H_{37}N_5O_3S_2$, 652.23; found 652.2.

Ethyl 5,13,14,22-tetramethyl-28-oxa-2,9-dithia-5,6,12,13,22-pentaazaheptacyclo[27.7.1.1^{4,7}.0^{11,15}.0^{16,21}.0^{20,24}.0^{30,35}]octatriaconta-1(37),4(38),6,11,14,16,18,20,23,29,31,33,35-tridecaene-23-carboxylate (**A18**)

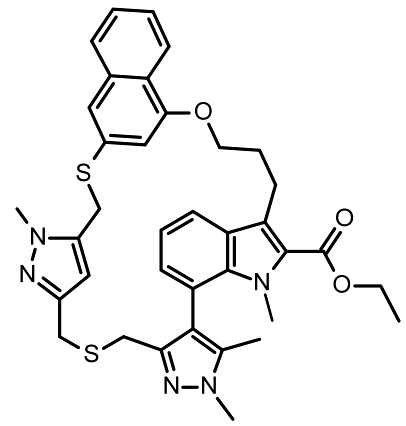

A mixture of ethyl 5,13,14-trimethyl-28-oxa-2,9-dithia-5,6,12,13,22-pentaazaheptacyclo[27.7.1.1^{4,7}.0^{11,15}.0^{16,21}.0^{20,24}.0^{30,35}]octatriaconta-1(37),4(38),6,11,14,16,18,20,23,29,31,33,35-tridecaene-23-carboxylate **A17** (241 mg, 0.37 mmol), iodomethane (0.37 mL, 0.74 mmol) (2M in *tert*-butyl methyl ether) and Cs_2CO_3 (241 mg, 0.74 mmol) in DMF (10 mL) was stirred at RT for 2.5 h. Water (20 mL) was added to give a white suspension. The suspension was extracted with DCM (3 x 10 mL). The combined organic phases were washed with water (10 mL), dried over Na_2SO_4, filtered and concentrated to dryness. The residue was purified by silica gel chromatography (50% to 100% EtOAc in hexanes) to yield ethyl 5,13,14,22-tetramethyl-28-oxa-2,9-dithia-5,6,12,13,22-pentaazaheptacyclo[27.7.1.1^{4,7}.0^{11,15}.0^{16,21}.0^{20,24}.0^{30,35}]octatriaconta-1(37),4(38),6,11,14,16,18,20,23,29,31,33,35-tridecaene-23-carboxylate **A18** (206 mg, 84.0%) as a white solid.

^1H NMR (500 MHz, DMSO-d_6) δ 1.34 (t, J = 7.2 Hz, 3H), 2.00 (s, 3H), 2.20 - 2.30 (m, 1H), 2.35 - 2.47 (m, 1H), 2.81 - 2.84 (m, 1H), 3.03 - 3.06 (m, 1H), 3.12 - 3.16 (m, 2H), 3.26 - 3.29 (m, 1H), 3.41 - 3.43 (m, 1H), 3.56 (s, 3H), 3.69 (s, 3H), 3.73 (s, 3H), 3.80 - 3.87 (m, 1H), 4.06 - 4.12 (m,

1H), 4.21 (s, 2H), 4.32 (q, J = 7.2 Hz, 2H), 4.70 (s, 1H), 6.63 (s, 1H), 6.86 (d, J = 6.7 Hz, 1H), 6.99 (t, J =7.5 Hz, 1H), 7.37 (s, 1H), 7.40 - 7.50 (m, 2H), 7.70 (d, J = 7.3 Hz, 1H), 7.80 (d, J = 7.8 Hz, 1H), 8.11 (d, J = 7.5 Hz, 1H); ^{13}C NMR (126 MHz, DMSO-d_6) δ 9.65, 14.09, 20.39, 26.34, 26.94, 28.50, 29.69, 33.21, 36.07, 36.21, 60.35, 66.96, 104.69, 106.04, 115.97, 117.76,119.67, 119.95, 121.40, 121.64, 122.58, 123.70, 125.49, 126.68, 126.92, 127.14, 128.03, 129.09, 131.13, 133.88, 136.81, 137.45, 138.84, 145.66, 146.18, 153.91, 161.74; m/z (ES^+), $[M+H]^+$ calcd for $C_{37}H_{39}N_5O_3S_2$, 666.25; found 666.1.

5,13,14-Trimethyl-28-oxa-2,9-dithia-5,6,12,13,22-pentaazaheptacyclo[27.7.1.1^{4,7}.0^{11,15}.0^{16,21}.0^{20,24}.0^{30,35}]octatriaconta-1(37),4(38),6,11,14,16,18,20,23,29,31,33,35-tridecaene-23-carboxylic acid (**5**)

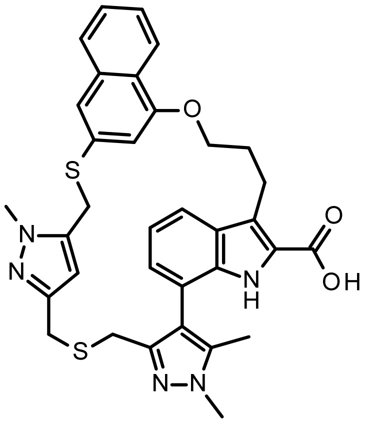

LiOH (90.0 mg, 3.74 mmol) in water (1.00 mL) was added to a mixture of ethyl 5,13,14-trimethyl-28-oxa-2,9-dithia-5,6,12,13,22-pentaazaheptacyclo[27.7.1.1^{4,7}.0^{11,15}.0^{16,21}.0^{20,24}.0^{30,35}] octatriaconta-1(37),4(38),6,11,14,16,18,20,23,29,31,33,35-tridecaene-23-carboxylate **A17** (122 mg, 0.19 mmol) in THF (4.00 mL) and MeOH (2.00 mL). The mixture was stirred at 50 °C for 3 h. The reaction was quenched with ice, 4 mL of 1N HCl solution, diluted with water (10 mL) to give a white suspension. The solid was collected by filtration, washed with water and dried to yield 5,13,14-trimethyl-28-oxa-2,9-dithia-5,6,12,13,22-pentaazaheptacyclo[27.7.1.1^{4,7}.0^{11,15}.0^{16,21}.0^{20,24}.0^{30,35}]octatriaconta-1(37),4(38),6,11,14,16,18,20,23,29,31,33,35-tridecaene-23-carboxylic acid **5** (110 mg, 94.0%), ^{1}H NMR (500 MHz, DMSO-d_6) δ 1.97 (s, 3H), 2.16 - 2.25 (m, 1H), 2.35 - 2.45 (m, 1H), 2.77 - 2.80 (m, 1H), 3.05 - 3.08 (m, 2H), 3.22 - 3.40 (m, 2H), 3.54 - 3.64 (m, 1H), 3.68 (s, 3H), 3.70 (s, 3H), 3.83 - 3.87 (m, 1H), 4.05 - 4.09 (m, 1H), 4.23 (s, 2H), 4.74 (s, 1H), 6.64 (s, 1H), 6.90 - 6.96 (m, 2H), 7.36 (s, 1H) 7.43 - 7.49 (m, 2H), 7.70 - 7.75 (m, 2H), 8.11 (d, J = 7.9 Hz, 1H), 10.76 (s, 1H), 12.95 (s, 1H); ^{13}C NMR (126 MHz, DMSO-d_6) δ 10.05, 19.95, 25.19, 26.63, 28.37, 29.66,

35.99, 36.21, 66.99, 104.88, 105.66, 114.30, 118.29, 119.03, 119.59, 121.15, 121.46, 121.97, 123.66, 124.83, 125.42, 126.48, 126.84, 127.14, 128.30, 131.27, 133.78, 135.53, 137.89, 138.65, 145.05, 146.35, 154.02, 163.18; m/z (ES$^+$), HRMS Calcd for $C_{34}H_{34}N_5O_3S_2$ $[M+H]^+$ 624.2098; found 624.2076.

5,13,14,22-Tetramethyl-28-oxa-2,9-dithia-5,6,12,13,22-pentaazaheptacyclo[27.7.1.1^{4,7}0^{11,15}.0^{16,21}.0^{20,24}.0^{30,35}]octatriaconta-1(37),4(38),6,11,14,16,18,20,23,29,31,33,35-tridecaene-23-carboxylic (**6**)

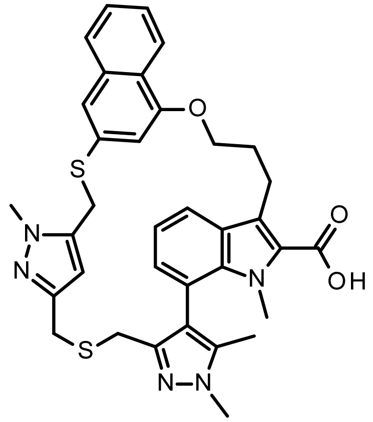

LiOH (140 mg, 5.86 mmol) in water (5.00 mL) was added to a mixture ethyl 5,13,14,22-tetramethyl-28-oxa-2,9-dithia-5,6,12,13,22-pentaazaheptacyclo [27.7.1.1^{4,7}.0^{11,15}.0^{16,21}.0^{20,24}.0^{30,35}]octatriaconta-1(37),4(38),6,11,14,16,18,20,23,29,31,33,35-tridecaene-23-carboxylate **A18** (195 mg, 0.29 mmol) in THF (10 mL) and MeOH (2 mL). The resulting mixture was stirred at 50 °C for 5 h. Ice was added to the mixture, followed by addition of 1M HCl solution (8 mL), water (20 mL) to give a white suspension. The solid was collected by filtration, washed with water and dried to yield 5,13,14,22-tetramethyl-28-oxa-2,9-dithia-5,6,12,13,22-pentaazaheptacyclo[27.7.1.1^{4,7}0^{11,15}.0^{16,21}.0^{20,24}.0^{30,35}]octatriaconta-1(37),4(38),6,11,14,16,18,20,23,29,31,33,35-tridecaene-23-carboxylic **6** (135 mg, 72.3%) as a white solid.

^{1}H NMR (300 MHz, DMSO-d_6) δ 2.00 (s, 3H), 2.19 - 2.29 (m, 1H), 2.32 - 2.42 (m, 1H), 2.82 (d, *J* = 14.3 Hz, 1H), 2.99 - 3.19 (m, 3H), 3.40 - 3.52 (m, 2H), 3.56 (s, 3H), 3.69 (s, 3H), 3.73 (s, 3H), 3.82 - 3.90 (m, 1H), 4.00 - 4.10 (m, 1H), 4.14 - 4.28 (m, 2H), 4.72 (s, 1H), 6.63 (s, 1H), 6.84 (d, *J* = 6.6 Hz, 1H), 6.94 (t, *J* = 6.6 Hz, 1H), 7.37 (s, 1H), 7.41 - 7.50 (m, 2H), 7.66 - 7.74 (m, 1H), 7.78 (d, *J* = 7.5 Hz, 1H), 8.07 - 8.19 (m, 1H), 13.25 (s, 1H); ^{13}C NMR (101 MHz, DMSO-d_6) δ 9.65, 20.32, 26.41, 26.90, 28.48, 29.82, 33.21, 36.06, 36.20, 67.05, 104.78, 106.02, 116.08, 117.68, 119.44, 119.78, 121.40, 121.57, 122.42, 123.73, 125.47, 126.89, 127.11, 127.49,

128.10, 128.82, 131.12, 133.77, 136.81, 137.40, 138.77, 145.70, 146.17, 153.93, 163.40; m/z (ES+), HRMS Calcd for $C_{35}H_{36}N_5O_3S_2$ $[M+H]^+$ 638.2254; found 638.2270.

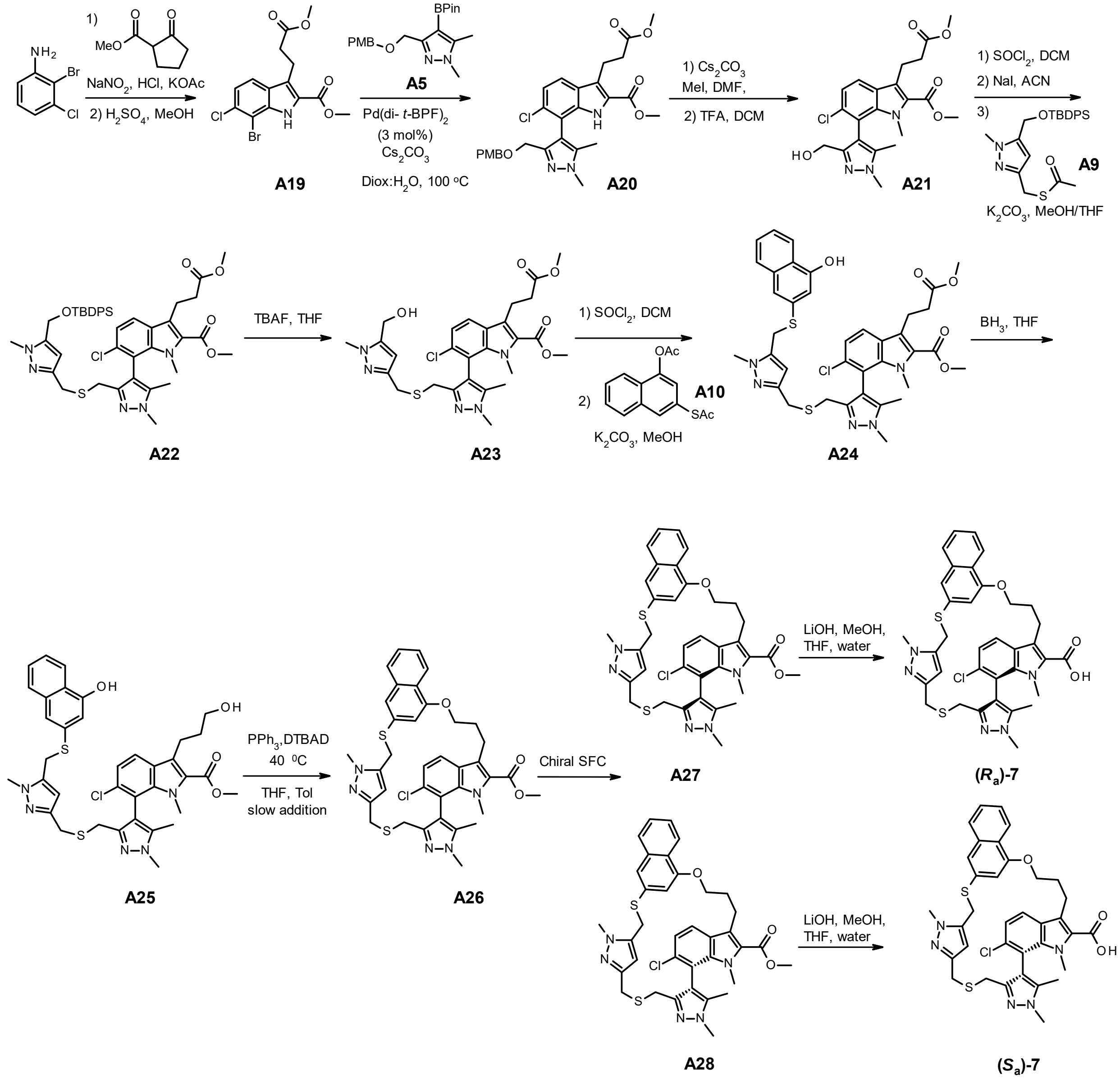

Supplementary Figure 16: Synthetic scheme for compounds **(R_a)-7** and **(S_a)-7**

Methyl 7-bromo-6-chloro-3-(3-methoxy-3-oxopropyl)-1H-indole-2-carboxylate (**A19**)

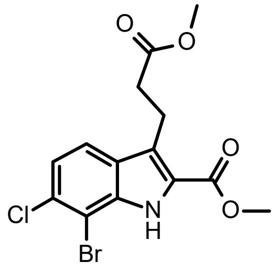

A solution of sodium nitrite (3.59 g, 52.0 mmol) in water (12.2 mL) was added dropwise to a stirred heterogeneous mixture of 2-bromo-3-chloroaniline (10.2 g, 49.6 mmol) in HCl (75 mL, 6 wt%) at 0 °C over a period of 10 min. The resulting mixture was stirred at 0 °C for 60 min. A solution of potassium acetate (34.6 g, 352 mmol) in water (68.7 mL) was added, followed by the addition of methyl 2-oxocyclopentanecarboxylate (6.16 mL, 49.6 mmol). The resulting mixture was stirred at 0 °C for 15 min, then warmed to room temperature and stirred for 2 h. The reaction mixture was extracted with DCM (3 x 200 mL). The combined organic phases were washed with brine (100 mL), dried over anhydrous Na_2SO_4 and concentrated to afford a yellow oil. This oil was dissolved in a mixture of 10 mL of concentrated (conc.) H_2SO_4 in 100 mL of MeOH. It was stirred at 110 °C for 1.5 h then cooled to RT. The MeOH was removed under reduced pressure and water (200 mL) was added to the residue. The aqueous phase was extracted with DCM (3 x 200 mL). The combined organic phases were dried over Na_2SO_4, filtered and concentrated to dryness to give (*E*/*Z*)-dimethyl 2-(2-(2-bromo-3-chlorophenyl)hydrazono)hexanedioate (19.0 g, 48.5 mmol). The mixture was used in the next step without purification.

A solution of *p*-toluenesulfonic acid monohydrate (12.92 g, 67.9 mmol) in anhydrous toluene (150 mL) was refluxed for 1 h. Water was continuously removed by a Dean-Stark trap. (*E*/*Z*)-Dimethyl 2-(2-(2-bromo-3-chlorophenyl)hydrazono)hexanedioate (19.0 g, 48.5 mmol) in anhydrous toluene (60.0 mL) was added. The mixture was stirred at reflux for 1.5 h, cooled to room temperature and then concentrated to dryness. DCM (200 mL) was added. The organic phase was washed with water (50 mL), brine (50 mL), dried over Na_2SO_4, filtered and concentrated to dryness. MeOH (25 mL) was added to the residue to give a suspension. The solid was collected by filtration and washed with MeOH (10 mL) to give the first batch of product.

The MeOH mother liquid was concentrated to dryness. The residue was purified by silica gel chromatography (hexanes/DCM) to give a second batch of product. The total amount of methyl 7-bromo-6-chloro-3-(3-methoxy-3-oxopropyl)-1H-indole-2-carboxylate **A19** was 11.50 g

(63.3%). ^{1}H NMR (400 MHz, CHLOROFORM- *d*) δ 2.68 (t, *J* = 7.6 Hz, 2H), 3.37 (t, *J* = 7.6 Hz, 2H), 3.63 (s, 3H), 3.98 (s, 3H), 7.23 (d, *J* = 8.7 Hz, 1H), 7.61 (d, *J* = 8.7 Hz, 1H), 8.80 (s, 1H); ^{13}C NMR (75 MHz, CHLOROFORM-*d*) δ 20.25, 34.73, 51.57, 52.04, 105.19, 120.51, 122.31, 124.07, 124.29, 126.57, 131.24, 135.36, 161.56, 173.20; m/z (ES$^+$), [M+H]$^+$ calcd for $C_{14}H_{13}BrClNO_4$, 373.97; found 374.0.

(±) Methyl 6-chloro-3-(3-methoxy-3-oxopropyl)-7-(3-(((4-methoxybenzyl)oxy)methyl)-1,5-dimethyl-1H-pyrazol-4-yl)-1H-indole-2-carboxylate (**A20**)

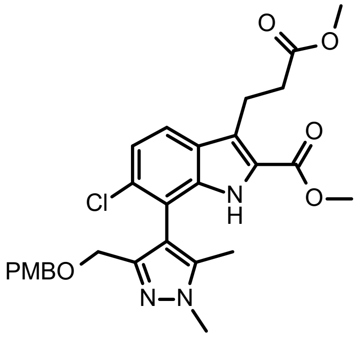

3-((4-Methoxybenzyl)oxy)methyl)-1,5-dimethyl-4-(4,4,5,5-tetramethyl-1,3,2-dioxaborolan-2-yl)-1H-pyrazole **A5** (18.6 g, 50.1 mmol) was dissolved in a mixture of 1,4-dioxane and water (4:1, 100 mL). Cs_2CO_3 (26.1 g, 80.1 mmol), methyl 7-bromo-6-chloro-3-(3-methoxy-3-oxopropyl)-1H-indole-2-carboxylate **A19** (15.0 g, 40.0 mmol) and dichloro[1,1'-bis(di-*tert*-butylphosphino)ferrocene] palladium(II) (0.783 g, 1.20 mmol) were added, followed by additional dioxane and water (300 mL, 4:1). The mixture was degassed and filled with N_2 three times. The resulting brown clear mixture was placed in an oil bath preheated to 100 °C. The mixture was stirred at 100 °C for 3 h. The mixture was cooled to RT and concentrated to 100 mL. EtOAc (200 mL) and water (100 mL) were added. The layers were separated and the aqueous phase was extracted with EtOAc (3 x 100 mL). The combined organic phases were dried over Na_2SO_4, filtered and concentrated. The residue was purified by silica gel column chromatography (hexanes/EtOAc) to give (±) methyl 6-chloro-3-(3-methoxy-3-oxopropyl)-7-(3-(((4-methoxybenzyl)oxy)methyl)-1,5-dimethyl-1H-pyrazol-4-yl)-1H-indole-2-carboxylate **A20** (20.0 g, 92.0%).

^{1}H NMR (400 MHz, CHLOROFORM-*d*) δ 2.11 (s, 3H), 2.73 (t, *J* = 7.5 Hz, 2H), 3.39 - 3.50 (m, 2H), 3.68 (s, 3H), 3.75 (s, 3H), 3.78 (s, 3H), 3.91 (s, 3H), 4.14 (d, *J* = 10.9 Hz, 1H), 4.33 - 4.40 (m, 3H), 6.73 (d, *J* = 8.8 Hz, 2H), 7.00 (d, *J* = 8.8 Hz, 2H), 7.25 (d, *J* = 8.6 Hz, 1H), 7.64 (d, *J* = 8.6 Hz, 1H), 9.17 (s, 1H); ^{13}C NMR (126 MHz, CHLOROFORM-*d*) δ 10.52, 20.09, 34.77, 36.52, 51.44, 51.50, 55.07, 63.94, 72.32, 112.23, 113.45, 115.94, 120.75, 121.96, 123.42, 123.96,

125.98, 129.36, 129.57, 131.48, 136.62, 138.90, 146.01, 158.95, 161.55, 173.42; m/z (ES^+), $[M+H]^+$ calcd for $C_{28}H_{30}ClN_3O_6$, 540.18; found 540.2.

(±) Methyl 6-chloro-7-(3-(hydroxymethyl)-1,5-dimethyl-1H-pyrazol-4-yl)-3-(3-methoxy-3-oxopropyl)-1-methyl-1H-indole-2-carboxylate (**A21**)

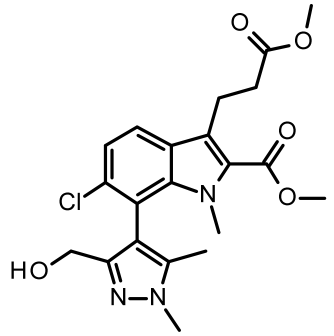

(±) Methyl 6-chloro-3-(3-methoxy-3-oxopropyl)-7-(3-(((4-methoxybenzyl)oxy)methyl)-1,5-dimethyl-1H-pyrazol-4-yl)-1H-indole-2-carboxylate **A20** (22.2 g, 37.2 mmol, 90.5 wt%) was dissolved in anhydrous DMF (100 mL). Cs_2CO_3 (18.2 g, 55.8 mmol) was added. The mixture was stirred for 20 min and MeI (4.65 mL, 74.4 mmol) was added. The mixture was stirred for 2.5 h. Water (300 mL) was added and the aqueous phase was extracted with EtOAc (3 x 100 mL). The combined organic phases were concentrated to dryness. The residue was dissolved in EtOAc (300 mL) and the resulting solution was washed with water (3 x 50 mL) to further remove DMF. The organic phase was dried over Na_2SO_4, filtered and concentrated to dryness to give (±) methyl 6-chloro-3-(3-methoxy-3-oxopropyl)-7-(3-(((4-methoxybenzyl)oxy)methyl)-1,5-dimethyl-1H-pyrazol-4-yl)-1-methyl-1H-indole-2-carboxylate (22.1 g, 100%, 93.1 wt%), which was used without purification

(±) Methyl 6-chloro-3-(3-methoxy-3-oxopropyl)-7-(3-(((4-methoxybenzyl)oxy)methyl)-1,5-dimethyl-1H-pyrazol-4-yl)-1-methyl-1H-indole-2-carboxylate (22.1 g, 37.3 mmol) was dissolved in DCM (56 mL) and TFA (28.7 mL, 373 mmol) was added at 0 °C. The ice bath was removed and the mixture was stirred at RT for 1.5 h. DCM (200 mL) was added. The organic phase was washed sequentially with water (3 x 75 mL) and sat. aq. $NaHCO_3$ (2 x 50 mL) and the aqueous phase was extracted with DCM (100 mL). The organic phases were combined and 2 mL of MeOH and Et_3N (2 mL) were added. The mixture was stirred for 30 min and concentrated to dryness. Water (50 mL) was added and the aqueous phase was extracted with DCM (3 x 100 mL). The organic phase was dried over Na_2SO_4, filtered and concentrated. The residue was purified by silica gel column chromatography (DCM/EtOAc then 10% MeOH in DCM) to (±) give

methyl 6-chloro-7-(3-(hydroxymethyl)-1,5-dimethyl-1H-pyrazol-4-yl)-3-(3-methoxy-3-oxopropyl)-1-methyl-1H-indole-2-carboxylate **A21** (13.7 g, 85.0%).

^{1}H NMR (400 MHz, CHLOROFORM-*d*) δ 2.07 (s, 3H), 2.67 (t, *J* = 7.9 Hz, 2H), 3.34 (t, *J* = 7.9 Hz, 2H), 3.54 (s, 3H), 3.69 (s, 3H), 3.92 (s, 3H), 3.93 (s, 3H), 4.42 (AB, d, *J* = 12.8 Hz, 1H), 4.48 (AB, d, *J* = 12.8 Hz, 1H), 7.24 (d, *J* = 8.7 Hz, 1H), 7.65 (d, *J* = 8.7 Hz, 1H); ^{13}C NMR (126 MHz, CHLOROFORM-*d*) δ 10.03, 20.77, 33.85, 35.20, 36.49, 51.64, 51.73, 57.82, 113.16, 115.62, 121.11, 121.71, 123.20, 126.03, 127.15, 133.76, 138.23, 138.61, 149.30, 162.53, 173.51; m/z (ES^{+}), $[M+H]^{+}$ calcd for $C_{21}H_{24}ClN_3O_5$, 434.14; found 434.1.

(±) Methyl 7-(3-((((5-((*tert*-butyldiphenylsilyl)oxy)methyl)-1-methyl-1H-pyrazol-3-yl)methylthio)methyl)-1,5-dimethyl-1H-pyrazol-4-yl)-6-chloro-3-(3-methoxy-3-oxopropyl)-1-methyl-1H-indole-2-carboxylate (**A22**)

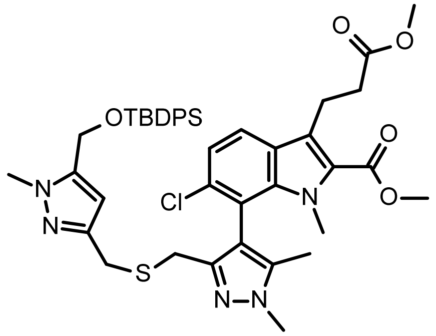

(±) Methyl 6-chloro-7-(3-(hydroxymethyl)-1,5-dimethyl-1H-pyrazol-4-yl)-3-(3-methoxy-3-oxopropyl)-1-methyl-1H-indole-2-carboxylate **A21** (13.0 g, 29.9 mmol) was dissolved in DCM (150 mL) under Ar. The solution was cooled to 0 °C and thionyl chloride (2.62 mL, 35.9 mmol) was added. The ice bath was removed and the mixture was stirred at RT for 30 min, then concentrated. DCM (50 mL) was added and the resulting solution was washed sequentially with sat. aq. $NaHCO_3$ and brine, dried over Na_2SO_4, filtered and concentrated to give (±) methyl 6-chloro-7-(3-(chloromethyl)-1,5-dimethyl-1H-pyrazol-4-yl)-3-(3-methoxy-3-oxopropyl)-1-methyl-1H-indole-2-carboxylate (13.6 g, 100%) which was used without purification.

(±) Methyl 6-chloro-7-(3-(chloromethyl)-1,5-dimethyl-1H-pyrazol-4-yl)-3-(3-methoxy-3-oxopropyl)-1-methyl-1H-indole-2-carboxylate (13.5 g, 29.9 mmol) was dissolved in acetonitrile (100 mL) and sodium iodide (7.86 g, 52.4 mmol) was added. The mixture was stirred at 80 °C for 2.5 h. After cooling to RT, the mixture was filtered through a pad of diatomaceous earth and concentrated. Water (100 mL) and EtOAc (100 mL) were added, the layers were separated and the aqueous phase was extracted with EtOAc (2 x 100 mL). The combined organics were dried over Na_2SO_4, filtered and concentrated to give (±) methyl 6-chloro-7-(3-(iodomethyl)-1,5-

dimethyl-1H-pyrazol-4-yl)-3-(3-methoxy-3-oxopropyl)-1-methyl-1H-indole-2-carboxylate (15.7 g, 96.0%) which was used without purification.

(±) Methyl 6-chloro-7-(3-(iodomethyl)-1,5-dimethyl-1H-pyrazol-4-yl)-3-(3-methoxy-3-oxopropyl)-1-methyl-1H-indole-2-carboxylate (7.60 g, 13.9 mmol) was dissolved in MeOH (30 mL) and THF (15 mL) to give a suspension. K_2CO_3 (1.93 g, 13.9 mmol) was added. The mixture was degassed and filled with N_2. S-((5-(((*tert*-Butyldiphenylsilyl)oxy)methyl)-1-methyl-1H-pyrazol-3-yl)methyl)ethanethioate **A9** (6.74 g, 15.4 mmol) in MeOH (15 mL) was added dropwise over 5 min. After addition of the thioacetate solution, the mixture was degassed, filled with N_2 again and then stirred for 2 h. The mixture was concentrated to dryness and EtOAc (100 mL) was added. The organic phase was washed with water, dried over Na_2SO_4 and concentrated. The residue was purified by silica gel column chromatography (hexanes/EtOAc) to give (±) methyl 7-(3-((((5-(((*tert*-butyldiphenylsilyl)oxy)methyl)-1-methyl-1H-pyrazol-3-yl)methyl)thio)methyl)-1,5-dimethyl-1H-pyrazol-4-yl)-6-chloro-3-(3-methoxy-3-oxopropyl)-1-methyl-1H-indole-2-carboxylate **A22** (7.10 g, 63.0%).

^{1}H NMR (400 MHz, CHLOROFORM-*d*) δ 1.04 (s, 9H), 2.04 (s, 3H), 2.65 (t, *J* = 7.9 Hz, 2H), 3.32 (t, *J* = 7.9 Hz, 2H), 3.52 - 3.57 (m, 5H), 3.61 (s, 2H), 3.68 (s, 3H), 3.79 (s, 3H), 3.89 (s, 3H), 3.91 (s, 3H), 4.58 (s, 2H), 5.93 (s, 1H), 7.22 (d, *J* = 8.7 Hz, 1H), 7.35 - 7.49 (m, 6H), 7.58 (d, *J* = 8.7 Hz, 1H), 7.61 - 7.71 (m, 4H); ^{13}C NMR (75 MHz, CHLOROFORM-*d*) δ 10.10, 19.18, 20.82, 26.71, 27.69, 29.02, 33.85, 35.23, 36.50, 36.51, 51.58, 51.62, 56.94, 104.99, 113.64, 116.11, 120.83, 121.72, 123.10, 125.93, 127.04, 127.79, 129.88, 132.86, 133.85, 135.55, 138.23, 138.29, 141.66, 147.15, 147.85, 162.55, 173.50; m/z (ES$^+$), [M+H]$^+$ calcd for $C_{43}H_{50}ClN_5O_5SSi$, 812.30; found 812.2.

(±) Methyl 6-chloro-7-(3-((((5-(hydroxymethyl)-1-methyl-1H-pyrazol-3-yl)methyl)thio)methyl)-1,5-dimethyl-1H-pyrazol-4-yl)-3-(3-methoxy-3-oxopropyl)-1-methyl-1H-indole-2-carboxylate (**A23**)

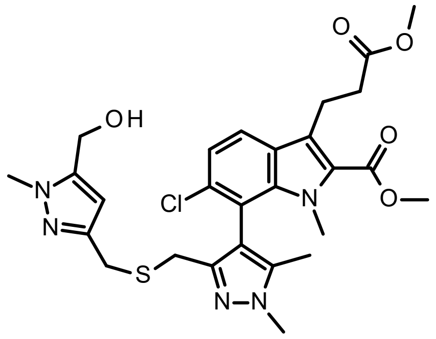

(±) Methyl 7-(3-((((5-(((*tert*-butyldiphenylsilyl)oxy)methyl)-1-methyl-1H-pyrazol-3-yl)methyl)thio)methyl)-1,5-dimethyl-1H-pyrazol-4-yl)-6-chloro-3-(3-methoxy-3-oxopropyl)-1-methyl-1H-indole-2-carboxylate **A22** (13.9 g, 17.1 mmol) was dissolved in THF (40 mL) and

TBAF (17.1 mL, 17.1 mmol) (1 M in THF) was added. The mixture was stirred for 1 h and then concentrated. EtOAc (200 mL) was added and the organic phase was washed sequentially with water and brine, dried over Na_2SO_4, filtered and concentrated. The residue was purified by silica gel column chromatography (hexanes/EtOAc) to (±) give methyl 6-chloro-7-(3-((((5-(hydroxymethyl)-1-methyl-1H-pyrazol-3-yl)methyl)thio)methyl)-1,5-dimethyl-1H-pyrazol-4-yl)-3-(3-methoxy-3-oxopropyl)-1-methyl-1H-indole-2-carboxylate **A23** (8.40 g, 86.0%).

^{1}H NMR (400 MHz, CHLOROFORM-*d*) δ 2.05 (s, 3H), 2.68 (dd, *J* = 8.5, 7.0 Hz, 2H), 3.35 (dd, *J* = 8.5, 7.0 Hz, 2H), 3.52 - 3.59 (m, 7H), 3.67 (s, 3H), 3.78 (s, 3H), 3.88 (s, 3H), 3.93 (s, 3H), 4.56 (s, 2H), 5.95 (s, 1H), 7.24 (d, *J* =8.7 Hz, 1H), 7.64 (d, *J* =8.7 Hz, 1H); ^{13}C NMR (75 MHz, CHLOROFORM-*d*) δ 9.90, 20.60, 27.42, 28.75, 33.64, 35.02, 36.02, 36.18, 51.42, 51.50, 54.83, 104.72, 113.49, 115.80, 120.75, 121.51, 122.91, 125.76, 126.87, 133.61, 138.25, 138.04, 142.30, 146.83, 147.72, 162.38, 173.35; m/z (ES$^+$), [M+H]$^+$ calcd for $C_{27}H_{32}ClN_5O_5S$, 574.18; found 574.2.

(±) Methyl 6-chloro-7-(3-((((5-(((4-hydroxynaphthalen-2-yl)thio)methyl)-1-methyl-1H-pyrazol-3-yl)methyl)thio)methyl)-1,5-dimethyl-1H-pyrazol-4-yl)-3-(3-methoxy-3-oxopropyl)-1-methyl-1H-indole-2-carboxylate (**A24**)

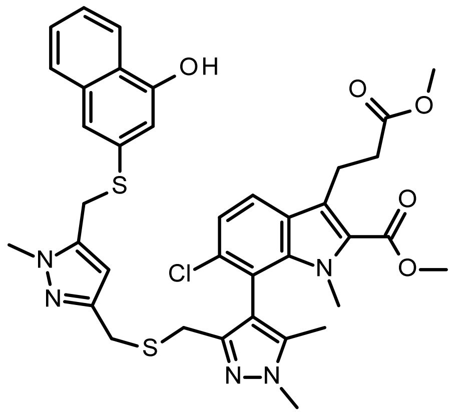

(±) Methyl 6-chloro-7-(3-((((5-(hydroxymethyl)-1-methyl-1H-pyrazol-3-yl)methyl)thio)methyl)-1,5-dimethyl-1H-pyrazol-4-yl)-3-(3-methoxy-3-oxopropyl)-1-methyl-1H-indole-2-carboxylate **A23**, (8.70 g, 15.2 mmol) was dissolved in anhydrous DCM (100 mL) under Ar. The mixture was cooled to 0 °C. Thionyl chloride (1.33 mL, 18.2 mmol) was added. The ice bath was removed. The mixture was stirred at RT for 30 min and then concentrated. DCM (50 mL) was added. The resulting solution was washed sequentially with water, sat. aq. $NaHCO_3$ and brine, dried over Na_2SO_4, filtered and concentrated to give (±) methyl 6-chloro-7-(3-((((5-(chloromethyl)-1-methyl-1H-pyrazol-3-yl)methyl)thio)methyl)-1,5-dimethyl-1H-pyrazol-4-yl)-3-(3-methoxy-3-oxopropyl)-1-methyl-1H-indole-2-carboxylate(9.00 g, 100%), which was used without

purification.

K_2CO_3 (5.15 g, 37.3 mmol) was added to a mixture of (±) methyl 6-chloro-7-(3-((((5-(chloromethyl)-1-methyl-1H-pyrazol-3-yl)methyl)thio)methyl)-1,5-dimethyl-1H-pyrazol-4-yl)-3-(3-methoxy-3-oxopropyl)-1-methyl-1H-indole-2-carboxylate (9.20 g, 15.5 mmol) and 3-(acetylthio)naphthalen-1-yl acetate **A10** (4.45 g, 17.1 mmol) in MeOH (120 mL). The resulting mixture was stirred for 1 h at RT. The reaction mixture was evaporated to dryness. The residue was redissolved in EtOAc (150 mL). The resulting solution was washed sequentially with water (2 x 100 mL) and brine (100 mL). The organic layer was dried over Na_2SO_4, filtered and concentrated. The residue was purified by silica gel column chromatography (0-10% MeOH in DCM) to give (±) methyl 6-chloro-7-(3-((((5-(((4-hydroxynaphthalen-2-yl)thio)methyl)-1-methyl-1H-pyrazol-3-yl)methyl)thio)methyl)-1,5-dimethyl-1H-pyrazol-4-yl)-3-(3-methoxy-3-oxopropyl)-1-methyl-1H-indole-2-carboxylate **A24** (7.42 g, 65.3%).

^{1}H NMR (300 MHz, CHLOROFORM-*d*) δ 2.09 (s, 3H), 2.62 - 2.74 (m, 2H), 3.31 - 3.66 (m, 12H), 3.70 (s, 3H), 3.94 - 3.96 (m, 8H), 6.07 (s, 1H), 6.65 (d, *J* = 1.2, Hz, 1H) 7.24 (d, *J* = 8.7 Hz, 1H), 7.43 - 7.56 (m, 2H), 7.59 - 7.71 (m, 2H), 7.71 - 7.80 (m, 1H), 8.19 - 8.30 (m, 1H); ^{13}C NMR (75 MHz, CHLOROFORM-*d*) δ 10.11, 20.90, 26.66, 28.85, 31.09, 34.01, 35.26, 35.91, 36.09, 51.69, 51.74, 105.73, 113.65, 114.51, 115.32, 121.25, 121.80, 122.61, 123.28, 125.12, 125.35, 125.55, 126.07, 126.85, 127.13, 127.21, 130.89, 134.07, 134.73, 138.22, 139.44, 140.68, 146.66, 148.06, 153.14, 162.60, 173.67; m/z (ES$^+$), $[M+H]^+$ calcd for $C_{37}H_{38}ClN_5O_5S_2$, 732.20; found 732.3.

(±) Methyl 6-chloro-7-(3-((((5-(((4-hydroxynaphthalen-2-yl)thio)methyl)-1-methyl-1H-pyrazol-3-yl)methyl)thio)methyl)-1,5-dimethyl-1H-pyrazol-4-yl)-3-(3-hydroxypropyl)-1-methyl-1H-indole-2-carboxylate (**A25**)

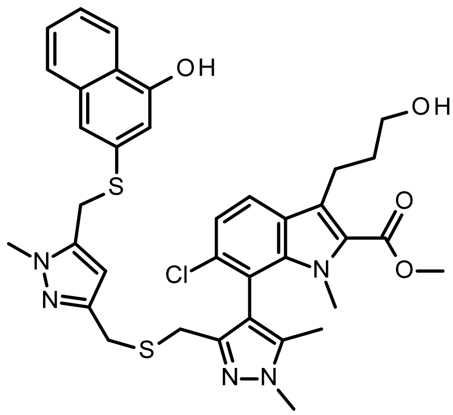

(±) Methyl 6-chloro-7-(3-((((5-(((4-hydroxynaphthalen-2-yl)thio)methyl)-1-methyl-1H-pyrazol-3-yl)methyl)thio)methyl)-1,5-dimethyl-1H-pyrazol-4-yl)-3-(3-methoxy-3-oxopropyl)-1-

methyl-1H-indole-2-carboxylate **A24** (5.00 g, 6.83 mmol) was dissolved in THF (20 mL) under Ar. The resulting solution was cooled to 0 °C and borane tetrahydrofuran complex (37.6 mL, 37.6 mmol) (1 M in THF) was added. The ice bath was removed and the mixture was stirred at RT for 5.5 h. The reaction mixture was concentrated and cooled to 0 °C, followed by addition of MeOH (20 mL) and 6 N HCl (40 mL) (exothermic). The resulting solution was stirred at 0 °C for 10 min, then at RT for 20 min. The volume of the mixture was reduced to 1/3 under reduced pressure. Water (200 mL) was added and the aqueous phase was extracted with 10% MeOH in DCM (9 x 50 mL). The combined organic phases was washed sequentially with sat. aq. $NaHCO_3$ (50 mL) and brine, dried over Na_2SO_4, filtered and concentrated. The residue was purified by silica gel column chromatography (hexanes/EtOAc) to give (±) methyl 6-chloro-7-(3-((((5-(((4-hydroxynaphthalen-2-yl)thio)methyl)-1-methyl-1H-pyrazol-3-yl)methyl)thio)methyl)-1,5-dimethyl-1H-pyrazol-4-yl)-3-(3-hydroxypropyl)-1-methyl-1H-indole-2-carboxylate **A25** (4.05 g, 84.0%).

^{1}H NMR (400 MHz, CHLOROFORM-*d*) δ 1.93 - 2.03 (m, 2H), 2.10 (s, 3H), 3.18 (t, *J* = 7.2 Hz, 2H), 3.41 - 3.64 (m, 10H), 3.68 (t, *J* = 5.9 Hz, 2H), 3.91 - 3.98 (m, 8H), 6.05 (s, 1H), 6.64 (d, *J* = 1.2 Hz, 1H), 7.25 (d, *J* = 8.7 Hz, 1H) 7.43 - 7.58 (m, 2H), 7.61 - 7.68 (m, 2H), 7.72 - 7.81 (m, 1H), 8.26 (d, *J* = 7.4 Hz, 1H); ^{13}C NMR (75 MHz, CHLOROFORM-*d*) δ 10.13, 21.04, 26.63, 28.72, 30.97, 33.34, 34.24, 35.91, 36.11, 51.85, 61.68, 105.80, 113.44, 114.55, 115.27, 121.42, 121.70, 122.59, 124.97, 125.09, 125.18, 125.54, 126.46, 126.87, 127.20, 127.50, 130.83, 134.08, 134.73, 138.45, 139.54, 140.82, 146.61, 147.99, 153.19, 163.05; m/z (ES$^+$), $[M+H]^+$ calcd for $C_{36}H_{38}ClN_5O_4S_2$, 704.21; found 704.3.

(±) Methyl 17-chloro-5,13,14,22-tetramethyl-28-oxa-2,9-dithia-5,6,12,13,22-pentaazaheptacyclo[27.7.1.1^{4,7}.0^{11,15}.0^{16,21}.0^{20,24}.0^{30,35}]octatriaconta-1(37),4(38),6,11,14,16,18,20,23,29,31,33,35-tridecaene-23-carboxylate (**A26**)

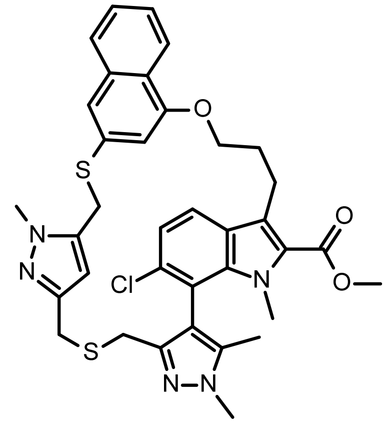

Triphenylphosphine (1.58 g, 6.02 mmol) was dissolved in toluene (30 mL) and a solution of di-*tert*-butyl diazene-1,2-dicarboxylate (1.39 g, 6.02 mmol) and (±) methyl 6-chloro-7-(3-((((5-(((4-hydroxynaphthalen-2-yl)thio)methyl)-1-methyl-1H-pyrazol-3-yl)methyl)thio)methyl)-1,5-dimethyl-1H-pyrazol-4-yl)-3-(3-hydroxypropyl)-1-methyl-1H-indole-2-carboxylate **A25** (2.12 g, 3.01 mmol) in toluene (27.6 mL) and THF (2.50 mL) was added via addition funnel over 1 h. After addition, the mixture was stirred for 1 h. The reaction mixture was diluted with EtOAc (50 mL) and MeOH (5 mL) and then washed sequentially with water, 2 N HCl and brine, dried over Na_2SO_4, filtered and concentrated. MeOH (10 mL) was added to the resulting residue. The mixture was sonicated for 5 min to result in a white suspension. The solid was collected, washed with MeOH (6 mL) and dried to give the first batch of product (1.34 g, 64.0%). The mother liquor was concentrated and the residue was purified by silica gel column chromatography (hexanes/EtOAc) to give the second batch of product. The total amount of (±) methyl 17-chloro-5,13,14,22-tetramethyl-28-oxa-2,9-dithia-5,6,12,13,22-pentaazaheptacyclo[27.7.1.1^{4,7}.0^{11,15}.0^{16,21}.0^{20,24}.0^{30,35}]octatriaconta-1(37),4(38),6,11,14,16,18,20,23,29,31,33,35-tridecaene-23-carboxylate **A26** was 1.40 g (68.0%).

^{1}H NMR (400 MHz, CHLOROFORM-*d*) δ 2.05 (s, 3H), 2.22 - 2.25 (m, 1H), 2.38 - 2.51 (m, 1H), 2.68 (AB, d, *J* = 13.8 Hz, 1H), 3.09 (AB, d, *J* = 13.8 Hz, 1H), 3.21 - 3.32 (m, 2H), 3.45 - 3.56 (m, 2H), 3.63 - 3.73 (m, 4H), 3.75 - 3.84 (m, 4H), 3.84 - 3.96 (m, 8H), 4.92 (s, 1H), 6.25 (d, *J* = 1.2 Hz, 1H), 6.95 (d, *J* = 8.6 Hz, 1H), 7.50 - 7.59 (m, 4H), 7.70 - 7.81 (m, 1H), 8.22 - 8.38 (m, 1H); ^{13}C NMR (126 MHz, CHLOROFORM-*d*) δ 10.15, 20.85, 26.83, 28.60, 30.00, 30.26, 33.96, 36.44, 36.47, 51.69, 66.00, 105.63, 108.64, 113.23, 116.46, 121.04, 121.31, 121.86, 123.52, 125.08, 125.30, 125.93, 127.06, 127.36, 127.39, 130.84, 133.63, 134.18, 138.15, 138.22, 139.46, 147.33, 148.67, 154.29, 162.68; m/z (ES$^+$), $[M+H]^+$ calcd for $C_{36}H_{36}ClN_5O_3S_2$, 686.19; found 686.2.

(*R*$_a$)-(+)-Methyl 17-chloro-5,13,14,22-tetramethyl-28-oxa-2,9-dithia-5,6,12,13,22-pentaazaheptacyclo[27.7.1.1^{4,7}.0^{11,15}.0^{16,21}.0^{20,24}.0^{30,35}]octatriaconta-1(37),4(38),6,11,14,16,18,20,23,29,31,33,35-tridecaene-23-carboxylate (**A27**) and (*S*$_a$)-(-)-methyl 17-chloro-5,13,14,22-tetramethyl-28-oxa-2,9-dithia-5,6,12,13,22-pentaazaheptacyclo[27.7.1.1^{4,7}.0^{11,15}.0^{16,21}.0^{20,24}.0^{30,35}]octatriaconta-1(37),4(38),6,11,14,16,18,20,23,29,31,33,35-tridecaene-23-carboxylate (**A28**)

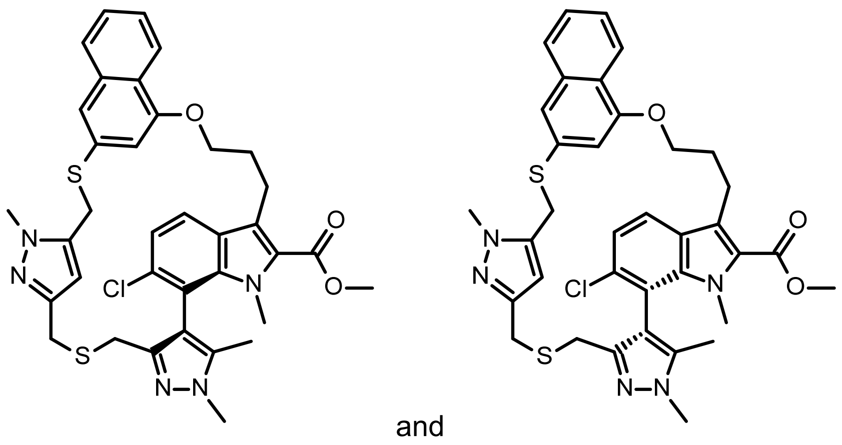

(±) Methyl 17-chloro-5,13,14,22-tetramethyl-28-oxa-2,9-dithia-5,6,12,13,22-pentaazaheptacyclo[27.7.1.1^{4,7}.0^{11,15}.0^{16,21}.0^{20,24}.0^{30,35}]octatriaconta-1(37),4(38),6,11,14,16,18,20,23,29,31,33,35-tridecaene-23-carboxylate **A26** (4.70 g, 6.85 mmol) was subjected to chiral supercritical fluid chromatography (SFC, Chiralpak IA® column, 21 x 250 mm, 5 µm, Temperature = 40 °C, 45:55 *i*-PrOH:CO_2, UV detection @ 220 nm, loading= 150 mg/inj, conc = 60 mg/mL, Diluent=MeOH/DCM, flow rate = 60 mL/min, Outlet Pressure = 100 bar).

(R_a)-(+)-Methyl 17-chloro-5,13,14,22-tetramethyl-28-oxa-2,9-dithia-5,6,12,13,22-pentaazaheptacyclo[27.7.1.1^{4,7}.0^{11,15}.0^{16,21}.0^{20,24}.0^{30,35}]octatriaconta-1(37),4(38),6,11,14,16,18,20,23,29,31,33,35-tridecaene-23-carboxylate (**A27**) was eluted first (1.87 g, 37.0%, >98.0% enantiomeric excess (e.e.)).

^{1}H NMR (400 MHz, CHLOROFORM-*d*) δ 2.05 (s, 3H), 2.22 - 2.25 (m, 1H), 2.38 - 2.51 (m, 1H), 2.68 (AB, d, *J* = 13.8 Hz, 1H), 3.09 (AB, d, *J* = 13.8 Hz, 1H), 3.21 - 3.32 (m, 2H), 3.45 - 3.56 (m, 2H), 3.63 - 3.73 (m, 4H), 3.75 - 3.84 (m, 4H), 3.84 - 3.96 (m, 8H), 4.92 (s, 1H), 6.25 (d, *J* = 1.2 Hz, 1H), 6.95 (d, *J* = 8.6 Hz, 1H), 7.50 - 7.59 (m, 4H), 7.70 - 7.81 (m, 1H), 8.22 - 8.38 (m, 1H); ^{13}C NMR (126 MHz, CHLOROFORM-*d*) δ 10.15, 20.85, 26.83, 28.60, 30.00, 30.26, 33.96, 36.44, 36.47, 51.69, 66.00, 105.63, 108.64, 113.23, 116.46, 121.04, 121.31, 121.86, 123.52, 125.08, 125.30, 125.93, 127.06, 127.36, 127.39, 130.84, 133.63, 134.18, 138.15, 138.22, 139.46, 147.33, 148.67, 154.29, 162.68; m/z (ES$^+$), [M+H]$^+$ calcd for $C_{36}H_{36}ClN_5O_3S_2$, 686.19; found 686.2.

Post Purification e.e. purity check:

Chiral analysis method: SFC: Chiralpak IA® column, 4.6 x 100 mm, 5 µm, Temperature = 40 °C, 35:65 i-PrOH:CO_2, UV detection at 220 nm, flow rate = 5.0 mL/min, Outlet Pressure =125 bar. Retention time of 1.63 min, >98.0% e.e., $[\alpha]_D$ +64° (*c* = 0.1, MeOH)

(S_a)-(-)-Methyl 17-chloro-5,13,14,22-tetramethyl-28-oxa-2,9-dithia-5,6,12,13,22-pentaazaheptacyclo[27.7.1.1^{4,7}.0^{11,15}.0^{16,21}.0^{20,24}.0^{30,35}]octatriaconta-1(37),4(38),6,11,14,16,18,20,23,29,31,33,35-tridecaene-23-carboxylate (**A28**) was eluted second (1.40 g, 28.0%, >98.0% e.e.).

^{1}H NMR (400 MHz, CHLOROFORM-*d*) δ 2.05 (s, 3H), 2.22 - 2.25 (m, 1H), 2.38 - 2.51 (m, 1H), 2.68 (AB, d, *J* = 13.8 Hz, 1H), 3.09 (AB, d, *J* = 13.8 Hz, 1H), 3.21 - 3.32 (m, 2H), 3.45 - 3.56 (m, 2H), 3.63 - 3.73 (m, 4H), 3.75 - 3.84 (m, 4H), 3.84 - 3.96 (m, 8H), 4.92 (s, 1H), 6.25 (d, *J* = 1.2 Hz, 1H), 6.95 (d, *J* = 8.6 Hz, 1H), 7.50 - 7.59 (m, 4H), 7.70 - 7.81 (m, 1H), 8.22 - 8.38 (m, 1H); ^{13}C NMR (126 MHz, CHLOROFORM-*d*) δ 10.15, 20.85, 26.83, 28.60, 30.00, 30.26, 33.96, 36.44, 36.47, 51.69, 66.00, 105.63, 108.64, 113.23, 116.46, 121.04, 121.31, 121.86, 123.52, 125.08, 125.30, 125.93, 127.06, 127.36, 127.39, 130.84, 133.63, 134.18, 138.15, 138.22, 139.46, 147.33, 148.67, 154.29, 162.68; m/z (ES$^+$), [M+H]$^+$ calcd for $C_{36}H_{36}ClN_5O_3S_2$, 686.19; found 686.2.

Post Purification e.e. purity check:
Chiral analysis method as for **A27**. Retention time of 3.77 min, >98.0% e.e., $[\alpha]_D$ -64° (*c* = 0.1, MeOH)

(R_a)-(+)-17-Chloro-5,13,14,22-tetramethyl-28-oxa-2,9-dithia-5,6,12,13,22-pentaazaheptacyclo[27.7.1.1^{4,7}.0^{11,15}.0^{16,21}.0^{20,24}.0^{30,35}]octatriaconta-1(37),4(38),6,11,14,16,18,20,23,29,31,33,35-tridecaene-23-carboxylic acid (**(R_a)-7**)

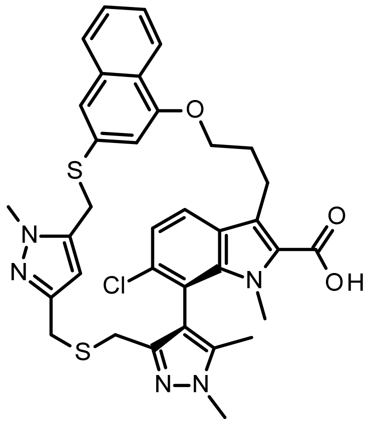

(R_a)-(+)-Methyl 17-chloro-5,13,14,22-tetramethyl-28-oxa-2,9-dithia-5,6,12,13,22-pentaazaheptacyclo[27.7.1.1^{4,7}.0^{11,15}.0^{16,21}.0^{20,24}.0^{30,35}]octatriaconta 1(37),4(38),6,11,14,16,18,20,23,29,31,33,35-tridecaene-23-carboxylate **A27** (1.87 g, 2.51

mmol) was dissolved in MeOH (8.35 mL), THF (8.35 mL) and water (8.35 mL). LiOH (0.90 g, 37.6 mmol) was added. The mixture was stirred for 4 h at RT. The mixture was concentrated to dryness. 2 N HCl (25 mL) was added. The aqueous phase was extracted with 5% MeOH in DCM (4 x 30 mL). The combined organic phases were washed with brine, dried over Na_2SO_4, filtered and concentrated. MeOH (20 mL) was added to the residue to result in a clear solution. This clear solution was concentrated to give a white solid which was dried under vacuum to give (R_a)-(+)-17-chloro-5,13,14,22-tetramethyl-28-oxa-2,9-dithia-5,6,12,13,22-pentaazaheptacyclo[27.7.1.1^{4,7}.0^{11,15}.0^{16,21}.0^{20,24}.0^{30,35}]octatriaconta-1(37),4(38),6,11,14,16,18,20,23,29,31,33,35-tridecaene-23-carboxylic acid **(R_a)-7** (1.55 g, 92.0%, >98.0% e.e.).

^{1}H NMR (400 MHz, DMSO-d_6) δ 1.97 (s, 3H), 2.20-2.30 (m, 1H), 2.35-2.50 (m, 1H), 2.90 (AB, d, J = 14.1 Hz, 1H), 3.07 - 3.19 (m, 3H), 3.40 - 3.47 (m, 2H), 3.50 (s, 3H), 3.71 (s, 3H), 3.76 (s, 3H), 3.86 (dd, J = 1.2, 8.8 Hz, 1H), 4.07 - 4.15 (m, 1H), 4.26 (s, 2H), 4.75 (s, 1H), 6.67 (s, 1H), 7.14 (d, J = 8.7 Hz, 1H), 7.38 (s, 1H), 7.45-7.52 (m, 2H), 7.71 (d, J = 7.2 Hz, 1H), 7.87 (d, J = 8.7 Hz, 1H), 8.10 (d, J = 7.2 Hz, 1H), 13.32 (br. s. 1H); ^{13}C NMR (126 MHz, DMSO-d_6) δ 9.62, 20.21, 26.72, 26.84, 28.46, 29.72, 33.32, 36.15, 36.22, 67.22, 104.31, 105.91, 112.62, 116.17, 120.88, 121.07, 121.40, 121.58, 121.77, 123.67, 125.48, 126.69, 126.91, 127.15, 128.66, 131.13, 131.92, 133.80, 137.19, 138.02, 138.95, 145.96, 146.12, 153.89, 163.35; m/z (ES$^+$), HRMS Calcd for $C_{35}H_{35}ClN_5O_3S_2$ [M+H]$^+$ 672.1864; found 672.1845.

Post Purification e.e. purity check:

Chiral analysis method: SFC: Chiralpak ID® column, 4.6 x 250 mm, 5 µm, Temperature = 40 °C, 40:60 MeOH:CO_2, UV detection at 220 nm, flow rate = 2.8 mL/min, Outlet Pressure = 100 bar, retention time of 7.33 min, >98.0% e.e., $[\alpha]_D$ +87° (c = 0.042, MeOH).

(S_a)-(–)-17-Chloro-5,13,14,22-tetramethyl-28-oxa-2,9-dithia-5,6,12,13,22-pentaazaheptacyclo[27.7.1.1^{4,7}.0^{11,15}.0^{16,21}.0^{20,24}.0^{30,35}]octatriaconta-1(37),4(38),6,11,14,16,18,20,23,29,31,33,35-tridecaene-23-carboxylic acid (**(S_a)-7**)

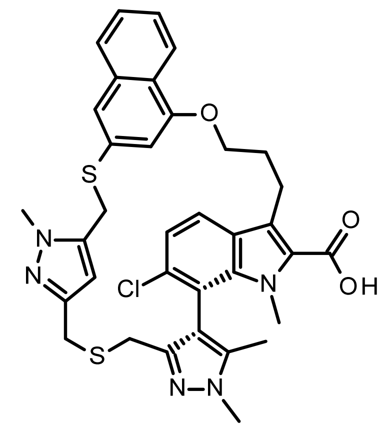

Starting from (S_a)-(-)-methyl 17-chloro-5,13,14,22-tetramethyl-28-oxa-2,9-dithia-5,6,12,13,22-pentaazaheptacyclo[27.7.1.$1^{4,7}$.$0^{11,15}$.$0^{16,21}$.$0^{20,24}$.$0^{30,35}$]octatriaconta 1(37),4(38),6,11,14,16,18,20,23,29,31,33,35-tridecaene-23-carboxylate **A28** (1.40 g, 2.04 mmol), the same procedure given for **(*R*$_a$)-7** was performed to obtain (S_a)-(-)-17-chloro-5,13,14,22-tetramethyl-28-oxa-2,9-dithia-5,6,12,13,22-pentaazaheptacyclo[27.7.1.$1^{4,7}$.$0^{11,15}$.$0^{16,21}$.$0^{20,24}$.$0^{30,35}$]octatriaconta-1(37),4(38),6,11,14,16,18,20,23,29,31,33,35-tridecaene-23-carboxylic acid **(*S*$_a$)-7** (1.25 g, 91.0%, 98.0% e.e.).

This (S_a)-(-)-17-chloro-5,13,14,22-tetramethyl-28-oxa-2,9-dithia-5,6,12,13,22-pentaazaheptacyclo[27.7.1.$1^{4,7}$.$0^{11,15}$.$0^{16,21}$.$0^{20,24}$.$0^{30,35}$]octatriaconta-1(37),4(38),6,11,14,16,18,20,23,29,31,33,35-tridecaene-23-carboxylic acid was further purified by chiral SFC (Chiralpak ID® column, 21 mm x 250 mm, Temperature = 40 °C, 40:60 EtOH:CO_2, UV detection @ 220 nm, loading= 60 mg/inj, conc = 33 mg/mL in MeOH, flow rate = 60 mL/min) three times to obtain **(*S*$_a$)-7** with >98.8% ee.

^{1}H NMR (400 MHz, DMSO-d_6) δ 1.97 (s, 3H), 2.20 - 2.30 (m, 1H), 2.35 - 2.50 (m, 1H), 2.90 (AB, d, J = 14.1 Hz, 1H), 3.07 - 3.19 (m, 3H), 3.40 - 3.47 (m, 2H), 3.50 (s, 3H), 3.71 (s, 3H), 3.76 (s, 3H), 3.86 (dd, J = 1.2, 8.8 Hz, 1H), 4.07 - 4.15 (m, 1H), 4.27 (s, 2H), 4.76 (s, 1H), 6.67 (s, 1H), 7.14 (d, J = 8.7 Hz, 1H), 7.38 (s, 1H), 7.45 - 7.52 (m, 2H), 7.71 (d, J = 7.2 Hz, 1H), 7.87 (d, J = 8.7 Hz, 1H), 8.10 (d, J = 7.2 Hz, 1H), 13.32 (br. s., 1H); ^{13}C NMR (126 MHz, DMSO-d_6) δ 9.62, 20.21, 26.72, 26.84, 28.46, 29.72, 33.32, 36.15, 36.22, 67.22, 104.31, 105.91, 112.62, 116.17, 120.88, 121.07, 121.40, 121.58, 121.77, 123.67, 125.48, 126.69, 126.91, 127.15, 128.66, 131.13, 131.92, 133.80, 137.19, 138.02, 138.95, 145.96, 146.12, 153.89, 163.35; m/z (ES$^+$), HRMS Calcd for $C_{35}H_{34}ClN_5O_3S_2$ [M+H]$^+$ 672.1864; found 672.1852.

<u>Post Purification e.e. purity check:</u>

Chiral analysis method: SFC: Chiralpak ID® column, 4.6 x 100 mm, 5 µm, Temperature = 40 °C, 40:60 EtOH:CO_2, UV detection at 220 nm, flow rate = 2.8 mL/min, Outlet Pressure = 100 bar, retention time of 2.61 min, >99.8% e.e., $[\alpha]_D$ –92º (*c* = 0.048, MeOH).

Conformational Analysis by NMR. NMR spectra were recorded on a Bruker 500 MHz equipped with a 5 mm QNP cryoprobe. NMR data was acquired in DMSO-d6 at 27 °C and chemical shifts referenced to the DMSO-d6 residual solvent signal (2.49 ppm). For the structural assignment the following NMR experiments were acquired, using the standard pulse sequences available in TopSpin 2.8 (Bruker GmbH): 1D ^1H, 2D COSY, ^1H-^{13}C HSQC and HMBC. For the conformational analysis of compound **7**, the 2D EASY ROESY experiment was recorded,[3] employing a zero-quantum filter element, 2048x256 data points in F2 and F1 respectively, a spectral width of 16 ppm, mixing time of 300 ms, and a relaxation delay of 5 s. NOE intensities were classified as STRONG, MEDIUM and WEAK. The resulting NMR-estimated distance restraints were fitted to ensembles of energy-minimized conformers (low-mode molecular dynamics; Maestro OPLS3 force field, version 10.7.015, Schrödinger; MOE Amber10 force field, version 2016.0802, CCG), using the MSpin NOE fitter algorithm.[4]

Supplementary Table 3: ^1H NMR assignment of **(*R*$_a$)-7**

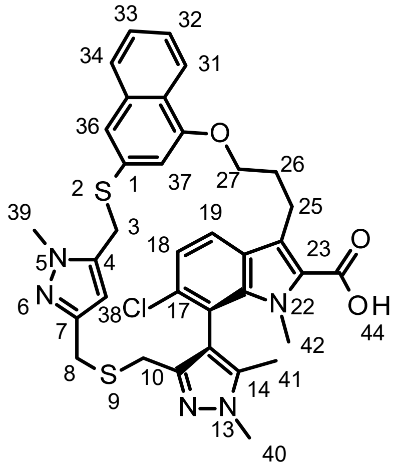

δ shift (ppm)	H's	mulitplet	J (Hz)	Atom
1.96	3	s	-	41
2.21	1	m	-	26"
2.35	1	m	-	26'
2.87	1	d	14.17	8"
3.05	1	ddd	14.23, 10.61, 3.89	25"
3.11	1	d	14.17	8'
3.17	1	d	12.63	10"
3.41	1	d	12.63	10'
3.43	1	m	-	25'
3.50	3	s	-	42
3.71	3	s	-	39
3.74	3	s	-	40
3.83	1	td	9.27, 4.96	27"
4.08	1	td	9.27, 6.84	27'
4.24	1	d	15.70	3"
4.27	1	d	15.70	3'
4.74	1	s	-	38
6.66	1	s	-	37
7.12	1	d	8.63	18
7.37	1	s	-	36

7.44	1	t	8.28	32
7.50	1	t	7.91	33
7.70	1	d	7.91	34
7.84	1	d	8.63	19
8.09	1	d	8.28	31
13.35	1	s	-	44

Supplementary Table 4: NOE correlations and inter-proton NOE estimates from 2D ROESY for **(*R*a)-7**

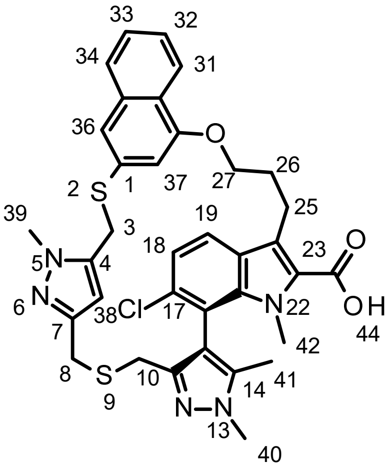

Strong NOE	Medium NOE	Weak NOE	No NOE
37-19	19-38	19-27”	38-26
37-38	19-3		8-39,40,41,42
38-18	19-27’		10-39,40,41,42
			38-31,32,33,34,36

Crystallization and structure determination

The crystal structure of compound **1** bound to Mcl-1 (PDB ID: 6FS2) was determined by the contract research organization Proteros (http://www.proteros.de) using their gallery system.

Co-crystals for compound **4** (PDB ID: 6FS1) were grown using a human-mouse chimera of Mcl-1[5] by sitting drop vapor diffusion. The protein was co-concentrated with the ligand at 2 mM (from a 200 mM DMSO stock) and Mcl-1 at 50 µM. The protein buffer was 20 mM Tris-HCl, 50 mM NaCl, 1 mM TCEP at pH 8 and the final concentration of the protein was around 500 µM. The concentrated solution was equilibrated after mixing 1:1 with a solution containing; 22% PEG MME 5K, 2% PEG 400, 0.1M MES pH 5.6. Crystals were cryo-protected using the well solution supplemented with 20% ethylene glycol and 2 mM compound before being flash frozen in liquid nitrogen. Data were collected using the APS synchrotron in Chicago. Diffraction data were processed with XDS[6] and scaled using SCALA/AIMLESS,[7] as implemented in the autoPROC routines from Global Phasing.[8] The structure was solved by molecular replacement and the protein and inhibitor were modeled into the electron density using Coot,[9] and refined using autoBUSTER.[10]

Co-crystals for compound **7** (PDB ID: 6FS0) were grown using the Mcl-1-FAb complex and a previously described method.[11] The compounds (100 mM stock in DMSO) were added to the complex to a final concentration of 1 mM. Small, thin plate like crystals were grown using a well

solution containing 18% w/v PEG-8000, 0.2M calcium acetate and 0.1M sodium acetate (pH 4.6) mixed in a protein to well ratio of 1:3. Crystals were cryo-protected using the well solution supplemented with 23% butan-2,3-diol and flash frozen in liquid nitrogen. The data set was collected at the Soleil synchrotron on beamline Proxima I and auto processed using Xia2[8]. The data were phased by molecular replacement using the program PHASER.[9] Structures were built and refined using Coot and autoBUSTER.

Supplementary Table 5: Crystallographic data and refinement statistics for Mcl-1:ligand crystal structures for compounds **1**, **4** and **7**.

Compound (pdb deposition)	**1** (6FS2)	**4** (6FS1)	**7** (6FS0)
Space Group	*P1*	*P 1 21 1*	*C2*
Unit cell dimensions (Å)	a = 46.7 Å<br>b = 46.7 Å<br>c = 47.5 Å<br>α = 74.7°<br>β = 74.7°<br>γ = 61.8°	a = 42.7 Å<br>b = 64.5 Å<br>c = 50.0 Å<br>α = γ = 90°<br>β = 98.8°	a = 147.2 Å<br>b = 42.0 Å<br>c = 107.3 Å<br>α = γ = 90°<br>β = 113.2°
Wavelength (Å)	1.5406	1.5406	0.97858
Resolution (Å)	45.2-2.6	64.5-1.6	49.32-2.24
Number of reflections	21139	109246	108756
Unique reflections	10721	34104	29214
Multiplicity	2(2)	3.2 (1.6)	3.7 (3.3)
Completeness (%)	97.2(96)	79.8 (18)	99.4 (93.8)
<I/σI>	8.55(2.1)	26 (6.3)	9 (1.7)
$R_{merge}{}^{a}$ (%)	8.1(40.0)	2.7 (9.0)	9.3 (68.4)
$R_{work}{}^{b}$ / $R_{free}{}^{c}$ (%)	20.8/26.9	19.9/22.6	22.4/25.0
Ramachandran Parameters			
Prefered (%)	99.5	97.9	95.9
Allowed (%)	99.3	100	98.7

1 Elmore, S. W. *et al.* 7-Substituted indoles as Mcl-1 protein inhibitors and their preparation. WO2008131000A2 (2008).

2 Bruncko, M. *et al.* Structure-guided design of a series of MCL-1 inhibitors with high affinity and selectivity. *J Med Chem* **58**, 2180-2194, doi:10.1021/jm501258m (2015).

3 Thiele, C. M., Petzold, K. & Schleucher, J. EASY ROESY: reliable cross-peak integration in adiabatic symmetrized ROESY. *Chemistry* **15**, 585-588, doi:10.1002/chem.200802027 (2009).

4 Troche-Pesqueira, E., Anklin, C., Gil, R. R. & Navarro-Vazquez, A. Computer-Assisted 3D Structure Elucidation of Natural Products using Residual Dipolar Couplings. *Angew Chem Int Ed Engl* **56**, 3660-3664, doi:10.1002/anie.201612454 (2017).

5 Czabotar, P. E. *et al.* Structural insights into the degradation of Mcl-1 induced by BH3 domains. *Proc Natl Acad Sci U S A* **104**, 6217-6222, doi:10.1073/pnas.0701297104 (2007).

6 Kabsch, W. Xds. *Acta Crystallogr D Biol Crystallogr* **66**, 125-132, doi:10.1107/S0907444909047337 (2010).
7 Evans, P. R. & Murshudov, G. N. How good are my data and what is the resolution? *Acta Crystallogr D Biol Crystallogr* **69**, 1204-1214, doi:10.1107/S0907444913000061 (2013).
8 Vonrhein, C. *et al.* Data processing and analysis with the autoPROC toolbox. *Acta Crystallogr D Biol Crystallogr* **67**, 293-302, doi:10.1107/S0907444911007773 (2011).
9 Emsley, P., Lohkamp, B., Scott, W. G. & Cowtan, K. Features and development of Coot. *Acta Crystallogr D Biol Crystallogr* **66**, 486-501, doi:10.1107/S0907444910007493 (2010).
10 BUSTER v. 2.8.0 (www.globalphasing.com, 2009).
