# Supplementary material for: Discovery of Mcl-1-specific inhibitor AZD5991 and preclinical activity in multiple myeloma and acute myeloid leukemia
Source: Nat Commun. 2018 Dec 17;9:5341. doi: 10.1038/s41467-018-07551-w (PMC6297231; doi:10.1038/s41467-018-07551-w)
Supplement: Supplementary file 2 — Description of Additional Supplementary Files [file 41467_2018_7551_MOESM2_ESM.docx]

**Description of Additional Supplementary Files**

**File Name**: Supplementary Data 1

**Description**: AZD5991 activity in cancer cell lines. Cancer cell lines were treated with a range of concentrations of AZD5991 and cell growth and caspase 3/7 activation were assessed by CellTiter Glo and CaspaseGlo, respectively. Data analysis was performed using GraphPad Prism. Data shown are representative of ≥ 2 independent experiments.
